# Tetrahedral Cu(I) Complexes for Thermally Activated Delayed Fluorescence: A Density Functional Benchmark Study with QM/MM Models

**DOI:** 10.1021/acs.inorgchem.5c00761

**Published:** 2025-04-30

**Authors:** Toni Eskelinen, Antti J. Karttunen

**Affiliations:** Department of Chemistry and Materials Science, School of Chemical Engineering, Aalto University, Kemistintie 1, Espoo 02150, Finland

## Abstract

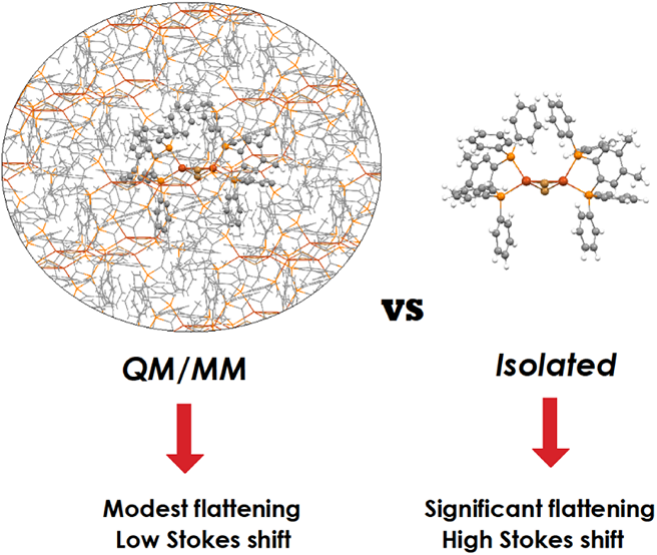

Tetrahedral Cu(I) complexes represent a major class of organometallic thermally activated delayed fluorescence (TADF) emitters. However, due to the d^10^ electronic structure and low-lying metal-to-ligand charge transfer (MLCT) states, these systems exhibit (pseudo) Jahn–Teller distortions in the excited state, resulting in tetrahedral to square planar geometry flattening. From a computational point of view, this poses a major challenge since theoretical studies are often conducted with isolated single molecule models. Such models are incapable of describing the suppressing effect of the surrounding solid-state environment on geometry relaxation and often result in overly relaxed excited-state geometries and inaccurate transition energies. Crystal models based on quantum mechanics/molecular mechanics (QM/MM) approaches have emerged as viable candidates for modeling the solid-state environment. Here, we report a study, conducted on 56 experimentally known tetrahedral Cu(I) TADF emitters, comparing the isolated and QM/MM models together with five commonly used density functionals. Our results show that while differences in ground-state geometries and excitation energies are small, significant deviations are observed in the excited-state geometries and fluorescence energies. Because of the added rigidity, the QM/MM models show less (pseudo) Jahn–Teller effect induced geometry flattening, which consequently results in blue-shifted fluorescence energies compared with isolated models.

## Abstract 2

A series of tetrahedral Cu(I) thermally activated delayed fluorescent emitter molecules were investigated at the density functional level of theory using both isolated and QM/MM models. The increased rigidity provided by the QM/MM model effectively reduced the Jahn−Teller effect-induced geometry flattening experienced in the excited state. As a result, improved correlation between experimental and calculated fluorescence energies is obtained.

## Introduction

Luminescent molecular materials can be utilized in a wide variety of applications, such as optoelectronic devices,^1−3^ photocatalysis,^4,5^ chemical sensing,^6,7^ and photodynamic therapy.^8,9^ Especially, the field of organic light-emitting diodes (OLEDs) has gained significant academic and industrial interest over the past decades due to the possibility of producing thin displays with high contrast ratios or other intriguing properties, such as bendability or transparency.^10−12^ Early emitter materials in OLEDs, or other electroluminescent devices, were based on materials operating solely on fluorescence.^13,14^ However, the efficiency of these devices suffers greatly from the restriction imposed by spin statistics as the singlet and triplet excitons are formed in a 1:3 ratio, resulting in a maximum internal quantum efficiency (IQE) of only 25%.^15,16^ A significant increase in efficiency is achieved with phosphorescent emitter molecules, which can convert the singlet excitons to triplets via intersystem crossing (ISC), followed by radiative decay from the triplet state, thus allowing both singlet and triplet excitons to be utilized in light generation.^17−19^ In order to make the phosphorescence process efficient, however, one has to use compounds incorporating heavy elements with strong spin–orbit coupling (SOC), such as Ir(III)^20,21^ or Pt(II)^22,23^ -based transition metal complexes. The high cost and low abundance of these metals greatly limit large-scale commercial usage.

Promising alternatives with comparable efficiencies for phosphorescent emitters are molecules exhibiting thermally activated delayed fluorescence (TADF), also known as E-type delayed fluorescence. TADF emitters can be made with all-organic molecules or with organometallic complexes based on low-cost and earth-abundant metals.^24−26^ TADF emitters operate by minimizing the energy difference between the first excited singlet and triplet states, Δ*E*(S_1_ – T_1_), which allows for reverse intersystem crossing (rISC) between the excited triplet and singlet states by means of thermal energy at room temperature, followed by delayed fluorescence from the singlet excited state. A minimum requirement for TADF is achieving a low enough singlet–triplet energy difference. As this energy difference is proportional to the quantum mechanical exchange energy (which is proportional to the overlap density between the ground and excited states), one way of achieving low Δ*E*(S_1_ – T_1_) is by having low-lying excited states with charge transfer (CT) character.^27,28^

**Figure 1:**
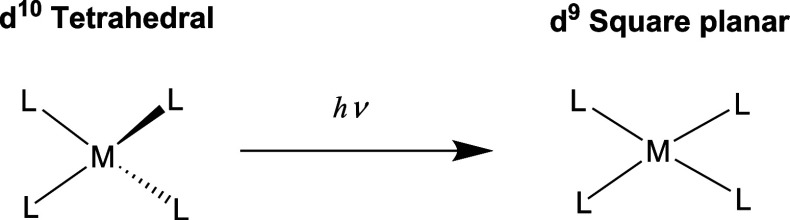
Schematic illustration of the pseudo Jahn–Teller distortion occurring in tetrahedral d^10^ complexes upon MLCT excitation.

Among the organometallic TADF emitter compounds, Cu(I) complexes represent perhaps the most documented class of compounds, with the earliest example reported in the early 1980s by McMillin et al.^29^ Cu(I) complexes commonly display low-lying metal-to-ligand charge transfer (MLCT) states, resulting in small Δ*E*(S_1_ – T_1_).^30,31^ While the coordination geometry for Cu(I) is somewhat flexible, tetrahedral is the preferred geometry.^32^ The combination of tetrahedral geometry and MLCT transition results in a flattening distortion in the excited state, caused by the (pseudo) Jahn–Teller effect, where the coordination geometry becomes twisted toward a square planar orientation (Figure 1).^33,34^ These flattening distortions often result in red-shifted emission and decreased luminescence efficiency, especially in a fluid medium, as the more distorted geometry generally leads to an increased rate of nonradiative relaxation. However, in a solid-state environment, highly efficient emitter compounds based on Cu(I) complexes can be realized because the flattening distortions are largely restricted in the aggregated state.^35^ The degree of structural relaxation can be further restrained by utilizing sterically demanding substituents.^30,36,37^

From a computational point of view, modeling solid-state tetrahedral Cu(I) TADF emitters is far from trivial since most computational studies are performed with models consisting of just a single isolated molecule in a vacuum, with or without an implicit solvent layer. Such models are obviously incapable of describing the structural rigidity experienced in the aggregated state. This can lead to overly relaxed excited-state geometries and, consequently, highly inaccurate emission energies. Due to this, the theoretical analysis of tetrahedral Cu(I) TADF emitters is often limited to ground-state properties, and/or the excited-state properties are extrapolated based on the ground-state geometries. Recently, some studies have utilized a quantum mechanics/molecular mechanics (QM/MM)-based approach, where a supercell is formed from the experimental crystal structure, and one molecule inside the supercell is assigned as the QM layer and described with density functional theory (DFT), whereas the surrounding molecules are described with a simple MM force field.^38−40^ In a study conducted by Cui et al., the authors studied two tetrahedral Cu(I) TADF emitters bearing bidentate NN and PP ligands, [Cu(NN)(PP)], where NN = 5-(2-pyridyl)-tetrazole, PP = bis[2-(diphenylphosphino)phenyl]ether (DPEPhos) or bis[2-(diphenylphosphino)p-tolyl]ether.^41^ They studied the materials in gas, solution, and solid phases with the B3LYP density functional method, utilizing three different model systems: an isolated molecule in a vacuum, an isolated molecule in an implicit solvent, and a QM/MM crystal. Their results showed that the degree of structural relaxation in the excited state occurs to a significantly greater extent in the gas phase or solution than in the solid state. This is best reflected in the angle between the two planes spanned by the coordinating nitrogen or phosphorus atoms and the copper ion (N–Cu–N, P–Cu–P). In the ground state, all three models predicted roughly the same angles between the two planes, with less than 3.5° deviation between the three models at most. The change in this angle between the ground and excited (S_1_) state geometries was on average 27° for the gas-phase models, 31° for the solution models, and 14° for the QM/MM models. In other words, the excited-state structures were significantly flatter for the gas and solution models. This indicates that the QM/MM model provides a more rigid environment and can restrict the extent of structural relaxation caused by the Jahn–Teller effect. Similar observations were made in a recent study by Zou et al., where they studied cationic [Cu(NN)(PP)]^+^, where NN = 3-(2-pyridyl)pyrazole derivative, PP = bis[2-(diphenylphosphino)phenyl]ether, in solution and solid state using implicit solvent and QM/MM models together with the PBE0 hybrid density functional method.^42^

To the best of our knowledge, no large-scale study assessing the accuracy of QM/MM models in predicting transition energies has been conducted to date. Motivated by this, we performed a time-dependent DFT (TD-DFT) benchmark study on a total of 56 tetrahedral Cu(I) TADF emitters found in the literature. Five commonly used density functional methods (PBE0, B3LYP, LRC-ωPBEh, CAM-B3LYP, and ωB97X) from the families of hybrid and range-separated hybrid functionals were used together with both isolated and QM/MM models.

### Studied Emitters

**Figure 2:**
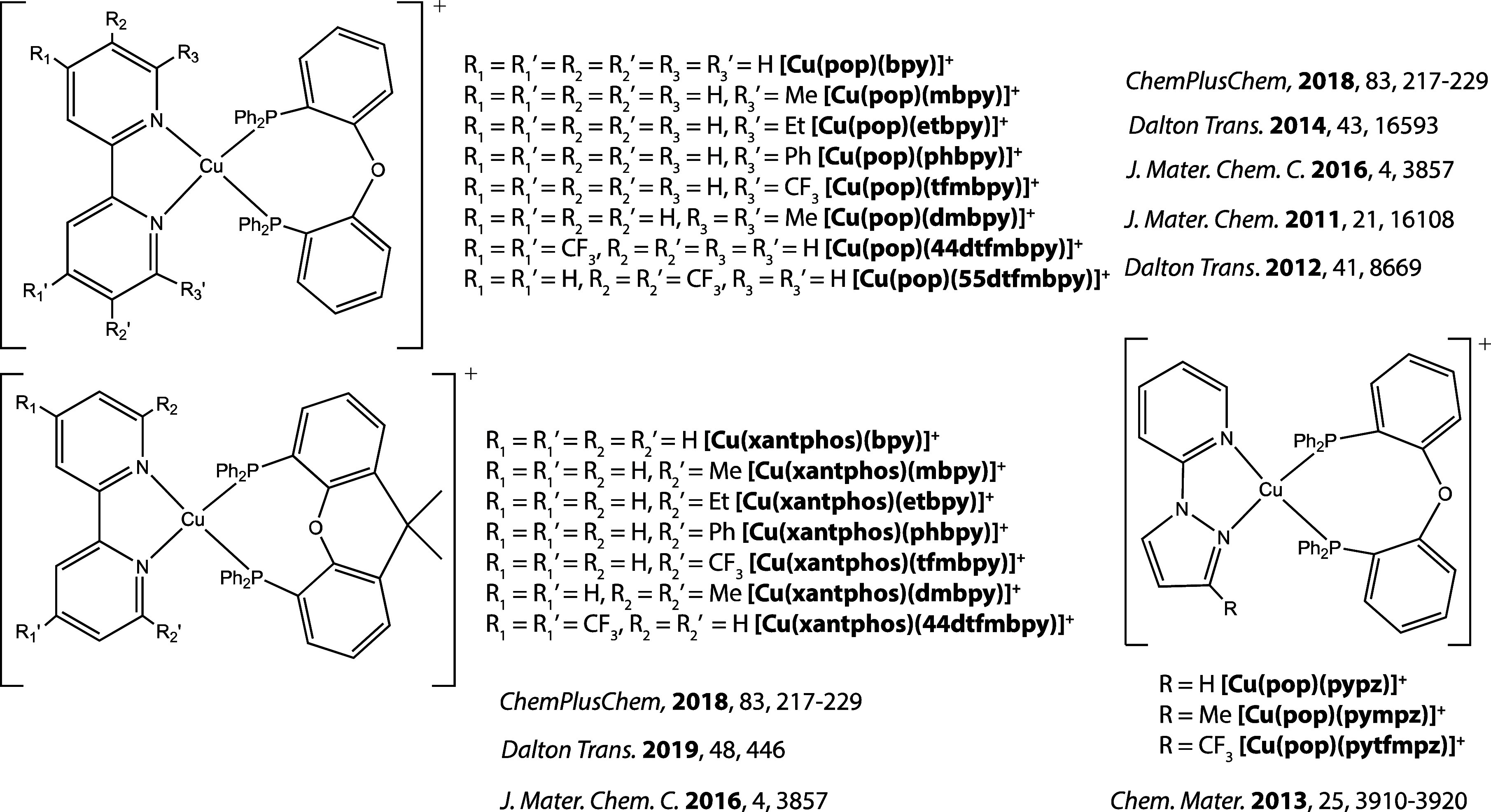
[Cu(NN)(PP)]^+^ emitters included in the study.^43−49^.

**Figure 3:**
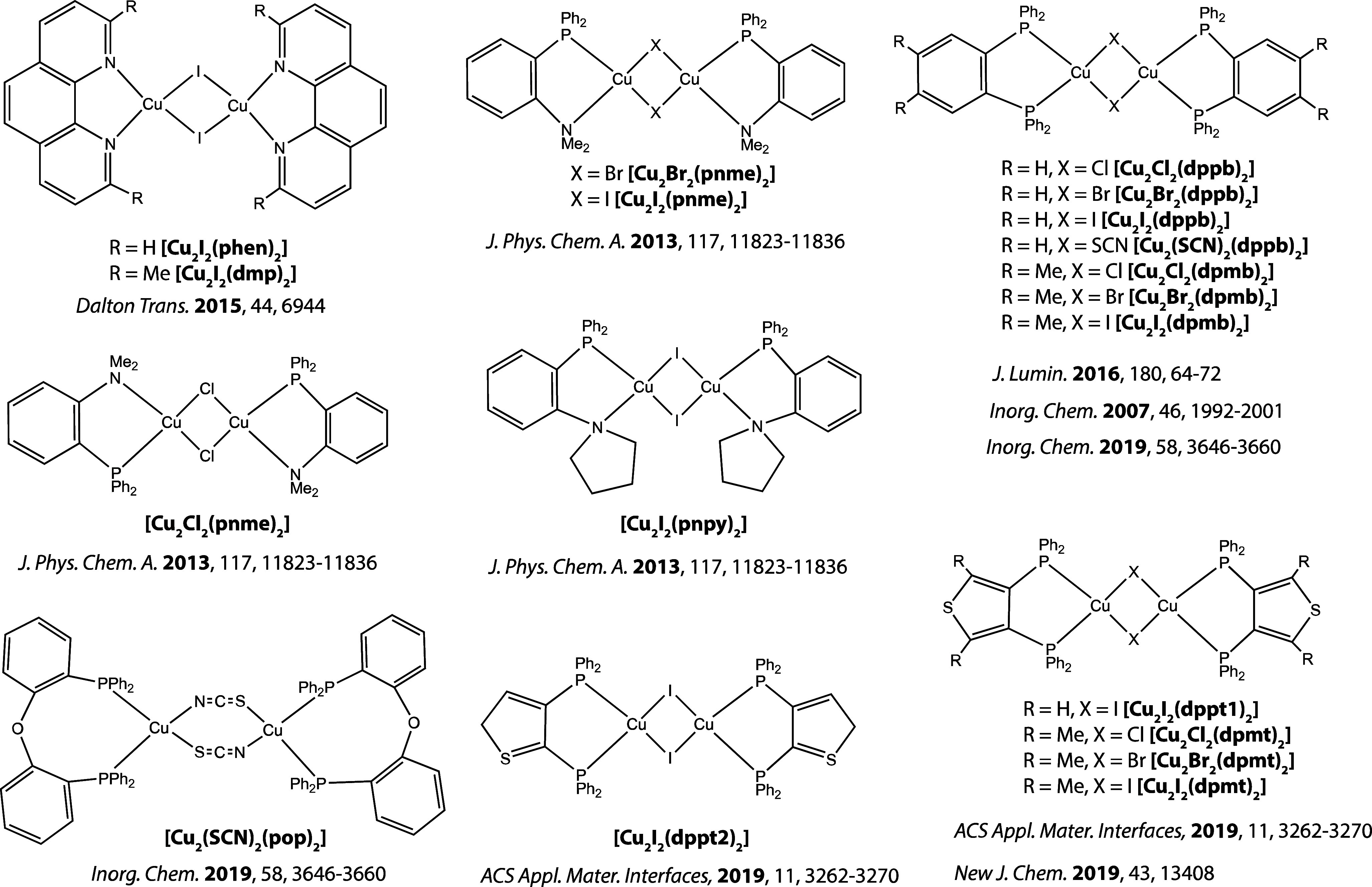
[Cu_2_X_2_(L^2^)_2_] emitters included in the study.^50−56^.

**Figure 4:**
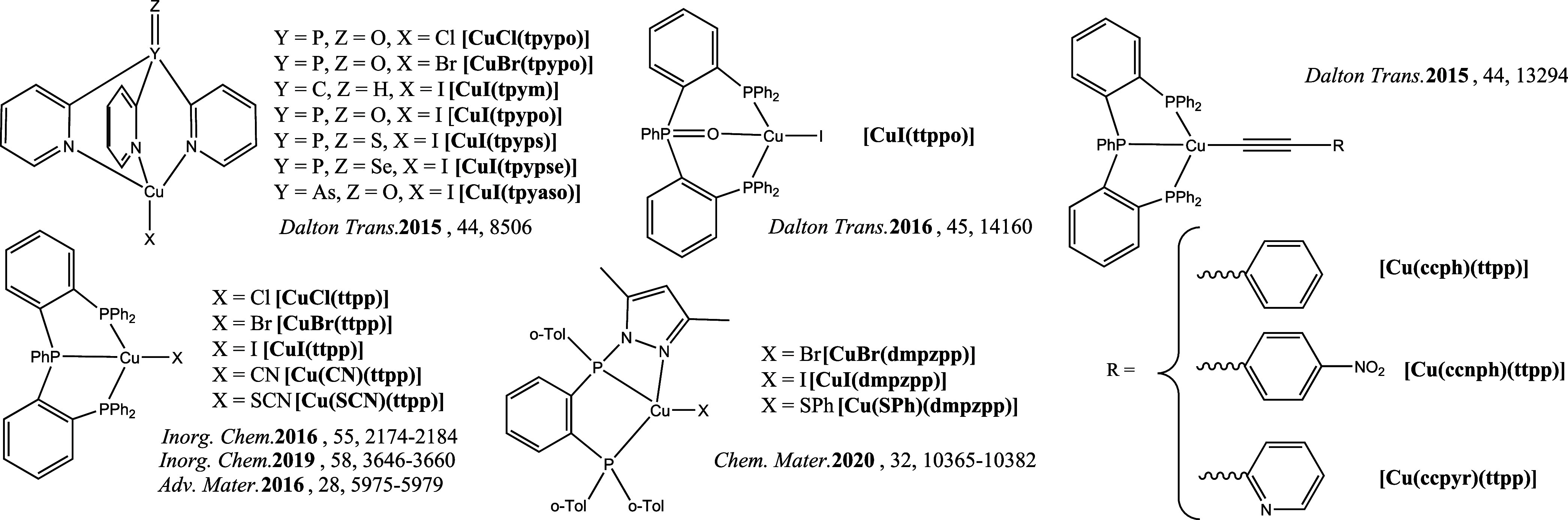
[CuX(L^3^)] emitters included in the study.^56−62^.

We included a total of 56 tetrahedral Cu(I) TADF emitters in the present study, all of which have been previously characterized in the literature. This set includes 18 cationic complexes of the general formula [Cu(NN)(PP)]^+^ (where NN and PP indicate bidentate chelating ligands with nitrogen and phosphorus as the coordinating atoms, respectively, Figure 2), 19 binuclear complexes of the type [Cu_2_X_2_(L^2^)_2_] (X = bridging halide or pseudohalide, L^2^ = bidentate chelating ligand with N or P as the coordinating atoms, Figure 3), and 19 complexes of the type [CuX(L^3^)] (where X = halide, pseudohalide or alkynyl ligand and L^3^ = tridentate chelating ligand which can contain P, N, or O as the coordinating atoms, Figure 4). All compounds included in the study are presented in Figures 2–4, and their photophysical properties are collected in Table S1.

## Computational Details

All electronic structure calculations were performed with the ORCA software package (version 5.0.3).^63^ Each set of calculations was conducted using five different density functionals: the hybrid density functionals PBE0^64,65^ and B3LYP,^66,67^ as well as the range-separated hybrid functionals LRC-ωPBEh,^68^ CAM-B3LYP,^69^ and ωB97X.^70^ No optimization of the range-separation parameter ω was done. Unless stated otherwise, all calculations utilized the def2-TZVP basis set together with the corresponding effective core potential (ECP) for heavy atoms (*Z* > 36).^71^ The resolution-of-the-identity and the chain-of-spheres for exchange (RIJCOSX) algorithms were used to reduce the computational burden in all calculations.^72,73^ TD-DFT calculations employed the Tamm–Dancoff approximation (TDA)^74^ due to the triplet instability problem associated with pure TD-DFT.^75−77^

**Figure 5:**
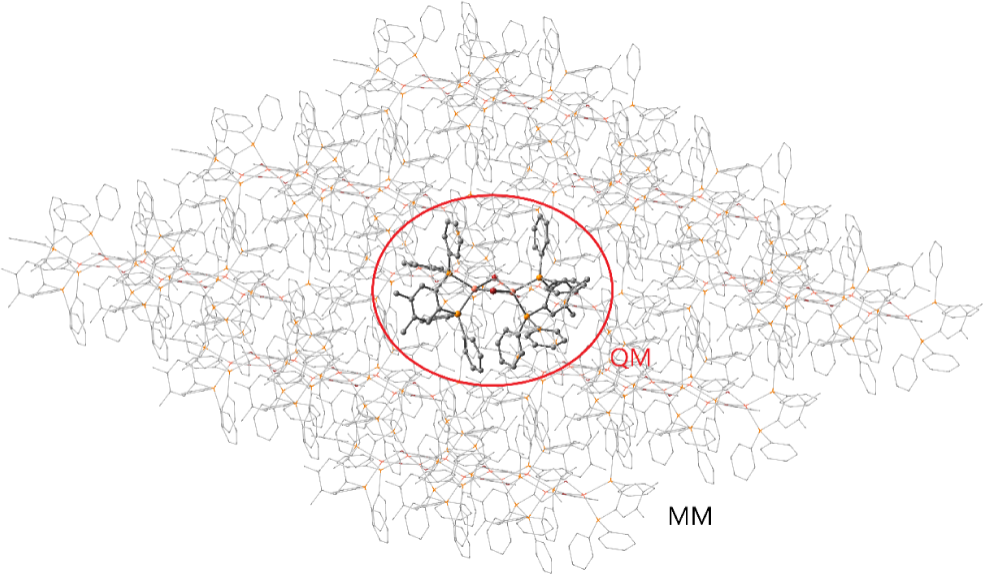
Example of the used QM/MM model as that used for **[Cu**_**2**_**Br**_**2**_**(dpmb)**_**2**_**]**. Hydrogens omitted for clarity.

Geometry optimizations were performed using two different models: an isolated model, consisting of a single molecule in a vacuum, and a QM/MM model to represent a molecule within a solid-state environment. The QM/MM models were built by first expanding the experimental crystal structures in three dimensions to form a supercell. From the middle of the generated supercell, one molecule (or cation in the case of ionic systems) was selected and assigned as the QM layer, whereas the surroundings were assigned as the MM layer (Figure 5). During the optimization, the MM layer was kept frozen, and only the QM part was allowed to relax. In this way, the purpose of the MM layer is only to increase the rigidity of the system by limiting the nuclear degrees of freedom and forcing the molecule to locate a stationary point in a more restricted space, thereby mimicking the behavior the molecule would exhibit in an experimental solid-state environment. The QM part was described with (TD-)DFT, and the MM layer was described with a simple force field generated with the makeff utility program within ORCA’s MM module. While freezing of the MM layer is necessary to obtain reasonable geometries (due to the simplistic nature of the force field used and the lack of parametrization for organometallic species in more advanced force fields), one could utilize a three-layer QM/QM2/MM scheme, where both QM and QM2 layers are allowed to relax, to provide more degrees of freedom for the QM layer. In this approach, the QM2 layer would be described with a computationally more affordable method compared to the QM layer. To this end, we also experimented with a DFT/GFN1-xTB^78^/MM model, where the DFT layer consisted of one molecule in the center of the supercell, the GFN1-xTB layer represented the neighboring molecules relative to the DFT layer, and the MM layer included all other molecules in the supercell. In this model, both the DFT and GFN1-xTB layers were allowed to relax during the optimization. However, even with the smallest systems in the test set, we observed roughly a factor of 10 increase in computational time in finding a stationary point. Furthermore, with the larger molecules included in the test set, the increasing size of the active layer resulted in a bottleneck. Consequently, we only utilized the 2-layer QM/MM models. Mechanical embedding was used in all QM/MM calculations; that is, the MM layer could not polarize the QM region. Although electrostatic embedding would result in a more realistic description of solid-state solvation effects, the purpose of this study is primarily to investigate the degree of structural difference between an isolated single-molecule model and a model that can mimic the rigidity of a solid-state system, specifically regarding Jahn–Teller effect-induced flattening distortion and its outcome on predicted transition energies. Therefore, comparisons between different embedding approaches are out of scope for this study. For each system, we optimized the ground state (S_0_) and first excited singlet (S_1_) and triplet (T_1_) state geometries. Ground-state optimizations were done at the DFT level, and TD-DFT was used for all excited-state optimizations. To compare the optimized ground-state geometries against experimental structures as well as the optimized ground-state geometries against excited-state geometries, we used the root-mean-square deviation (RMSD) as a simple metric for structural deviation. After the two structures were superimposed, RMSD was calculated^79−81^ according to
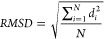
where *N* is the total number of atoms and *d*_*i*_ is the distance between atom *i* in the two geometries.

Following the geometry optimizations, single-point TD-DFT calculations were performed for each optimized structure to estimate the excitation and emission energies. In the single-point TD-DFT calculations, we used the scalar relativistic ZORA Hamiltonian^82,83^ with the perturbative inclusion of spin–orbit coupling (SOC) effects.^84^ The relativistically recontracted ZORA-def2-TZVP basis set was used for all elements with *Z* ≤ 36, and the all-electron SARC-ZORA-TZVP basis set was employed for heavier elements.^85^ For the QM/MM optimized models, the MM layer was discarded in the excitation and emission calculations, since in the mechanical embedding formalism, the MM layer cannot polarize the QM region and is therefore redundant in the single-point TD-DFT calculations.

To assess the predictive accuracy of each density functional, as well as the model, against the experimentally determined photophysical properties, we report the mean absolute deviation (MAD) and mean signed deviation (MSD) for S_0_ → S_1_ excitation energies and S_1_ → S_0_ fluorescence energies. In the experimental excitation data, there are some variations in the experimental setups, with some papers reporting excitation data measured in the solid state, whereas others only reported the absorption spectra in solution (and a few did not report any). In cases where the experimental data were obtained in solution, we also performed the TD-DFT calculations in solution to allow for a more reasonable comparison between experiment and theory. Calculations in solution were conducted only using the isolated models. To this end, we utilized the conductor-like polarizable continuum (C-PCM) implicit solvation method together with the same solvent as in the experimental conditions.^86^

## Results and Discussion

### Ground-State Structures

**Figure 6:**
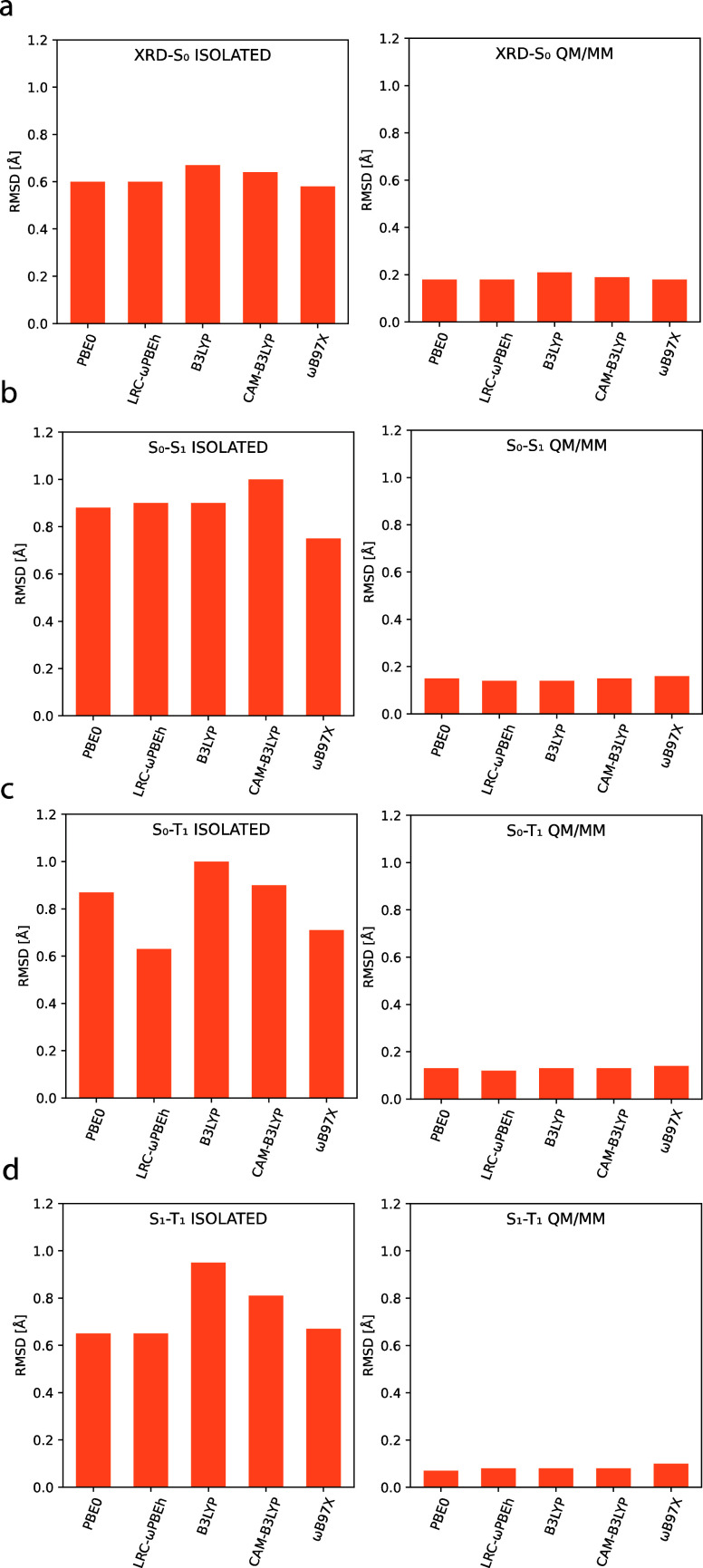
Average RMSD (*N* = 56) between superimposed XRD-S_0_ (a), S_0_-S_1_ (b), S_0_-T_1_ (c), and S_1_-T_1_ (d) structures with the isolated and QM/MM models.

We started by comparing the optimized ground-state structures with isolated and QM/MM models to the experimental X-ray diffraction (XRD) structures. The average RMSD values between the optimized and experimental geometries for the three groups of complexes are collected in Table 1. More detailed data are available in Tables S2 and S3. Of the tested density functionals, ωB97X predicts geometries closest to the experimental ones using the isolated models, resulting in an average RMSD value of 0.58 Å, whereas the B3LYP functional shows the largest deviation with an average RMSD of 0.67 Å. With the QM/MM models, however, the difference between the tested functionals is mostly negligible, with PBE0, LRC-ωPBEh, and ωB97X reaching an average RMSD of 0.18 Å, followed by CAM-B3LYP and B3LYP with RMSD values of 0.19 Å and 0.21 Å, respectively. From the RMSD values, one can observe that the QM/MM models are able to reproduce the experimental geometries with far better accuracy (Figure 6a).

**Table 1:** Average RMSD (Å) between XRD and Optimized Ground-State Structures^a^

	PBE0	LRC-ωPBEh	B3LYP	CAM-B3LYP	ωB97X
ISOLATED					
[Cu(NN)(PP)]^+^	0.50	0.49	0.52	0.50	0.56
[Cu_2_X_2_(L^2^)_2_]	0.86	0.88	1.03	0.95	0.75
[CuX(L^3^)]	0.43	0.43	0.44	0.44	0.44
**All**	**0.60**	**0.60**	**0.67**	**0.64**	**0.58**
QM/MM					
[Cu(NN)(PP)]^+^	0.19	0.18	0.21	0.19	0.18
[Cu_2_X_2_(L^2^)_2_]	0.21	0.21	0.26	0.23	0.20
[CuX(L^3^)]	0.15	0.15	0.17	0.16	0.16
**All**	**0.18**	**0.18**	**0.21**	**0.19**	**0.18**

a *N* = 56.

**Figure 7:**
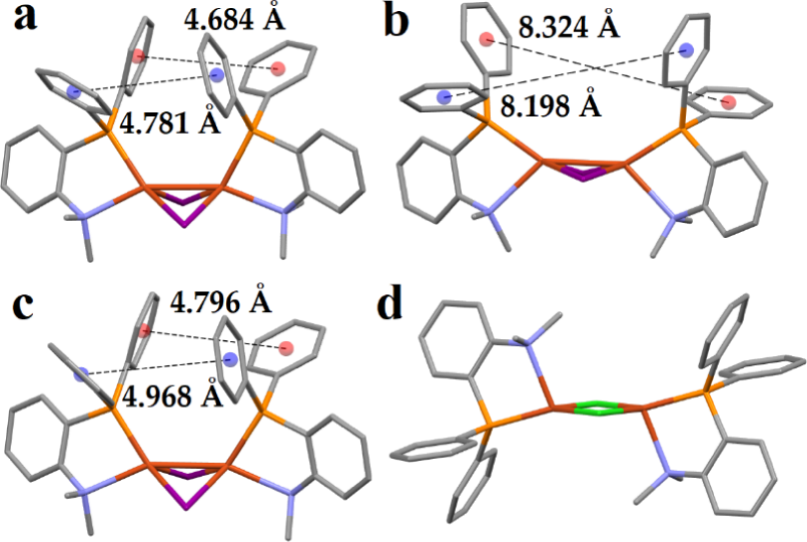
XRD (a) and LRC-ωPBEh optimized structures of **[Cu**_**2**_**I**_**2**_**(pnme)**_**2**_**]** with the isolated (b) and QM/MM (c) models and the XRD structure of **[Cu**_**2**_**Cl**_**2**_**(pnme)**_**2**_**]** (d). Hydrogens omitted for clarity.

To identify the key structural differences between the isolated and QM/MM models, we took a closer look at the compounds that resulted in the biggest RMSD values between the experimental and theoretical geometries. Of the entire test set, **[Cu**_**2**_**I**_**2**_**(pnme)**_**2**_**]**, **[Cu**_**2**_**Br**_**2**_**(pnme)**_**2**_**]** and **[Cu**_**2**_**I**_**2**_**(pnpy)**_**2**_**]** showed the largest discrepancy, with RMSD values between the X-ray and optimized (isolated models) ranging from 1.45 Å to 1.88 Å (Table S2) with four out of the five functionals used. Closer inspection of the crystal structures reveals the likely reason behind the high RMSD values. Due to the *cis* configuration of the (P^N) ligands, close contacts are observed between the phenyl groups of adjacent (P^N) ligands. For example, in **[Cu**_**2**_**I**_**2**_**(pnme)**_**2**_**]** centroid-to-centroid distances of 4.684 and 4.781 Å between the phenyl groups are measured (Figure 7a). This likely induces a fair amount of steric repulsion in the system and leads to significant geometry rearrangement in the optimized structures. Indeed, with the LRC-ωPBEh functional centroid-to-centroid distances of 8.324 and 8.198 Å are predicted between the same phenyl groups (Figure 7b) with similar distances also predicted with the PBE0, B3LYP and CAM-B3LYP functionals. Interestingly, ωB97X is the only functional that results in reasonable RMSD between the X-ray and isolated models, with RMSD values of 0.66 0.44, and 0.81 Å obtained for **[Cu**_**2**_**I**_**2**_**(pnme)**_**2**_**]**, **[Cu**_**2**_**Br**_**2**_**(pnme)**_**2**_**]** and **[Cu**_**2**_**I**_**2**_**(pnpy)**_**2**_**]**, respectively. Inspection of the optimized structure of **[Cu**_**2**_**I**_**2**_**(pnme)**_**2**_**]** reveals centroid-to-centroid distances of 4.825 and 4.799 Å between the phenyl groups, which are close to the experimental values. This might be caused by the ωB97X functionals superior description of noncovalent interactions. However, with the QM/MM models, all functionals predict geometries close to the experimental ones, with RMSD values ranging from 0.16 to 0.31 Å for the three complexes in question, due to the added rigidity of the model (Table S3 and Figure 7c). Contrary to **[Cu**_**2**_**I**_**2**_**(pnme)**_**2**_**]** and **[Cu**_**2**_**Br**_**2**_**(pnme)**_**2**_**]**, the chloride-bridged isologue, **[Cu**_**2**_**Cl**_**2**_**(pnme)**_**2**_**]**, shows *trans* configurations for the (P^N) ligands (Figure 7d), resulting in far less steric repulsion. Consequently, the optimized structures also show less deviation with RMSD values ranging from 0.56 to 0.84 Å using the isolated models (Table S2).

**Figure 8:**
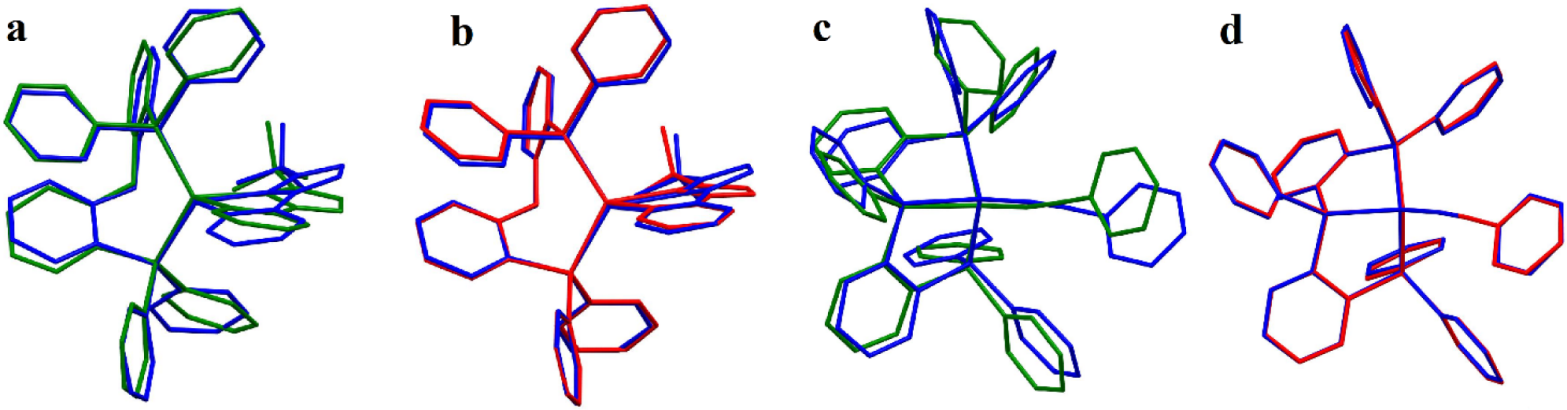
Superimposed X-ray (blue) and LRC-ωPBEh optimized (isolated = green, QM/MM = red) structures for **[Cu(pop)(pytfmpz)]**^**+**^ (a, b) and **[Cu(ccpyr)(ttpp)]** (c, d). Hydrogens omitted for clarity.

In addition to geometry relaxation due to steric repulsion, freely rotating groups are the second major source of disparity for the isolated models. Among the [Cu(NN)(PP)]^+^ and [CuX(L^3^)] groups, **[Cu(pop)(pytfmpz)]**^+^ and **[Cu(ccpyr)(ttpp)]** show the highest deviation between experimental and optimized geometries, with RMSD values ranging from 0.55 to 0.84 Å and 0.91 to 1.14 Å, respectively (Table S2). Superimposed structures between experimental and optimized geometries are shown in Figure 8. Comparing the isolated and QM/MM models of **[Cu(pop)(pytfmpz)]^+^**, one can observe that in the isolated model, the phenyl groups of the pop ligand are clearly mismatched with respect to the X-ray structure, in addition to the slight bending of the N^N chelating ligand (Figures 8a, b). In the QM/MM model, however, only a slight mismatch in the pop ligand is noticeable. This effect is even more obvious for **[Cu(ccpyr)(ttpp)]** where in addition to the phenyl groups of the ttpp ligand, the alkynyl ancillary ligand is also rotated in the isolated model but matches the experimental geometry very well in the QM/MM model (Figures 8c, d).

### Excitation Energies

Next, we briefly assessed the predicted excitation energies. The statistical data are collected in Table 2, while data for individual complexes are provided in Tables S4 and S5. Note that of the 56 complexes included in this study, experimental excitation data were only available for 43 complexes. Furthermore, only 15 of those were obtained in the solid state, while 28 were obtained in solution. As the QM/MM models are intended to represent the solid-state environment, comparisons against experimental data were conducted only for those 15 complexes, whereas the isolated models were used to compare against experimental data obtained in both the solid state and solution. Overall, the hybrid functionals were able to produce excitation energies closer to the experimental values than the range-separated hybrid functionals, with PBE0 and B3LYP functionals resulting in MADs of 0.36 (0.38) eV and 0.48 (0.52) eV with the isolated (QM/MM) models, respectively. However, the hybrid functionals tend to underestimate the transition energies, which is reflected in the negative MSDs, with PBE0 producing an MSD of −0.18 (−0.24) eV and B3LYP producing an MSD of −0.39 (−0.44) eV with the isolated (QM/MM) models, respectively. Their range-separated counterparts, LRC-ωPBEh and CAM-B3LYP, produce slightly more erroneous excitation energies, resulting in MADs of 0.57 (0.52) eV and 0.64 (0.57) eV, respectively. Furthermore, both of these range-separated hybrids overestimate the excitation energy with respect to experimental values for each system. Out of the five functionals utilized, the range-separated hybrid ωB97X produced by far the most erroneous excitation energies, with an MAD and MSD of 1.03 (0.99) eV using the isolated (QM/MM) models.

**Table 2:** Mean Absolute Deviations (MAD) and Mean Signed Deviations (MSD) against Experimental Solid-State Data for the S_0_ → S_1_ Excitation Energies^a^^b^

	ISOLATED		QM/MM	
	MAD	MSD	MAD	MSD
PBE0	0.36	–0.18	0.38	–0.24
LRC-ωPBEh	0.57	0.57	0.52	0.52
B3LYP	0.48	–0.39	0.52	–0.44
CAM-B3LYP	0.64	0.64	0.57	0.57
ωB97X	1.03	1.03	0.99	0.99

a All values in eV.

b *N* = 15.

The difference in the excitation energies predicted by the isolated and QM/MM optimized models is rather minuscule, with absolute differences ranging from zero to 0.28 eV (Table S6). The largest disparity is again observed for complex **[Cu**_**2**_**I**_**2**_**(pnme)**_**2**_**]**, which also resulted in the largest geometry difference in the ground state with all but the ωB97X functional. Unsurprisingly, ωB97X is also the only functional with negligible difference in the predicted excitation energy between the two models, while the PBE0, LRC-ωPBEh, B3LYP, and CAM-B3LYP functionals show an absolute difference in the excitation energy of 0.19, 0.25, 0.14, and 0.28 eV, respectively (Table S6). Interestingly, while **[Cu(ccpyr)(ttpp)]** also resulted in a significant ground-state geometry difference between the isolated and QM/MM models, the predicted excitation energies are virtually identical between the two models (Table S6). Closer inspection of these two systems reveals that while the isolated models produce distinctly differing ground-state geometries, only in **[Cu**_**2**_**I**_**2**_**(pnme)**_**2**_**]** does it affect the Cu coordination environment, which ultimately results in the observed excitation energy difference. In **[Cu(ccpyr)(ttpp)]**, even though the ground-state geometries differ quite significantly, the Cu coordination environment is mostly unaffected (Figure S1), thus having virtually no influence on the excitation energy.

Comparing the isolated models in solution against experimental excitation energies, the same relative trend is observed (in increasing MAD): PBE0 < B3LYP < LRC-ωPBEh < CAM-B3LYP ≪ ωB97X. As in the solid state, the hybrid functionals predict slightly underestimated excitation energies, while the range-separated hybrids overestimate the transition energies (Table 3).

**Table 3:** Mean Absolute Deviations (MAD) and Mean Signed Deviations (MSD) against Experimental Data Obtained in Solution for the S_0_ → S_1_ Excitation Energies^a^^b^

	MAD	MSD
PBE0	0.20	–0.16
LRC-ωPBEh	0.50	0.46
B3LYP	0.34	–0.33
CAM-B3LYP	0.57	0.55
ωB97X	0.95	0.95

a All values in eV.

b *N* = 28.

### Excited-State Structures

**Figure 9:**
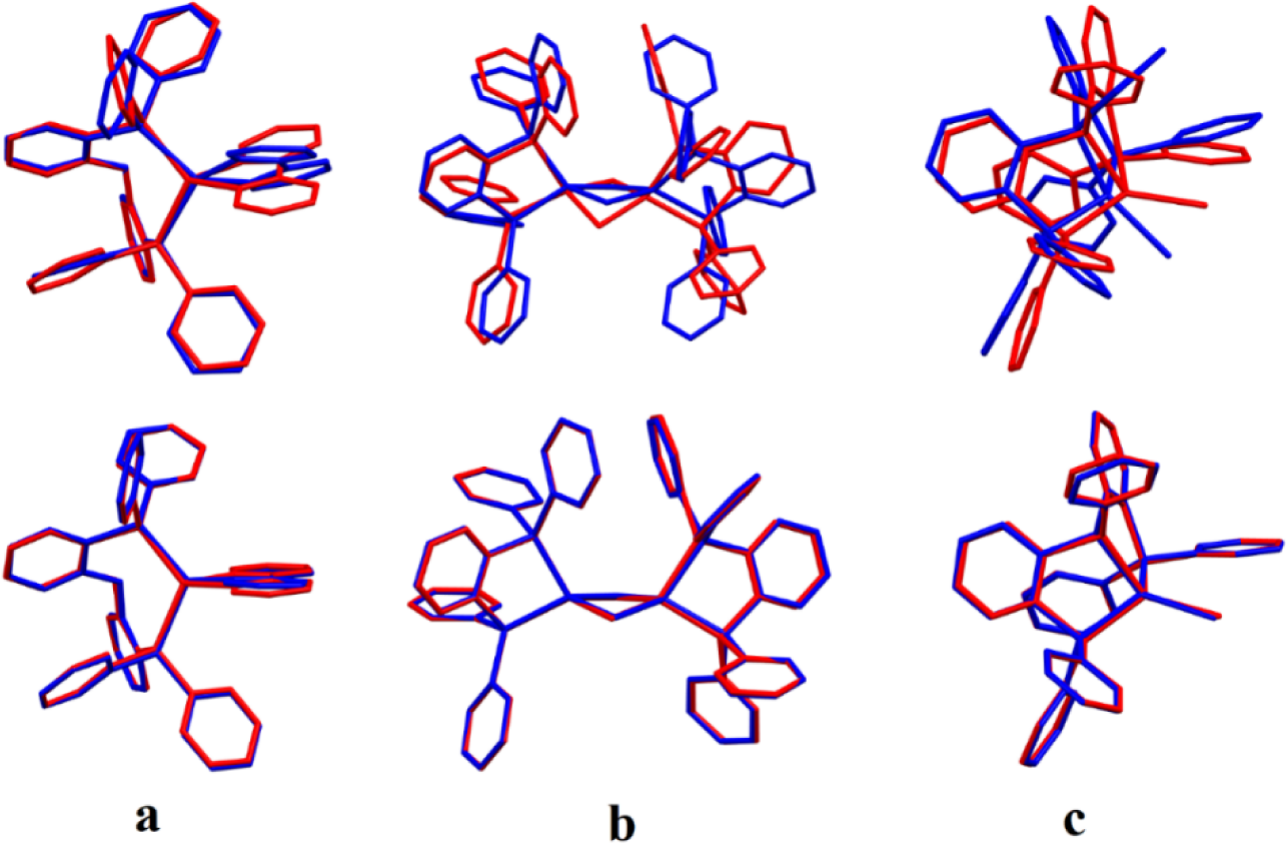
Superimposed structures of LRC-ωPBEh optimized ground (S_0_, red) and excited singlet state (S_1_, blue) for **[Cu(pop)(bpy)]**^**+**^, (a) **[Cu**_**2**_**Cl**_**2**_**(dppb)**_**2**_**]** (b), and **[CuI(ttpp)]** (c). Top row = isolated models; bottom row = QM/MM models. Hydrogens omitted for clarity.

We now turn to the excited states and start by analyzing the geometry relaxation upon excitation between the isolated and QM/MM models. Table 4 shows the average geometry relaxation between ground and excited singlet (S_1_) state structures, measured as the RMSD between the superimposed geometries for both model systems. For the data of each individual complex, see Tables S7–S11. Using the isolated models, significant geometry relaxation is observed in the excited state, with average RMSD values ranging from 0.75 Å to 1.00 Å, depending on the functional used. However, with the QM/MM models, the added rigidity of the MM layer considerably restricts the geometry relaxation, as is evident from the RMSD values, which are reduced to only a fraction of the analogous values for the isolated model (Figure 6b). This effect is also clearly observed by superimposing the ground- and excited-state structures (Figure 9). Moreover, the choice of functional has very limited influence on the geometry relaxation when the QM/MM model is used, with the PBE0, LRC-ωPBEh, B3LYP, CAM-B3LYP, and ωB97X functionals resulting in average RMSDs of 0.15, 0.14, 0.14, 0.15, and 0.16 Å, respectively. The same conclusion also holds for the geometry difference between the ground and excited triplet (T_1_) and the excited singlet (S_1_) and triplet (T_1_) states (Figures 6c,d, Tables S12 and S13).

**Table 4:** Average RMSD (Å) between the Optimized Ground-State (S_0_) and Excited Singlet State (S_1_) Structures^a^

	PBE0	LRC-ωPBEh	B3LYP	CAM-B3LYP	ωB97X
ISOLATED					
[Cu(NN)(PP)]^+^	0.50	0.52	0.56	0.63	0.73
[Cu_2_X_2_(L^2^)_2_]	1.32	1.34	1.38	1.56	0.81
[CuX(L^3^)]	0.81	0.81	0.76	0.79	0.70
**All**	0.88	0.90	0.90	1.00	0.75
QM/MM					
[Cu(NN)(PP)]^+^	0.18	0.16	0.17	0.17	0.17
[Cu_2_X_2_(L^2^)_2_]	0.16	0.14	0.14	0.15	0.17
[CuX(L^3^)]	0.11	0.12	0.11	0.13	0.13
**All**	0.15	0.14	0.14	0.15	0.16

a *N* = 56.

To look beyond the simple RMSD metric and specifically assess the role of Jahn–Teller effect-induced flattening distortions on geometry relaxation, we examined the optimized geometries obtained with the LRC-ωPBEh functional and introduced a flattening angle and flattening degree. The flattening angle is defined as the angle between two planes spanned by the central Cu ion and two coordinating atoms. For the [Cu(NN)(PP)]^+^ group, the two planes are spanned by the N–Cu–N and P–Cu–P planes; for the [Cu_2_X_2_(L^2^)_2_] group, by X-Cu-X and L-Cu-L planes; and for the [CuX(L^3^)] group, by X–Cu–(**L**^L^L) and (L^**L**^L)–Cu–(L^L^**L**) planes (where the atom written in bold defines the specific coordinating atom within the tridentate ligand) (Figure S2). In an ideal tetrahedral geometry, the planes would be perpendicular to one another, whereas in an ideal square-planar geometry, they would be parallel to one another. The flattening degree is defined as the difference in the flattening angles between the ground (S_0_)- and excited (S_1_)-state geometries, thus measuring the change from a (pseudo)tetrahedral geometry toward a square-planar one. Using the isolated models, the flattening degree varies quite drastically, ranging from zero up to a maximum value of 76.5° (although we note that such extreme values were only observed for two complexes: **[Cu**_**2**_**I**_**2**_**(phen)**_**2**_**]** and **[Cu**_**2**_**Cl**_**2**_**(dpmt)**_**2**_**]**) and an average (median) of 22.4° (24.1°). Excluding the two outliers results in a maximum flattening angle of 38.4°. With the QM/MM models, however, the maximum flattening degree is reduced to 21.0° and an average (median) of 5.8° (5.3°) (Table 5). Although both models experience flattening distortions caused by the Jahn–Teller effect, the extent of geometry relaxation is significantly reduced in the QM/MM models due to the added rigidity of the model. Worth noting is the group of tripodal [CuX(tpyYZ)] complexes, all of which show less than a 4° difference in the flattening degree between the isolated and QM/MM models (Table S14). Because of the very unyielding coordination environment, these systems are able to resist flattening distortions to a large extent.

**Table 5:** Average, Median, and Maximum Flattening Degrees (°) for the Isolated and QM/MM Models with the LRC-ωPBEh Functional^a^

	ISOLATED			QM/MM		
	Avg	Mdn	Max	Avg	Mdn	Max
[Cu(NN)(PP)]^+^	22.6	22.5	32.4	12.0	11.8	21.0
[Cu_2_X_2_(L^2^)_2_]	27.9	28.1	76.5	2.6	1.5	8.8
[CuX(L^3^)]	16.8	21.0	38.4	3.2	3.1	8.5
**All**	**22.4**	**24.1**	**76.5**	**5.8**	**5.3**	**21.0**

a *N* = 56.

### Fluorescence Energies and Nature of the Excited States

Turning to the predicted fluorescence energies, both the PBE0 and B3LYP functionals substantially underestimate the transition energies when using the geometries optimized with the isolated model, resulting in MADs (MSDs) of 0.72 (−0.72) eV and 0.81 (−0.81) eV, respectively (Tables 6 and S15). However, when the QM/MM-optimized geometries are used, these errors are reduced to 0.37 (−0.36) eV and 0.49 (−0.49) eV, respectively (Tables 6 and S16). This is because the flattening distortions induce a stabilizing effect on the excited-state potential energy surface, leading to red-shifted emission the more the excited-state geometry is relaxed (Figure S3). The range-separated hybrid functional LRC-ωPBEh yields the best estimates for the fluorescence energies with respect to experimental measurements, with an MAD and MSD of 0.30 eV and −0.25 eV when the isolated model is used. Again, usage of the QM/MM model blue-shifts the predicted transition energies, resulting in reduced MAD and MSD values of only 0.20 and 0.12 eV, which are rather impressive results for TD-DFT. Interestingly, for the CAM-B3LYP functional, the deviations with respect to experiment are almost identical (albeit with a reversed sign) between the two models, with the isolated model slightly underestimating the fluorescence energies, whereas the QM/MM model tends to overestimate them, as indicated by the MSDs of −0.18 and 0.19 eV. The ωB97X functional presents a case where the mean absolute and signed deviations are considerably lower when using the geometries obtained with the isolated model (0.26 and 0.22 eV vs 0.50 and 0.50 eV, respectively). Naively, one might interpret this as an endorsement of the isolated model’s superiority over the QM/MM model. Therefore, let us remind ourselves of the results obtained earlier in the excitation energies section. With either model, the ωB97X functional overestimated the excitation energies by roughly 1 eV (since the flattening distortions only manifest in the excited state, and thus, the differences in ground-state geometries were mostly negligible). Combining the ωB97X functional’s tendency to overshoot the transition energies and the isolated model’s tendency to produce overly flattened geometries (which induce a red-shift in transition energy) allows one to conclude that the ωB97X functional massively benefits from error cancellation when used in combination with the isolated models.

**Table 6:** Mean Absolute and Signed Deviations (eV) with Respect to the Experiment for the Fluorescence Energies with the Isolated and QM/MM Models^a^

	ISOLATED		QM/MM	
	MAD	MSD	MAD	MSD
PBE0	0.72	–0.72	0.37	–0.36
LRC-ωPBEh	0.30	–0.25	0.20	0.12
B3LYP	0.81	–0.81	0.49	–0.49
CAM-B3LYP	0.24	–0.18	0.23	0.19
ωB97X	0.26	0.22	0.50	0.50

a *N* = 56.

**Figure 10:**
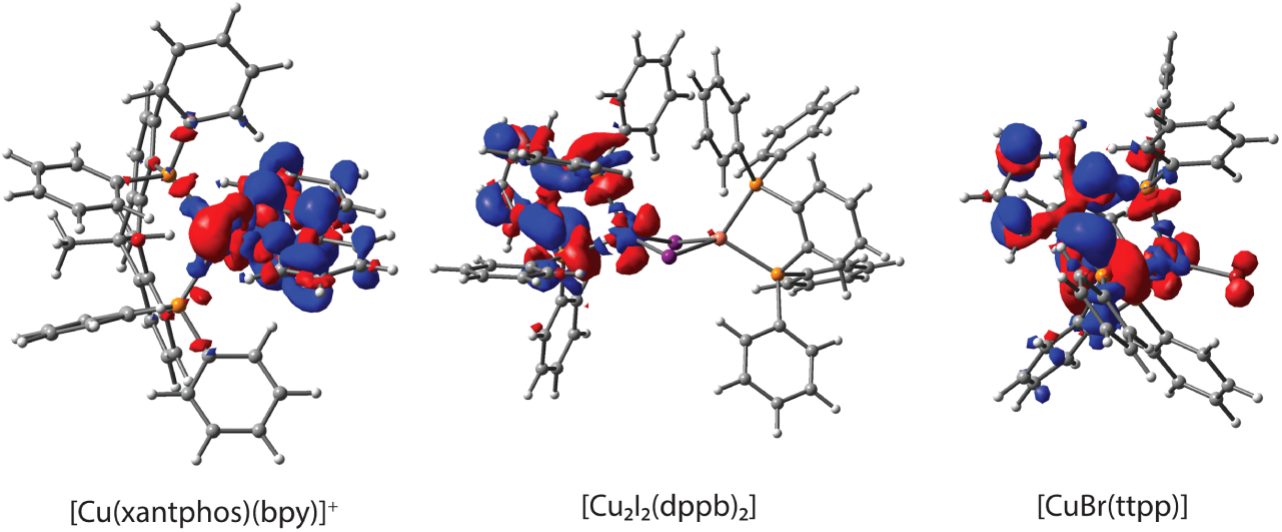
Electron density difference plots for S_1_ → S_0_ fluorescence emission on a 0.002 au isosurface for **[Cu(xantphos)(bpy)]**^**+**^, **[Cu**_**2**_**I**_**2**_**(dppb)**_**2**_**]**, and **[CuBr(ttpp)]** with the LRC-ωPBEh functional and QM/MM model. Red (blue) regions imply an increase (decrease) of electron density during the transition.

For characterizing the emitting singlet states, we use electron density difference plots (Figures 10 and S4–S18). All complexes in the [Cu(NN)(PP)]^+^ group can be characterized as ^1^MLCT emitters, with the electron density shifting between the copper ion and the NN-donor ligand, along with a small admixture of ligand-centered (LC) π-π* character (Figures S4–S8). Likewise, ^1^MLCT fluorescence from a d → π* state is predicted for the [Cu_2_X_2_(L^2^)_2_] group as well (Figures S9–S13). For the [CuX(L^3^)] group, an additional (pseudo)halide → π* component is predicted, resulting in a ^1^(M + X)LCT emission (Figures S14–S18). All tested functionals predict the same emitting states, although the functionals without long-range corrections (PBE0 and B3LYP) predict a visibly higher contribution from the (pseudo)halides in complexes of ^1^(M + X)LCT emission (Figures S4–S18).

### Singlet–Triplet Energy Differences

Lastly, we assess the predicted Δ*E*(S_1_ – T_1_) differences, as this is the most important parameter for achieving TADF emission. All energy differences are calculated as total energy differences in their optimized geometries. Since experimentally determined Δ*E*(S_1_ – T_1_) values were available for only a handful of the complexes included in this study, we use a simple pass/fail evaluation criterion. In order to pass, the predicted Δ*E*(S_1_ – T_1_) must be positive and below 0.37 eV. Although there is no clear-cut line regarding the maximum allowed Δ*E*(S_1_ – T_1_) while maintaining reasonably efficient reverse intersystem crossing rates, since the efficiency of rISC depends on the amount of SOC and/or (spin-)vibronic coupling as well, the literature consensus seems to fall somewhere between 1500 cm^–1^ and 3000 cm^–1^.^27,87,88^ We have therefore selected the more forgiving threshold of 3000 cm^–1^ (0.37 eV). Despite the fact that negative singlet–triplet energy differences are well documented in the literature in the context of so-called inverted singlet–triplet emitters, they require a distinct double excitation character, which TD-DFT in its adiabatic approximation formalism is unable to describe.^89,90^ Therefore, negative singlet–triplet gaps are classified here as unphysical. Negative singlet–triplet gaps likely arise from an imbalanced description of singlet and triplet states, resulting in the singlet states being relaxed to a greater extent than the triplet states. Note that if one were to estimate the Δ*E*(S_1_ – T_1_) as differences in vertical excitation energies from the ground state (or any other state for that matter), the issue of negative singlet–triplet gaps would be resolved (Table S17). Negative singlet–triplet gaps are mainly predicted when the isolated models are utilized, especially in combination with the B3LYP functional, with 18 of the 25 entries in this category being associated with the B3LYP functional (Tables 7, S18 and S19). Usage of QM/MM models mostly eliminates the negative singlet–triplet gaps, resulting in only 2 systems failing to meet this condition (both with the B3LYP functional). When it comes to the predicted Δ*E*(S_1_ – T_1_) values being higher than the 0.37 eV threshold, the hybrid functionals tend to do slightly better than the range-separated hybrids with 0 (1) and 0 (1) of the systems failing to meet this condition with the isolated (QM/MM) models using PBE0 or B3LYP functional, respectively. With their range-separated counterparts, LRC-ωPBEh and CAM-B3LYP, 4 (4), and 3 (6) systems show singlet–triplet gaps higher than 0.37 eV. The ωB97X functional struggles by far the most, with 11 (9) systems failing to meet the Δ*E*(S_1_ – T_1_) < 0.37 eV condition using isolated (QM/MM) models.

**Table 7:** Number of Systems Failing to Meet the 0 < Δ*E*(S_1_ – T_1_) < 0.37 eV Criteria^a^

	PBE0	LRC-ωPBEh	B3LYP	CAM-B3LYP	ωB97X
ISOLATED					
Negative	3	2	18	1	1
Too high	0	4	0	3	11
QM/MM					
Negative	0	0	2	0	0
Too high	1	4	1	6	9

a *N* = 56.

## Conclusions

We compared the results obtained using isolated and QM/MM models with commonly used hybrid and range-separated hybrid density functionals on a set of 56 experimentally characterized tetrahedral Cu(I) TADF emitters. Special emphasis between the isolated and QM/MM models is placed on the difference in predicted geometries and its effect on excitation, fluorescence, and relative energies. Regarding the ground-state structures, the QM/MM models consistently provide geometries closer to the experimental XRD reference data. That said, the differences when using the isolated models mainly arise from freely rotating groups (such as phenyl groups) and/or geometry relaxation due to steric repulsion. On the other hand, the choice of a density functional method had very little influence on the predicted ground-state geometries, especially when the QM/MM models were used. The only noteworthy difference was the ωB97X functional’s ability to resist steric repulsion-induced geometry relaxation when used together with the isolated model, which is likely caused by a better description of noncovalent interactions.

Despite the differences in ground-state geometries between the two models, the differences in the predicted excitation energies were mostly negligible. Only when the change in geometry affected the coordination geometry of the central copper ion was there a noticeable difference in excitation energies. The usage of the hybrid PBE0 functional resulted in the smallest MAD with respect to experimental results, followed closely by the B3LYP hybrid and range-separated hybrid LRC-ωPBEh and CAM-B3LYP functionals. The range-separated functional ωB97X resulted in by far the highest MAD of roughly 1 eV. In general, the hybrid functionals PBE0 and B3LYP underestimated the excitation energies, whereas the range-separated hybrids LRC-ωPBEh, CAM-B3LYP, and ωB97X predicted overestimated excitation energies compared to the experiment. This is not surprising given the problems associated with CT transition energies in TD-DFT when functionals with no or low amounts of Hartree–Fock exchange are used and, conversely, the range-separated functionals’ tendency to stabilize states with CT character.

For the excited-state geometries, even more structural dissimilarities were observed between the isolated and the QM/MM models. More importantly, the isolated models produced geometries that were flattened to a significantly larger extent by the Jahn–Teller effect, whereas the added rigidity of the QM/MM models restricted the degree of geometry relaxation, thereby mimicking the solid-state environment. As with ground-state geometries, when the QM/MM models were utilized, differences between the functionals were negligible.

As expected, for predicted fluorescence energies, the use of QM/MM models resulted in blue-shifted transition energies with respect to the isolated models. This is because the flattening distortions induce a stabilizing effect on the excited-state potential energy surface, resulting in red-shifted transition energy the more the geometry is able to relax. With the PBE0, B3LYP, and LRC-ωPBEh functionals, the use of QM/MM models significantly decreased the MAD and MSD with respect to experimental data, with LRC-ωPBEh predicting fluorescence energies closest to the experiment, achieving an MAD and MSD of 0.20 and 0.12 eV, respectively. On the other hand, ωB97X benefited greatly from error cancellation when used together with the isolated models, resulting in a lower MAD than with the QM/MM models. All functionals predicted the same nature for the emitting singlet state, although the (M + X)LCT states encountered for the [CuX(L^3^)] group were slightly more delocalized with the hybrid functionals PBE0 and B3LYP than with the range-separated hybrid functionals.

For the singlet–triplet energy gaps, Δ*E*(S_1_ – T_1_), use of QM/MM models mostly eliminated the negative energy gaps, presumably caused by the imbalanced description of singlet and triplet states. PBE0 and B3LYP predicted the fewest systems where Δ*E*(S_1_ – T_1_) was too high for efficient reverse intersystem crossing to take place, followed closely by their range-separated counterparts, LRC-ωPBEh and CAM-B3LYP. Out of the tested functionals, ωB97X showed the worst performance in this category and predicted by far the largest number of systems with excessively high singlet–triplet energy gaps.

Although an in-depth understanding of photophysics and excited-state dyanamics in TADF emitters would require a thorough investigation of the relevant radiative and nonradiative rates, the results obtained in this study highlight the importance of the chosen model system. The usage of simple isolated single-molecule models to study molecules in their solid state may lead to unrealistic geometries, which can have a cascading adverse effect on subsequent property calculations. We hope the findings presented here provide useful insight into improving the predictive power of theoretical studies in designing novel TADF emitter materials.

## Data Availability

In addition, all optimized geometries (.xyz), electron density difference files (.cube), and example input files (.inp) are available at DOI: 10.5281/zenodo.14045652

## Supporting Information Available

Photophysical properties, RMSD values, excitation and fluorescence energies, additional details for flattening angles and degrees, electron density difference plots, and singlet–triplet energy differences (PDF)
