# Supplementary material for: Tetrahedral Cu(I) Complexes for Thermally Activated Delayed Fluorescence: A Density Functional Benchmark Study with QM/MM Models
Source: Inorg Chem. 2025 Apr 30;64(18):9150–62. doi: 10.1021/acs.inorgchem.5c00761 (PMC12076558; doi:10.1021/acs.inorgchem.5c00761)
Supplement: Supplementary file 1 — ic5c00761_si_001.pdf [file ic5c00761_si_001.pdf]

Tetrahedral Cu(I) Complexes for Thermally Activated Delayed Fluorescence: A Density Functional Benchmark Study with QM/MM Models

Supporting Information

Toni Eskelinen [a*], Antti J. Karttunen [a]

[a] *Department of Chemistry and Materials Science, School of Chemical Engineering, Aalto University, Kemistintie 1, 02150 Espoo, Finland.*

* Email: toni.eskelinen@aalto.fi

Table S1. Photophysical properties of the studied emitters.

	298 K		77 K		
	λ, em [nm]	QY	λ, em [nm]	$\Delta E(S_1\text{-}T_1)$ [eV]	ref.
**[Cu(pop)(pypz)]⁺**	490	0.56	508	0.18	1
**[Cu(pop)(pympz)]⁺**	465	0.87	493	0.17	1
**[Cu(pop)(pytfmpz)]⁺**	492	0.75	511	0.18	1
**[Cu(pop)(bpy)]⁺**	581	0.03	610	N/A	2, 3
**[Cu(pop)(mbpy)]⁺**	567	0.10	N/A	N/A	4
**[Cu(pop)(dmbpy)]⁺**	535	0.43	N/A	N/A	4, 5
**[Cu(pop)(etbpy)]⁺**	557	0.24	N/A	N/A	6
**[Cu(pop)(phbpy)]⁺**	576	0.05	N/A	N/A	6
**[Cu(pop)(tfmbpy)]⁺**	575	0.06	610	N/A	2
**[Cu(pop)(55dtfmbpy)]⁺**	648	0.01	656	N/A	2
**[Cu(pop)(44dtfmbpy)]⁺**	664	0.01	650	N/A	2
**[Cu(xanthpos)(tfmbpy)]⁺**	581	0.11	595	N/A	2
**[Cu(xanthpos)(phbpy)]⁺**	563	0.04	N/A	N/A	6
**[Cu(xanthpos)(dmbpy)]⁺**	539	0.37	551	N/A	2, 7
**[Cu(xanthpos)(44dtfmbpy)]⁺**	632	0.01	652	N/A	2
**[Cu(xanthpos)(bpy)]⁺**	587	0.02	613	N/A	2, 7
**[Cu(xanthpos)(etbpy)]⁺**	545	0.37	N/A	N/A	6
**[Cu(xanthpos)(mbpy)]⁺**	547	0.34	567	N/A	2, 7
**$[Cu_2Cl_2(dpmb)_2]$**	527	0.29	532	N/A	8
**$[Cu_2Cl_2(dpmt)_2]$**	543	0.35	547	N/A	9
**$[Cu_2Cl_2(dppb)_2]$**	533	0.60	537	N/A	10
**$[Cu_2Cl_2(pnme)_2]$**	506	0.45	513	0.06	11
**$[Cu_2Br_2(dpmb)_2]$**	511	0.28	517	N/A	8
**$[Cu_2Br_2(dpmt)_2]$**	542	0.26	546	N/A	9
**$[Cu_2Br_2(dppb)_2]$**	520	0.60	524	N/A	10
**$[Cu_2Br_2(pnme)_2]$**	490	0.65	498	0.06	11
**$[Cu_2I_2(dpmb)_2]$**	498	0.32	500	N/A	8
**$[Cu_2I_2(dpmt)_2]$**	490	0.32	496	N/A	9
**$[Cu_2I_2(dppb)_2]$**	502	0.80	505	N/A	10
**$[Cu_2I_2(pnme)_2]$**	464	0.65	471	0.07	11
**$[Cu_2I_2(pnpy)_2]$**	465	0.65	465	0.08	11
**$[Cu_2I_2(phen)_2]$**	688	0.01	N/A	N/A	12
**$[Cu_2I_2(dmp)_2]$**	667	0.18	660, 720	N/A	12
**$[Cu_2I_2(dppt1)_2]$**	487	0.69	500	0.07	13
**$[Cu_2I_2(dppt2)_2]$**	483	0.86	494	0.05	13
**$[Cu_2(SCN)_2(dppb)_2]$**	571	0.14	571	N/A	14

**[$Cu_2(SCN)_2(pop)_2$]**	448	0.15	452	N/A	14
**[CuBr(dmpzpp)]**	541	0.83	551	N/A	15
**[CuBr(tpypo)]**	620	0.18	620	N/A	16
**[CuBr(ttpp)]**	523	0.79	531	0.04	17
**[CuCl(tpypo)]**	645	0.08	645	N/A	16
**[CuCl(ttpp)]**	530	0.76	531	0.01	17
**[CuI(dmpzpp)]**	530	0.82	530	N/A	15
**[CuI(tpyaso)]**	600	0.12	610	N/A	16
**[CuI(tpym)]**	550	0.28	550	N/A	16
**[CuI(tpypo)]**	600	0.20	610	N/A	16
**[CuI(tpyps)]**	595	0.34	595	N/A	16
**[CuI(tpypse)]**	640	0.04	675	N/A	16
**[CuI(ttpp)]**	521	0.83	531	0.05	17
**[CuI(ttppo)]**	621	0.01	624	N/A	18
**[Cu(CN)(ttpp)]**	530	0.70	N/A	N/A	19
**[Cu(SCN)(ttpp)]**	520	0.57	520	0.08	14
**[Cu(SPh)(dmpzpp)]**	540	0.90	540	N/A	15
**[Cu(ccph)(ttpp)]**	602	0.06	612	N/A	20
**[Cu(ccnph)(ttpp)]**	722	<0.01	717	N/A	20
**[Cu(ccpyr)(ttpp)]**	573	0.19	579	N/A	20

Table S2. RMSD (Å) between XRD and optimized ground state structures using the isolated models.

	ISOLATED				
	PBE0	LRC-ωPBEh	B3LYP	CAM-B3LYP	ωB97X
cu_pop_pypz	0.38	0.38	0.42	0.38	0.39
cu_pop_pympz	0.50	0.52	0.52	0.52	0.63
cu_pop_pytfmpz	0.84	0.55	0.76	0.73	0.76
cu_pop_bpy	0.43	0.52	0.41	0.49	0.66
cu_pop_mbpy	0.58	0.57	0.58	0.56	0.49
cu_pop_dmbpy	0.29	0.31	0.33	0.33	0.38
cu_pop_etbpy	0.52	0.53	0.73	0.74	0.77
cu_pop_phbpy	0.54	0.57	0.56	0.55	0.50
cu_pop_tfmbpy	0.38	0.42	0.51	0.48	0.27
cu_pop_55dtfmbpy	0.65	0.48	0.41	0.48	0.64
cu_pop_44dtfmbpy	0.54	0.45	0.55	0.51	0.93
cu_xantphos_tfmbpy	0.45	0.62	0.46	0.40	0.31
cu_xantphos_phbpy	0.31	0.33	0.36	0.32	0.48
cu_xantphos_dmbpy	0.74	0.73	0.66	0.64	0.74
cu_xantphos_44dtfmbpy	0.37	0.38	0.42	0.37	0.34
cu_xantphos_bpy	0.70	0.70	0.74	0.70	0.71
cu_xantphos_etbpy	0.42	0.42	0.50	0.44	0.55
cu_xantphos_mbpy	0.39	0.39	0.47	0.43	0.55
cu2_cl2_dpmb2	1.05	1.04	1.07	1.07	0.95
cu2_cl2_dpmt2	0.74	0.87	1.05	1.06	0.78
cu2_cl2_dppb2	0.55	0.55	1.02	0.86	0.49
cu2_cl2_pnme22	0.58	0.84	0.62	0.59	0.56
cu2_br2_dpmb2	0.71	0.73	1.02	0.85	0.95
cu2_br2_dpmt2	0.75	0.82	1.00	0.94	1.01
cu2_br2_dppb2	0.68	0.57	0.96	0.74	0.58
cu2_br2_pnme22	1.59	1.59	1.82	1.76	0.44
cu2_i2_dpmb2	0.71	0.70	0.97	0.77	1.06
cu2_i2_dpmt2	0.54	0.58	0.63	0.56	0.61

cu2_i2_dppb2	0.53	0.45	0.99	0.47	0.52
cu2_i2_pnme22	1.61	1.64	1.61	1.58	0.66
cu2_i2_pnpy22	1.45	1.49	1.88	1.55	0.81
cu2_i2_phen2	0.84	0.87	0.77	0.87	0.71
cu2_i2_dmp2	0.40	0.41	0.51	0.42	0.47
cu2_i2_dppt12	0.62	0.63	0.30	1.11	0.62
cu2_i2_dppt22	0.76	0.75	1.42	0.76	0.70
cu2_scn2_dppb2	1.30	1.28	1.28	1.26	1.65
cu2_scn2_pop2	0.94	0.90	0.61	0.92	0.58
cu_br_dmpzpp	0.43	0.42	0.49	0.45	0.44
cu_br_tpypo	0.13	0.13	0.15	0.15	0.13
cu_br_ttpp	0.64	0.75	0.58	0.67	0.50
cu_cl_tpypo	0.12	0.11	0.12	0.11	0.11
cu_cl_ttpp	0.63	0.72	0.61	0.67	0.52
cu_i_dmpzpp	0.24	0.24	0.35	0.27	0.32
cu_i_tpyaso	0.14	0.14	0.15	0.14	0.14
cu_i_tpym	0.15	0.15	0.15	0.15	0.15
cu_i_tpypo	0.13	0.13	0.14	0.13	0.13
cu_i_tpyps	0.30	0.29	0.30	0.29	0.29
cu_i_tpypse	0.17	0.16	0.18	0.16	0.16
cu_i_ttpp	0.60	0.55	0.58	0.66	0.50
cu_i_ttppo	0.61	0.48	0.60	0.51	0.63
cu_cn_ttpp	0.59	0.59	0.60	0.57	0.60
cu_scn_ttpp	0.49	0.50	0.59	0.61	0.42
cu_sph_dmpzpp	0.33	0.38	0.46	0.33	0.36
cu_ccph_ttpp	0.88	0.89	0.85	0.91	1.04
cu_ccnph_ttpp	0.63	0.64	0.62	0.65	0.77
cu_ccpyr_ttpp	0.91	0.93	0.92	0.97	1.14

Table S3. RMSD (Å) between XRD and optimized ground state structures using the QM/MM models.

	QM/MM				
	PBE0	LRC-ωPBEh	B3LYP	CAM-B3LYP	ωB97X
cu_pop_pypz	0.21	0.21	0.24	0.21	0.21
cu_pop_pympz	0.21	0.21	0.23	0.21	0.23
cu_pop_pytfmpz	0.28	0.28	0.30	0.29	0.29
cu_pop_bpy	0.20	0.20	0.22	0.21	0.21
cu_pop_mbpy	0.20	0.20	0.22	0.22	0.21
cu_pop_dmbpy	0.17	0.17	0.19	0.18	0.18
cu_pop_etbpy	0.16	0.15	0.20	0.16	0.13
cu_pop_phbpy	0.16	0.15	0.18	0.16	0.14
cu_pop_tfmbpy	0.16	0.14	0.19	0.15	0.13
cu_pop_55dtfmbpy	0.17	0.17	0.19	0.17	0.17
cu_pop_44dtfmbpy	0.16	0.16	0.19	0.17	0.16
cu_xantphos_tfmbpy	0.19	0.19	0.22	0.18	0.17
cu_xantphos_phbpy	0.18	0.18	0.19	0.18	0.18
cu_xantphos_dmbpy	0.20	0.21	0.22	0.20	0.20
cu_xantphos_44dtfmbpy	0.19	0.19	0.20	0.19	0.19
cu_xantphos_bpy	0.16	0.16	0.18	0.16	0.16
cu_xantphos_etbpy	0.18	0.17	0.19	0.17	0.16
cu_xantphos_mbpy	0.16	0.16	0.20	0.17	0.15
cu2_cl2_dpmb2	0.17	0.17	0.19	0.17	0.15

cu2_cl2_dpmt2	0.30	0.31	0.34	0.31	0.26
cu2_cl2_dppb2	0.17	0.17	0.20	0.17	0.15
cu2_cl2_pnme22	0.16	0.15	0.18	0.16	0.16
cu2_br2_dpmb2	0.30	0.30	0.31	0.30	0.27
cu2_br2_dpmt2	0.31	0.33	0.40	0.39	0.35
cu2_br2_dppb2	0.16	0.16	0.18	0.17	0.15
cu2_br2_pnme22	0.19	0.18	0.21	0.19	0.19
cu2_i2_dpmb2	0.24	0.23	0.25	0.23	0.23
cu2_i2_dpmt2	0.24	0.24	0.27	0.24	0.21
cu2_i2_dppb2	0.20	0.19	0.23	0.22	0.17
cu2_i2_pnme22	0.22	0.22	0.31	0.27	0.25
cu2_i2_pnpy22	0.17	0.16	0.21	0.17	0.15
cu2_i2_phen2	0.14	0.14	0.17	0.15	0.16
cu2_i2_dmp2	0.29	0.30	0.32	0.30	0.29
cu2_i2_dppt12	0.27	0.27	0.29	0.27	0.25
cu2_i2_dppt22	0.18	0.16	0.38	0.34	0.17
cu2_scn2_dppb2	0.18	0.18	0.21	0.19	0.19
cu2_scn2_pop2	0.17	0.16	0.21	0.18	0.14
cu_br_dmpzpp	0.18	0.17	0.19	0.18	0.20
cu_br_tpypo	0.12	0.12	0.14	0.14	0.14
cu_br_ttpp	0.13	0.13	0.15	0.13	0.14
cu_cl_tpypo	0.19	0.19	0.21	0.19	0.20
cu_cl_ttpp	0.14	0.14	0.15	0.14	0.15
cu_i_dmpzpp	0.20	0.19	0.23	0.25	0.24
cu_i_tpyaso	0.15	0.15	0.16	0.15	0.15
cu_i_tpym	0.11	0.11	0.12	0.11	0.11
cu_i_tpypo	0.15	0.15	0.16	0.15	0.15
cu_i_tpyps	0.12	0.12	0.13	0.12	0.12
cu_i_tpypse	0.12	0.12	0.12	0.12	0.12
cu_i_ttpp	0.14	0.14	0.15	0.14	0.14
cu_i_ttppo	0.14	0.13	0.16	0.14	0.14
cu_cn_ttpp	0.12	0.12	0.13	0.12	0.12
cu_scn_ttpp	0.20	0.19	0.21	0.19	0.18
cu_sph_dmpzpp	0.20	0.20	0.24	0.21	0.23
cu_ccph_ttpp	0.21	0.21	0.31	0.30	0.22
cu_ccnph_ttpp	0.12	0.11	0.14	0.12	0.12
cu_ccpyr_ttpp	0.15	0.15	0.19	0.18	0.19

Table S4. $S_0 \rightarrow S_1$ excitation energies and errors with respect to experiment for the isolated models. Entries written in italics are obtained in solution.

	ISOLATED									
	PBE0		LRC-ωPBEh		B3LYP		CAM-B3LYP		ωB97X	
	E [eV]	Error [eV]	E [eV]	Error [eV]	E [eV]	Error [eV]	E [eV]	Error [eV]	E [eV]	Error [eV]
cu_pop_pypz[a]	*3.47*	*-0.10*	*4.15*	*0.58*	*3.28*	*-0.30*	*4.23*	*0.66*	*4.71*	*1.13*
cu_pop_pympz[a]	*3.53*	*-0.05*	*4.27*	*0.68*	*3.33*	*-0.25*	*4.33*	*0.74*	*4.72*	*1.14*
cu_pop_pytfmpz[a]	*3.29*	*-0.21*	*4.04*	*0.55*	*3.07*	*-0.42*	*4.24*	*0.75*	*4.56*	*1.07*
cu_pop_bpy[a]	*3.06*	*-0.14*	*3.62*	*0.43*	*2.89*	*-0.30*	*3.78*	*0.58*	*4.21*	*1.01*
cu_pop_mbpy[a]	*3.04*	*-0.22*	*3.63*	*0.37*	*2.84*	*-0.42*	*3.72*	*0.45*	*4.15*	*0.89*
cu_pop_dmbpy[a]	*3.12*	*-0.21*	*3.77*	*0.44*	*2.96*	*-0.37*	*3.85*	*0.52*	*4.33*	*0.99*
cu_pop_etbpy[a]	*3.07*	*-0.11*	*3.71*	*0.53*	*2.88*	*-0.30*	*3.78*	*0.60*	*4.20*	*1.02*
cu_pop_phbpy[a]	*3.04*	*-0.06*	*3.64*	*0.54*	*2.87*	*-0.23*	*3.73*	*0.63*	*4.21*	*1.11*

cu_pop_tfmbpy[a]	2.98	-0.29	3.59	0.33	2.79	-0.47	3.74	0.47	4.20	0.94
cu_pop_55dtfmbpy[a]	2.62	-0.19	3.32	0.51	2.48	-0.34	3.39	0.57	3.87	1.06
cu_pop_44dtfmbpy[a]	2.73	-0.15	3.34	0.45	2.55	-0.33	3.44	0.56	3.92	1.04
cu_xantphos_tfmbpy[a]	2.94	-0.32	3.55	0.29	2.73	-0.53	3.64	0.38	4.12	0.85
cu_xantphos_phbpy[a]	3.10	-0.08	3.73	0.55	2.93	-0.25	3.83	0.65	4.28	1.10
cu_xantphos_dmbpy[a]	3.15	-0.13	3.75	0.48	2.83	-0.44	3.74	0.47	4.25	0.97
cu_xantphos_44dtfmbpy[a]	2.82	-0.06	3.40	0.51	2.57	-0.32	3.41	0.53	3.87	0.99
cu_xantphos_bpy[a]	3.05	-0.13	3.60	0.42	2.84	-0.34	3.70	0.52	4.16	0.98
cu_xantphos_etbpy[a]	3.10	-0.08	3.63	0.45	2.87	-0.31	3.73	0.55	4.16	0.98
cu_xantphos_mbpy[a]	3.09	-0.18	3.63	0.36	2.87	-0.41	3.73	0.46	4.17	0.90
cu2_cl2_dpmb2[a]	3.37	0.02	3.97	0.61	3.24	-0.12	4.04	0.69	4.30	0.95
cu2_cl2_dpmt2[a]	3.23	-0.21	3.82	0.37	3.08	-0.36	3.88	0.43	4.18	0.74
cu2_br2_dpmb2[a]	3.39	0.04	3.97	0.62	3.27	-0.08	4.08	0.73	4.33	0.98
cu2_br2_dpmt2[a]	3.27	0.17	3.88	0.78	3.12	0.02	3.92	0.82	4.23	1.13
cu2_i2_dpmb2[a]	3.42	0.06	4.06	0.71	3.30	-0.05	4.14	0.79	4.39	1.04
cu2_i2_dpmt2[a]	3.30	0.20	3.92	0.82	3.14	0.04	3.95	0.85	4.27	1.17
cu2_i2_dppb2[b]	3.31	0.05	3.99	0.72	3.24	-0.03	4.05	0.79	4.35	1.08
cu2_i2_pnme22	3.42	0.24	4.17	0.99	3.28	0.10	4.28	1.10	4.38	1.20
cu2_i2_phen2	1.69	-0.67	2.55	0.19	1.52	-0.84	2.62	0.26	3.21	0.85
cu2_i2_dmp2	2.16	-0.09	2.62	0.37	1.76	-0.49	2.89	0.64	3.30	1.05
cu2_i2_dppt12	3.06	-0.70	3.83	0.07	2.85	-0.91	3.91	0.15	4.24	0.48
cu2_i2_dppt22	3.10	-0.55	3.86	0.21	2.91	-0.74	3.92	0.27	4.32	0.67
cu2_scn2_dppb2	3.09	0.10	3.81	0.82	2.91	-0.08	3.86	0.87	4.25	1.26
cu2_scn2_pop2	3.45	0.19	4.30	1.04	3.22	-0.04	4.33	1.07	4.77	1.51
cu_br_ttpp	2.84	-0.27	3.51	0.40	2.67	-0.44	3.58	0.47	3.96	0.84
cu_cl_ttpp	2.88	-0.24	3.51	0.40	2.73	-0.39	3.58	0.47	3.95	0.83
cu_i_tpypo[a]	2.57	-0.22	3.15	0.36	2.38	-0.41	3.31	0.53	3.77	0.99
cu_i_ttpp	2.73	-0.39	3.53	0.41	2.52	-0.60	3.57	0.45	3.97	0.85
cu_i_ttppo	2.50	-0.65	3.60	0.45	2.23	-0.91	3.65	0.51	4.11	0.96
cu_cn_ttpp[a]	3.37	-0.76	4.00	-0.14	3.22	-0.91	4.04	-0.09	4.39	0.26
cu_scn_ttpp	2.70	-0.32	3.57	0.54	2.49	-0.53	3.60	0.58	4.00	0.97
cu_sph_dmpzpp[a]	3.05	-1.14	3.83	-0.36	3.04	-1.15	3.99	-0.20	4.34	0.15
cu_ccph_ttpp	2.90	-0.09	3.58	0.59	2.73	-0.26	3.60	0.61	4.02	1.03
cu_ccnph_ttpp	2.93	0.80	3.60	1.47	2.70	0.57	3.65	1.51	4.03	1.89
cu_ccpyr_ttpp	2.87	-0.12	3.58	0.59	2.71	-0.28	3.61	0.62	4.00	1.01

[a] in dichloromethane, [b] in tetrahydrofuran.

Table S5. $S_0 \rightarrow S_1$ excitation energies and errors with respect to experiment for the QM/MM models.

	QM/MM									
	PBE0		LRC-ωPBEh		B3LYP		CAM-B3LYP		ωB97X	
	E [eV]	Error [eV]	E [eV]	Error [eV]	E [eV]	Error [eV]	E [eV]	Error [eV]	E [eV]	Error [eV]
cu2_i2_pnme22	3.23	0.05	3.92	0.74	3.14	-0.04	4.01	0.83	4.40	1.22
cu2_i2_phen2	1.65	-0.71	2.38	0.02	1.48	-0.88	2.47	0.11	3.04	0.68
cu2_i2_dmp2	2.17	-0.08	2.62	0.37	1.70	-0.55	2.77	0.52	3.32	1.07
cu2_i2_dppt12	3.02	-0.74	3.81	0.05	2.84	-0.92	3.83	0.07	4.23	0.47
cu2_i2_dppt22	2.99	-0.66	3.82	0.17	2.79	-0.86	3.84	0.19	4.28	0.63
cu2_scn2_dppb2	2.91	-0.08	3.68	0.69	2.74	-0.25	3.72	0.73	4.05	1.06
cu2_scn2_pop2	3.48	0.22	4.39	1.13	3.24	-0.02	4.42	1.16	4.79	1.53
cu_br_ttpp	2.77	-0.35	3.49	0.37	2.60	-0.51	3.54	0.42	3.93	0.82
cu_cl_ttpp	2.80	-0.31	3.48	0.36	2.65	-0.46	3.53	0.42	3.92	0.80
cu_i_ttpp	2.61	-0.50	3.49	0.38	2.40	-0.72	3.50	0.38	3.95	0.83
cu_i_ttppo	2.48	-0.67	3.59	0.44	2.22	-0.92	3.52	0.37	4.15	1.01

cu_scn_ttpp	2.72	-0.30	3.59	0.57	2.51	-0.52	3.62	0.60	4.03	1.01
cu_ccph_ttpp	2.83	-0.16	3.50	0.51	2.69	-0.30	3.57	0.58	3.89	0.91
cu_ccnph_ttpp	2.93	0.79	3.60	1.46	2.74	0.60	3.64	1.50	4.03	1.89
cu_ccpyr_ttpp	2.88	-0.11	3.56	0.58	2.73	-0.26	3.60	0.61	3.97	0.98

Table S6. Absolute differences in $S_0 \rightarrow S_1$ excitation energies (eV) between the isolated and QM/MM models.

	PBE0	LRC-ωPBEh	B3LYP	CAM-B3LYP	ωB97X
cu2_i2_pnme22	0.19	0.25	0.14	0.28	0.02
cu2_i2_phen2	0.04	0.17	0.04	0.15	0.17
cu2_i2_dmp2	0.00	0.00	0.05	0.12	0.02
cu2_i2_dppt12	0.04	0.02	0.01	0.09	0.02
cu2_i2_dppt22	0.11	0.04	0.12	0.07	0.04
cu2_scn2_dppb2	0.18	0.13	0.17	0.14	0.20
cu2_scn2_pop2	0.03	0.09	0.01	0.10	0.03
cu_br_ttpp	0.08	0.02	0.07	0.04	0.03
cu_cl_ttpp	0.07	0.04	0.08	0.05	0.03
cu_i_ttpp	0.11	0.03	0.12	0.07	0.02
cu_i_ttppo	0.02	0.01	0.01	0.13	0.04
cu_scn_ttpp	0.02	0.02	0.02	0.02	0.03
cu_ccph_ttpp	0.07	0.08	0.04	0.03	0.12
cu_ccnph_ttpp	0.00	0.00	0.03	0.01	0.00
cu_ccpyr_ttpp	0.01	0.02	0.02	0.00	0.03

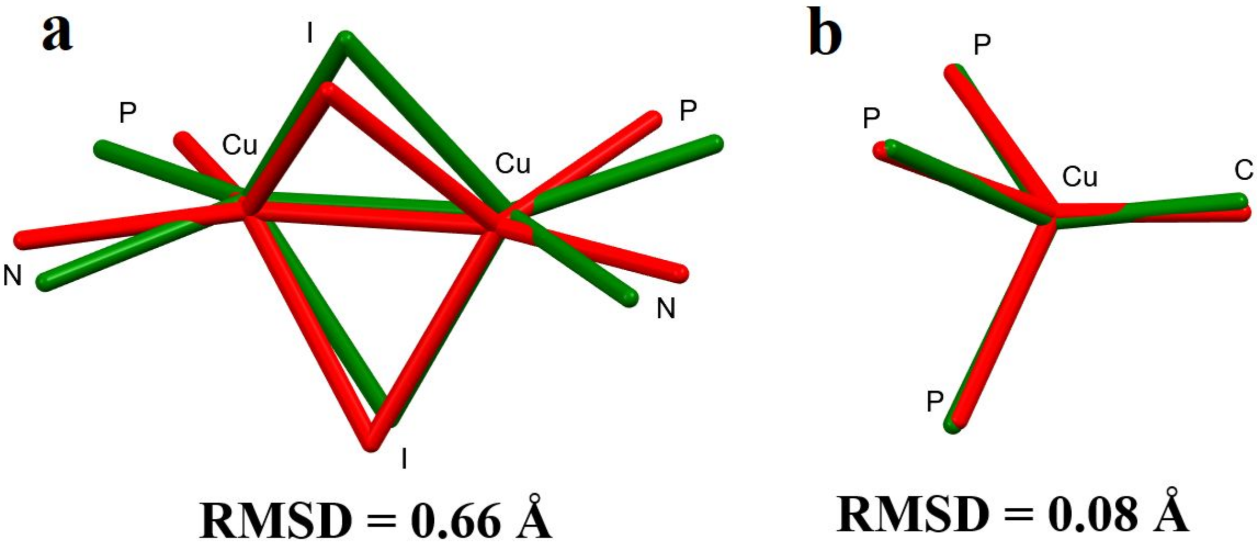

Figure S1. Superimposed Cu coordination environments from optimized (LRC-ωPBEh) ground state geometries of **[$Cu_2I_2(pnme)_2$]** (a) and **[Cu(ccpyr)(ttpp)]** (b). Red = QM/MM model, green = isolated model.

Table S7. RMSD (Å) between the optimized ground and excited state structures with the PBE0 functional.

	PBE0					
	ISOLATED			QM/MM		
	S0-S1	S0-T1	S1-T1	S0-S1	S0-T1	S1-T1
cu_pop_pypz	0.58	0.59	0.17	0.18	0.17	0.05
cu_pop_pympz	0.47	0.55	0.17	0.32	0.28	0.06
cu_pop_pytfmpz	0.36	0.35	0.15	0.17	0.13	0.07
cu_pop_bpy	0.64	0.69	0.11	0.19	0.20	0.05
cu_pop_mbpy	0.35	0.37	0.14	0.14	0.14	0.03
cu_pop_dmbpy	0.58	0.28	0.42	0.13	0.00	0.07
cu_pop_etbpy	0.41	0.34	0.14	0.15	0.16	0.04
cu_pop_phbpy	0.42	0.32	0.12	0.18	0.15	0.06
cu_pop_tfmbpy	0.72	0.65	0.13	0.37	0.32	0.08
cu_pop_55dtfmbpy	0.40	0.46	0.17	0.12	0.13	0.05
cu_pop_44dtfmbpy	0.65	0.77	0.17	0.27	0.26	0.08
cu_xantphos_tfmbpy	0.41	0.37	0.20	0.11	0.10	0.07
cu_xantphos_phbpy	0.70	0.66	0.11	0.16	0.18	0.31
cu_xantphos_dmbpy	0.40	0.33	0.13	0.12	0.12	0.03
cu_xantphos_44dtfmbpy	0.82	0.78	0.14	0.15	0.12	0.23
cu_xantphos_bpy	0.46	0.45	0.11	0.22	0.17	0.06
cu_xantphos_etbpy	0.33	0.69	0.56	0.11	0.14	0.10
cu_xantphos_mbpy	0.35	0.69	0.49	0.12	0.12	0.06
cu2_cl2_dpmb2	0.58	0.44	0.26	0.10	0.11	0.07
cu2_cl2_dpmt2	0.97	2.25	1.68	0.25	0.13	0.25
cu2_cl2_dppb2	1.74	0.75	1.36	0.09	0.07	0.04
cu2_cl2_pnme22	1.59	2.54	2.44	0.10	0.10	0.03
cu2_br2_dpmb2	1.96	0.87	1.63	0.10	0.11	0.09
cu2_br2_dpmt2	0.87	0.30	0.96	0.20	0.18	0.05
cu2_br2_dppb2	1.77	0.83	1.39	0.08	0.07	0.10
cu2_br2_pnme22	1.92	1.56	1.75	0.17	0.10	0.16
cu2_i2_dpmb2	1.23	1.03	1.79	0.24	0.18	0.31
cu2_i2_dpmt2	1.17	1.36	1.93	0.21	0.18	0.05
cu2_i2_dppb2	1.10	0.78	0.42	0.08	0.07	0.09
cu2_i2_pnme22	1.47	1.74	0.99	0.16	0.15	0.04
cu2_i2_pnpy22	0.81	0.79	1.19	0.09	0.08	0.02
cu2_i2_phen2	2.35	2.08	0.81	0.10	0.09	0.05
cu2_i2_dmp2	0.44	0.48	0.75	0.34	0.13	0.31
cu2_i2_dppt12	0.56	0.32	0.43	0.09	0.07	0.04
cu2_i2_dppt22	1.84	2.41	2.52	0.36	0.33	0.04
cu2_scn2_dppb2	1.83	2.12	2.24	0.10	0.09	0.03
cu2_scn2_pop2	0.86	1.20	1.68	0.11	0.10	0.12
cu_br_dmpzpp	0.68	0.61	0.09	0.10	0.10	0.03
cu_br_tpypo	0.13	0.16	0.05	0.10	0.10	0.02
cu_br_ttpp	1.40	1.55	0.21	0.11	0.10	0.02
cu_cl_tpypo	0.14	0.15	0.04	0.07	0.07	0.02
cu_cl_ttpp	1.35	1.58	0.70	0.11	0.10	0.02
cu_i_dmpzpp	0.46	0.49	0.40	0.18	0.17	0.07
cu_i_tpyaso	0.16	0.18	0.06	0.06	0.07	0.03
cu_i_tpym	0.14	0.17	0.04	0.05	0.05	0.02
cu_i_tpypo	0.16	0.14	0.06	0.05	0.06	0.03

cu_i_tpyps	0.16	0.16	0.05	0.06	0.07	0.03
cu_i_tpypse	0.13	0.14	0.03	0.09	0.08	0.03
cu_i_ttpp	1.43	1.58	0.22	0.12	0.11	0.02
cu_i_ttppo	0.70	0.81	0.23	0.20	0.15	0.08
cu_cn_ttpp	1.31	1.41	0.18	0.12	0.11	0.02
cu_scn_ttpp	1.30	1.55	0.73	0.12	0.11	0.04
cu_sph_dmpzpp	0.42	0.61	0.34	0.20	0.21	0.03
cu_ccph_ttpp	1.88	2.02	0.29	0.16	0.17	0.04
cu_ccnph_ttpp	1.78	0.29	1.80	0.10	0.02	0.10
cu_ccpyr_ttpp	1.56	1.85	0.78	0.13	0.17	0.06

Table S8. RMSD (Å) between the optimized ground and excited state structures with the LRC-ωPBEh functional.

	LRC-ωPBEh					
	ISOLATED			QM/MM		
	S0-S1	S0-T1	S1-T1	S0-S1	S0-T1	S1-T1
cu_pop_pypz	0.60	0.55	0.13	0.16	0.15	0.05
cu_pop_pympz	0.48	0.57	0.20	0.31	0.26	0.07
cu_pop_pytfmpz	0.79	0.47	0.96	0.16	0.15	0.07
cu_pop_bpy	0.85	0.92	0.13	0.17	0.17	0.11
cu_pop_mbpy	0.36	0.42	0.13	0.13	0.14	0.04
cu_pop_dmbpy	0.51	0.43	0.27	0.12	0.07	0.10
cu_pop_etbpy	0.39	0.34	0.13	0.12	0.03	0.13
cu_pop_phbpy	0.40	0.31	0.12	0.17	0.14	0.05
cu_pop_tfmbpy	0.42	0.42	0.13	0.30	0.18	0.17
cu_pop_55dtfmbpy	0.57	0.72	0.22	0.12	0.14	0.05
cu_pop_44dtfmbpy	0.63	0.69	0.22	0.18	0.21	0.05
cu_xantphos_tfmbpy	0.41	0.36	0.60	0.11	0.12	0.07
cu_xantphos_phbpy	0.76	0.74	0.11	0.21	0.18	0.05
cu_xantphos_dmbpy	0.36	0.27	0.53	0.11	0.12	0.03
cu_xantphos_44dtfmbpy	0.80	0.74	0.13	0.16	0.13	0.09
cu_xantphos_bpy	0.42	0.46	0.13	0.20	0.17	0.05
cu_xantphos_etbpy	0.34	0.44	0.20	0.10	0.14	0.05
cu_xantphos_mbpy	0.35	0.43	0.17	0.09	0.12	0.06
cu2_cl2_dpmb2	1.30	1.87	1.44	0.12	0.10	0.04
cu2_cl2_dpmt2	2.57	0.36	2.59	0.25	0.11	0.24
cu2_cl2_dppb2	1.68	0.45	1.62	0.09	0.08	0.11
cu2_cl2_pnme22	1.38	2.59	2.15	0.09	0.10	0.04
cu2_br2_dpmb2	1.94	0.55	1.68	0.11	0.10	0.03
cu2_br2_dpmt2	1.13	0.57	1.05	0.16	0.06	0.15
cu2_br2_dppb2	1.72	0.38	1.65	0.08	0.08	0.11
cu2_br2_pnme22	1.52	1.01	1.97	0.14	0.10	0.06
cu2_i2_dpmb2	0.87	1.48	0.65	0.17	0.21	0.20
cu2_i2_dpmt2	0.49	0.31	0.40	0.22	0.17	0.16
cu2_i2_dppb2	1.81	0.39	1.62	0.08	0.08	0.07
cu2_i2_pnme22	1.68	0.44	1.36	0.15	0.15	0.13
cu2_i2_pnpy22	0.66	0.41	0.87	0.09	0.08	0.03
cu2_i2_phen2	1.95	1.87	0.91	0.07	0.09	0.05
cu2_i2_dmp2	0.24	0.43	0.35	0.09	0.10	0.04
cu2_i2_dppt12	0.38	0.24	0.35	0.10	0.06	0.12
cu2_i2_dppt22	1.80	0.24	1.88	0.34	0.10	0.37

cu2_scn2_dppb2	1.36	1.28	0.72	0.11	0.10	0.14
cu2_scn2_pop2	0.90	0.48	1.17	0.13	0.08	0.15
cu_br_dmpzpp	0.82	0.80	0.09	0.11	0.10	0.04
cu_br_tpypo	0.14	0.15	0.04	0.10	0.10	0.02
cu_br_ttpp	1.53	1.40	0.26	0.12	0.11	0.02
cu_cl_tpypo	0.14	0.15	0.04	0.07	0.07	0.03
cu_cl_ttpp	1.50	1.40	0.22	0.11	0.10	0.02
cu_i_dmpzpp	0.81	0.80	0.10	0.21	0.19	0.06
cu_i_tpyaso	0.16	0.14	0.09	0.08	0.09	0.04
cu_i_tpym	0.14	0.17	0.07	0.05	0.06	0.02
cu_i_tpypo	0.16	0.13	0.07	0.07	0.08	0.04
cu_i_tpyps	0.16	0.15	0.06	0.08	0.09	0.02
cu_i_tpypse	0.13	0.12	0.05	0.09	0.09	0.04
cu_i_ttpp	1.36	1.34	0.23	0.14	0.12	0.03
cu_i_ttppo	0.51	0.66	0.32	0.22	0.17	0.08
cu_cn_ttpp	1.37	1.23	0.38	0.12	0.11	0.02
cu_scn_ttpp	1.31	1.14	0.39	0.16	0.12	0.08
cu_sph_dmpzpp	0.42	0.53	0.30	0.21	0.17	0.11
cu_ccph_ttpp	1.88	0.15	1.85	0.17	0.18	0.04
cu_ccnph_ttpp	1.22	0.18	1.24	0.10	0.02	0.10
cu_ccpyr_ttpp	1.69	0.27	1.86	0.15	0.03	0.14

Table S9. RMSD (Å) between the optimized ground and excited state structures with the B3LYP functional.

	B3LYP					
	ISOLATED			QM/MM		
	S0-S1	S0-T1	S1-T1	S0-S1	S0-T1	S1-T1
cu_pop_pypz	0.54	0.51	0.23	0.15	0.12	0.06
cu_pop_pympz	0.49	0.53	0.16	0.32	0.28	0.07
cu_pop_pytfmpz	0.33	0.32	0.16	0.15	0.12	0.08
cu_pop_bpy	0.59	0.57	0.12	0.20	0.17	0.17
cu_pop_mbpy	0.34	0.33	0.16	0.12	0.12	0.03
cu_pop_dmbpy	0.48	0.30	0.30	0.13	0.13	0.07
cu_pop_etbpy	0.47	0.39	0.34	0.13	0.13	0.05
cu_pop_phbpy	0.36	0.30	0.34	0.18	0.15	0.06
cu_pop_tfmbpy	0.46	0.38	0.32	0.39	0.37	0.10
cu_pop_55dtfmbpy	0.73	0.64	0.21	0.12	0.12	0.05
cu_pop_44dtfmbpy	0.70	0.61	0.19	0.22	0.18	0.08
cu_xantphos_tfmbpy	0.30	0.32	0.18	0.10	0.10	0.08
cu_xantphos_phbpy	1.05	0.41	1.11	0.15	0.18	0.30
cu_xantphos_dmbpy	0.44	0.40	0.11	0.11	0.11	0.05
cu_xantphos_44dtfmbpy	1.19	0.62	0.98	0.14	0.13	0.23
cu_xantphos_bpy	0.54	0.44	0.18	0.23	0.17	0.07
cu_xantphos_etbpy	0.55	0.33	0.42	0.10	0.10	0.04
cu_xantphos_mbpy	0.52	0.52	0.19	0.11	0.12	0.06
cu2_cl2_dpmb2	1.87	0.87	2.27	0.10	0.10	0.09
cu2_cl2_dpmt2	1.36	1.80	1.36	0.23	0.12	0.24
cu2_cl2_dppb2	1.65	1.70	2.24	0.10	0.07	0.05
cu2_cl2_pnme22	1.92	1.15	2.48	0.09	0.10	0.04
cu2_br2_dpmb2	1.83	1.41	1.48	0.10	0.10	0.10
cu2_br2_dpmt2	0.76	1.58	1.84	0.27	0.29	0.07

cu2_br2_dppb2	1.62	1.89	2.09	0.07	0.05	0.08
cu2_br2_pnme22	1.76	0.90	1.90	0.28	0.09	0.33
cu2_i2_dpmb2	0.77	2.11	2.23	0.18	0.14	0.20
cu2_i2_dpmt2	1.23	1.87	1.95	0.23	0.21	0.06
cu2_i2_dppb2	1.08	2.40	2.41	0.09	0.07	0.09
cu2_i2_pnme22	1.58	1.13	1.34	0.09	0.07	0.04
cu2_i2_pnpy22	1.63	2.67	2.85	0.08	0.08	0.03
cu2_i2_phen2	0.79	1.05	1.22	0.09	0.08	0.03
cu2_i2_dmp2	0.18	0.39	0.46	0.28	0.29	0.02
cu2_i2_dppt12	1.65	2.18	1.48	0.08	0.07	0.04
cu2_i2_dppt22	1.71	2.42	2.45	0.11	0.08	0.04
cu2_scn2_dppb2	1.79	2.45	2.69	0.09	0.07	0.04
cu2_scn2_pop2	0.97	1.00	1.18	0.11	0.11	0.11
cu_br_dmpzpp	0.66	0.58	0.70	0.10	0.14	0.07
cu_br_tpypo	0.18	0.19	0.04	0.06	0.07	0.02
cu_br_ttpp	1.30	1.55	0.76	0.10	0.09	0.03
cu_cl_tpypo	0.14	0.15	0.04	0.07	0.07	0.02
cu_cl_ttpp	1.28	1.60	0.64	0.10	0.08	0.03
cu_i_dmpzpp	0.55	0.63	0.76	0.26	0.24	0.15
cu_i_tpyaso	0.14	0.15	0.07	0.06	0.06	0.04
cu_i_tpym	0.12	0.12	0.07	0.05	0.05	0.02
cu_i_tpypo	0.10	0.12	0.04	0.05	0.06	0.03
cu_i_tpyps	0.13	0.16	0.04	0.05	0.07	0.05
cu_i_tpypse	0.13	0.15	0.04	0.07	0.08	0.03
cu_i_ttpp	1.33	1.78	0.58	0.11	0.10	0.03
cu_i_ttppo	0.51	1.94	1.78	0.15	0.14	0.10
cu_cn_ttpp	1.26	1.46	0.71	0.11	0.09	0.02
cu_scn_ttpp	1.17	1.50	0.82	0.09	0.14	0.07
cu_sph_dmpzpp	0.54	0.83	0.51	0.19	0.19	0.13
cu_ccph_ttpp	1.73	1.93	1.23	0.23	0.19	0.06
cu_ccnph_ttpp	1.65	0.48	1.74	0.09	0.02	0.09
cu_ccpyr_ttpp	1.43	1.77	0.81	0.12	0.19	0.11

Table S10. RMSD (Å) between the optimized ground and excited state structures with the CAM-B3LYP functional.

	CAM-B3LYP					
	ISOLATED			QM/MM		
	S0-S1	S0-T1	S1-T1	S0-S1	S0-T1	S1-T1
cu_pop_pypz	0.61	0.52	0.21	0.16	0.13	0.06
cu_pop_pympz	0.51	0.57	0.17	0.31	0.27	0.07
cu_pop_pytfmpz	0.37	0.36	0.15	0.16	0.12	0.08
cu_pop_bpy	0.80	0.82	0.11	0.17	0.18	0.03
cu_pop_mbpy	0.36	0.40	0.15	0.13	0.14	0.03
cu_pop_dmbpy	0.42	0.26	0.33	0.13	0.14	0.07
cu_pop_etbpy	0.48	0.43	0.33	0.14	0.18	0.06
cu_pop_phbpy	0.37	0.22	0.24	0.17	0.15	0.06
cu_pop_tfmbpy	0.45	0.34	0.25	0.33	0.29	0.11
cu_pop_55dtfmbpy	1.00	0.99	0.16	0.12	0.13	0.04
cu_pop_44dtfmbpy	0.77	0.85	0.21	0.20	0.22	0.07
cu_xantphos_tfmbpy	0.30	0.35	0.15	0.11	0.12	0.06
cu_xantphos_phbpy	1.02	0.64	1.28	0.21	0.17	0.06

cu_xantphos_dmbpy	0.42	0.39	0.13	0.10	0.11	0.04
cu_xantphos_44dtfmbpy	1.46	0.74	1.42	0.14	0.14	0.23
cu_xantphos_bpy	0.83	0.81	0.18	0.21	0.16	0.06
cu_xantphos_etbpy	0.58	0.42	0.48	0.09	0.14	0.09
cu_xantphos_mbpy	0.57	0.61	0.23	0.10	0.13	0.05
cu2_cl2_dpmb2	1.72	2.46	2.36	0.10	0.10	0.09
cu2_cl2_dpmt2	2.54	0.70	2.64	0.26	0.11	0.27
cu2_cl2_dppb2	1.75	2.16	2.57	0.10	0.07	0.06
cu2_cl2_pnme22	1.51	1.17	2.30	0.09	0.11	0.07
cu2_br2_dpmb2	1.77	2.14	1.10	0.11	0.10	0.11
cu2_br2_dpmt2	0.77	0.73	0.39	0.29	0.26	0.14
cu2_br2_dppb2	1.72	2.05	1.88	0.08	0.07	0.10
cu2_br2_pnme22	1.48	1.07	1.21	0.20	0.18	0.33
cu2_i2_dpmb2	1.91	1.52	2.35	0.21	0.12	0.24
cu2_i2_dpmt2	1.28	0.42	1.15	0.25	0.19	0.13
cu2_i2_dppb2	1.90	2.03	2.05	0.10	0.07	0.12
cu2_i2_pnme22	1.54	1.15	0.84	0.09	0.06	0.05
cu2_i2_pnpy22	0.69	0.31	0.78	0.09	0.08	0.03
cu2_i2_phen2	2.14	1.96	1.69	0.08	0.12	0.07
cu2_i2_dmp2	0.33	0.41	0.12	0.30	0.33	0.07
cu2_i2_dppt12	1.52	0.97	1.56	0.10	0.05	0.12
cu2_i2_dppt22	1.90	0.63	2.01	0.13	0.09	0.10
cu2_scn2_dppb2	1.54	2.42	2.54	0.11	0.09	0.05
cu2_scn2_pop2	1.65	0.59	1.81	0.13	0.09	0.16
cu_br_dmpzpp	0.89	0.48	0.74	0.11	0.12	0.06
cu_br_tpypo	0.18	0.19	0.04	0.07	0.08	0.02
cu_br_ttpp	1.45	1.51	0.17	0.12	0.09	0.05
cu_cl_tpypo	0.14	0.15	0.04	0.07	0.07	0.02
cu_cl_ttpp	1.45	1.57	0.26	0.11	0.09	0.05
cu_i_dmpzpp	0.81	0.48	0.56	0.26	0.22	0.14
cu_i_tpyaso	0.16	0.18	0.08	0.07	0.08	0.05
cu_i_tpym	0.14	0.13	0.10	0.05	0.06	0.03
cu_i_tpypo	0.15	0.12	0.06	0.06	0.08	0.04
cu_i_tpyps	0.15	0.14	0.05	0.07	0.08	0.03
cu_i_tpypse	0.11	0.11	0.03	0.09	0.08	0.04
cu_i_ttpp	1.39	1.46	0.17	0.14	0.11	0.05
cu_i_ttppo	0.49	1.89	1.71	0.20	0.17	0.08
cu_cn_ttpp	1.27	1.18	0.36	0.12	0.09	0.04
cu_scn_ttpp	1.20	1.53	0.80	0.15	0.08	0.09
cu_sph_dmpzpp	0.63	0.79	0.38	0.19	0.15	0.10
cu_ccph_ttpp	1.70	1.90	0.51	0.25	0.21	0.06
cu_ccnph_ttpp	1.28	0.26	1.32	0.10	0.02	0.10
cu_ccpyr_ttpp	1.34	1.62	0.57	0.17	0.20	0.08

Table S11. RMSD (Å) between the optimized ground and excited state structures with the ωB97X functional.

	ωB97X					
	ISOLATED			QM/MM		
	S0-S1	S0-T1	S1-T1	S0-S1	S0-T1	S1-T1
cu_pop_pypz	0.65	0.38	0.36	0.17	0.11	0.08
cu_pop_pympz	1.17	1.14	0.20	0.37	0.34	0.09

cu_pop_pytfmpz	0.47	0.39	0.41	0.17	0.11	0.09
cu_pop_bpy	1.08	0.38	1.04	0.17	0.05	0.19
cu_pop_mbpy	0.42	0.47	0.20	0.12	0.12	0.04
cu_pop_dmbpy	0.55	0.26	0.46	0.14	0.16	0.08
cu_pop_etbpy	0.48	0.42	0.35	0.15	0.19	0.07
cu_pop_phbpy	0.61	0.35	0.42	0.17	0.16	0.07
cu_pop_tfmbpy	0.66	0.52	0.19	0.30	0.26	0.11
cu_pop_55dtfmbpy	0.64	0.52	0.31	0.12	0.13	0.04
cu_pop_44dtfmbpy	0.63	0.52	0.18	0.20	0.22	0.07
cu_xantphos_tfmbpy	0.30	0.37	0.15	0.11	0.13	0.07
cu_xantphos_phbpy	1.09	0.62	1.35	0.20	0.17	0.06
cu_xantphos_dmbpy	0.47	0.35	0.24	0.10	0.11	0.05
cu_xantphos_44dtfmbpy	1.71	0.79	1.67	0.15	0.17	0.13
cu_xantphos_bpy	1.08	0.96	0.43	0.21	0.16	0.08
cu_xantphos_etbpy	0.56	0.68	0.58	0.09	0.15	0.11
cu_xantphos_mbpy	0.60	0.61	0.34	0.09	0.13	0.07
cu2_cl2_dpmb2	1.53	0.61	1.37	0.13	0.09	0.13
cu2_cl2_dpmt2	0.65	0.66	0.35	0.32	0.12	0.31
cu2_cl2_dppb2	0.32	1.18	1.33	0.12	0.07	0.09
cu2_cl2_pnme22	1.34	0.93	0.83	0.10	0.11	0.07
cu2_br2_dpmb2	1.36	0.58	1.13	0.13	0.10	0.12
cu2_br2_dpmt2	0.60	1.06	0.95	0.27	0.27	0.16
cu2_br2_dppb2	0.33	0.50	0.65	0.10	0.09	0.12
cu2_br2_pnme22	0.76	0.88	0.62	0.17	0.21	0.33
cu2_i2_dpmb2	1.23	0.83	1.67	0.26	0.11	0.31
cu2_i2_dpmt2	0.42	0.47	0.26	0.29	0.24	0.12
cu2_i2_dppb2	0.90	0.44	0.86	0.13	0.09	0.15
cu2_i2_pnme22	0.34	0.78	0.78	0.11	0.15	0.18
cu2_i2_pnpy22	0.57	0.85	0.52	0.10	0.23	0.26
cu2_i2_phen2	0.27	0.57	0.91	0.10	0.12	0.06
cu2_i2_dmp2	0.19	0.36	0.40	0.23	0.22	0.03
cu2_i2_dppt12	0.32	0.14	0.31	0.11	0.07	0.13
cu2_i2_dppt22	0.93	0.58	1.02	0.38	0.34	0.13
cu2_scn2_dppb2	1.72	2.71	2.82	0.12	0.10	0.06
cu2_scn2_pop2	1.63	1.13	1.96	0.15	0.07	0.15
cu_br_dmpzpp	1.00	0.43	0.80	0.13	0.15	0.07
cu_br_tpypo	0.09	0.13	0.07	0.08	0.08	0.02
cu_br_ttpp	1.28	1.29	0.31	0.13	0.10	0.07
cu_cl_tpypo	0.13	0.14	0.06	0.08	0.08	0.03
cu_cl_ttpp	0.73	1.04	0.47	0.13	0.10	0.07
cu_i_dmpzpp	0.89	0.84	0.13	0.30	0.25	0.15
cu_i_tpyaso	0.15	0.15	0.04	0.08	0.09	0.04
cu_i_tpym	0.13	0.13	0.10	0.06	0.06	0.03
cu_i_tpypo	0.13	0.13	0.04	0.08	0.08	0.03
cu_i_tpyps	0.13	0.12	0.06	0.08	0.09	0.03
cu_i_tpypse	0.10	0.11	0.04	0.08	0.08	0.04
cu_i_ttpp	0.98	1.06	0.28	0.15	0.12	0.07
cu_i_ttppo	0.74	1.63	1.22	0.21	0.21	0.11
cu_cn_ttpp	0.90	1.09	0.48	0.12	0.08	0.06
cu_scn_ttpp	1.35	0.37	1.28	0.17	0.09	0.13
cu_sph_dmpzpp	0.91	1.08	0.90	0.22	0.19	0.09
cu_ccph_ttpp	1.25	2.41	1.45	0.16	0.13	0.06

cu_ccnph_ttpp	1.32	0.59	1.15	0.11	0.03	0.11
cu_ccpyr_ttpp	1.14	2.04	1.24	0.16	0.19	0.11

Table S12. Average RMSD (Å) between the optimized ground state (S_0) and excited triplet state (T_1) structures. $N = 56$.

ISOLATED	PBE0	LRC-ωPBEh	B3LYP	CAM-B3LYP	ωB97X
[Cu(NN)(PP)]	0.52	0.52	0.44	0.54	0.54
$[Cu_2X_2(L^2)_2]$	1.26	0.81	1.63	1.31	0.80
$[CuX(L^3)]$	0.81	0.57	0.90	0.83	0.78
**All**	0.87	0.63	1.00	0.90	0.71

QM/MM	PBE0	LRC-ωPBEh	B3LYP	CAM-B3LYP	ωB97X
[Cu(NN)(PP)]	0.16	0.15	0.16	0.16	0.16
$[Cu_2X_2(L^2)_2]$	0.12	0.10	0.12	0.12	0.15
$[CuX(L^3)]$	0.11	0.11	0.11	0.11	0.12
**All**	0.13	0.12	0.13	0.13	0.14

Table S13. Average RMSD (Å) between the optimized excited singlet (S_1) and triplet (T_1) state structures. $N = 56$.

ISOLATED	PBE0	LRC-ωPBEh	B3LYP	CAM-B3LYP	ωB97X
[Cu(NN)(PP)]	0.20	0.25	0.32	0.34	0.49
$[Cu_2X_2(L^2)_2]$	1.38	1.29	1.89	1.65	0.99
$[CuX(L^3)]$	0.33	0.40	0.60	0.42	0.53
**All**	0.65	0.65	0.95	0.81	0.67

QM/MM	PBE0	LRC-ωPBEh	B3LYP	CAM-B3LYP	ωB97X
[Cu(NN)(PP)]	0.08	0.07	0.09	0.07	0.08
$[Cu_2X_2(L^2)_2]$	0.10	0.12	0.09	0.12	0.15
$[CuX(L^3)]$	0.04	0.05	0.06	0.06	0.07
**All**	0.07	0.08	0.08	0.08	0.10

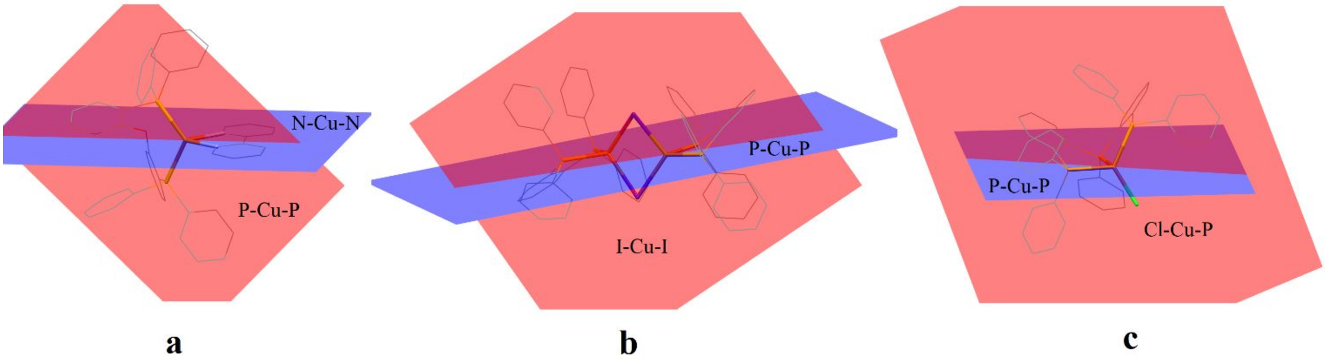

Figure S2. Planes defining the flattening angle for **[Cu(pop)(bpy)]** (a), **$[Cu_2I_2(dppb)_2]$** (b) and **[CuCl(ttpp)]** (c).

Table S14. Flattening angles (FA) and degrees (FD) for the isolated and QM/MM models with the LRC-ωPBEh functional.

	Isolated			QM/MM		
	FA (S_0) [°]	FA (S_1) [°]	FD [°]	FA (S_0) [°]	FA (S_1) [°]	FD [°]
cu_pop_pypz	84.4	52.0	32.4	77.1	59.5	17.6
cu_pop_pympz	89.3	60.0	29.3	85.1	68.0	17.1
cu_pop_pytfmpz	88.7	60.9	27.8	84.5	69.8	14.7
cu_pop_bpy	84.2	55.7	28.5	89.5	83.2	6.3
cu_pop_mbpy	88.1	66.3	21.8	85.0	76.1	8.9
cu_pop_dmbpy	82.3	69.8	12.5	82.9	70.7	12.2
cu_pop_etbpy	79.9	60.2	19.7	88.4	79.9	8.5
cu_pop_phbpy	78.7	64.0	14.7	77.1	66.2	10.9
cu_pop_tfmbpy	84.6	71.0	13.6	80.6	68.1	12.5
cu_pop_55dtfmbpy	87.8	58.0	29.8	83.0	71.6	11.4
cu_pop_44dtfmbpy	86.7	55.7	31.0	87.3	71.9	15.4
cu_xantphos_tfmbpy	88.7	74.6	14.1	87.6	79.9	7.7
cu_xantphos_phbpy	84.8	61.7	23.1	85.2	69.4	15.8
cu_xantphos_dmbpy	85.5	71.6	13.9	86.1	78.3	7.8
cu_xantphos_44dtfmbpy	83.7	54.6	29.1	87.2	75.0	12.2
cu_xantphos_bpy	86.8	57.5	29.3	79.7	58.7	21.0
cu_xantphos_etbpy	89.5	71.6	17.9	89.5	82.0	7.5
cu_xantphos_mbpy	89.4	71.2	18.2	89.5	81.7	7.8
cu2_cl2_dpmb2	88.1	84.8	3.3	85.5	79.5	6.0
cu2_cl2_dpmt2	84.8	8.3	76.5	87.2	85.7	1.5
cu2_cl2_dppb2	87.1	57.4	29.7	88.3	88.0	0.3
cu2_cl2_pnme22	85.4	47.8	37.6	84.4	75.6	8.8
cu2_br2_dpmb2	86.4	51.1	35.3	85.4	80.7	4.7
cu2_br2_dpmt2	88.8	60.7	28.1	84.1	83.5	0.6
cu2_br2_dppb2	87.4	57.2	30.2	88.1	88.6	-0.5
cu2_br2_pnme22	88.0	62.3	25.7	82.8	82.1	0.7
cu2_i2_dpmb2	88.0	55.7	32.3	88.8	84.2	4.6
cu2_i2_dpmt2	87.5	84.4	3.1	88.0	88.8	-0.8
cu2_i2_dppb2	84.4	59.8	24.6	85.9	84.9	1.0
cu2_i2_pnme22	88.7	68.8	19.9	82.7	80.0	2.7
cu2_i2_pnpy22	80.3	62.5	17.8	88.4	88.4	0.0
cu2_i2_phen2	81.9	9.0	72.9	83.2	75.5	7.7
cu2_i2_dmp2	88.6	87.5	1.1	89.0	87.8	1.2
cu2_i2_dppt12	84.5	72.1	12.4	88.8	89.8	-1.0
cu2_i2_dppt22	84.2	52.0	32.2	86.1	84.4	1.7
cu2_scn2_dppb2	86.6	57.4	29.2	81.5	74.5	7.0
cu2_scn2_pop2	87.0	69.1	17.9	88.9	86.3	2.6
cu_br_dmpzpp	75.3	36.9	38.4	77.2	75.8	1.4
cu_br_tpypo	86.9	87.5	-0.6	89.7	89.8	-0.1
cu_br_ttpp	69.3	44.2	25.1	73.8	68.8	5.0
cu_cl_tpypo	89.5	87.6	1.9	85.5	85.8	-0.3
cu_cl_ttpp	71.6	44.3	27.3	73.4	68.6	4.8

cu_i_dmpzpp	74.3	37.8	36.5	75.7	67.2	8.5
cu_i_tpyaso	89.4	85.7	3.7	84.7	84.0	0.7
cu_i_tpym	89.1	89.5	-0.4	88.0	88.9	-0.9
cu_i_tpypo	89.5	86.8	2.7	85.5	85.2	0.3
cu_i_tpyps	89.9	87.5	2.4	85.8	87.0	-1.2
cu_i_tpypse	89.8	87.7	2.1	88.5	88.3	0.2
cu_i_ttpp	73.2	36.1	37.1	74.0	68.6	5.4
cu_i_ttppo	69.5	52.6	16.9	78.1	75.0	3.1
cu_cn_ttpp	73.1	46.3	26.8	72.9	66.6	6.3
cu_scn_ttpp	70.8	47.0	23.8	68.1	62.0	6.1
cu_sph_dmpzpp	73.5	68.2	5.3	72.4	70.8	1.6
cu_ccph_ttpp	71.5	45.7	25.8	66.3	58.6	7.7
cu_ccnph_ttpp	71.4	50.4	21.0	72.8	67.6	5.2
cu_ccpyr_ttpp	71.1	46.8	24.3	68.9	61.7	7.2

Table S15. Predicted $S_1 \rightarrow S_0$ fluorescence energies with the isolated models.

	ISOLATED										
	PBE0		LRC-ωPBEh		B3LYP		CAM-B3LYP		ωB97X		EXP
	E [eV]	Error [eV]	E [eV]	Error [eV]	E [eV]	Error [eV]	E [eV]	Error [eV]	E [eV]	Error [eV]	E [eV]
cu_pop_pypz	1.78	-0.75	2.23	-0.30	1.73	-0.80	2.37	-0.16	2.63	0.10	2.53
cu_pop_pympz	1.85	-0.81	2.36	-0.30	1.78	-0.89	2.47	-0.20	2.73	0.06	2.67
cu_pop_pytfmpz	1.72	-0.80	2.18	-0.34	1.63	-0.89	2.29	-0.23	2.60	0.08	2.52
cu_pop_bpy	1.33	-0.81	1.72	-0.42	1.28	-0.86	1.84	-0.30	2.02	-0.12	2.13
cu_pop_mbpy	1.58	-0.60	1.98	-0.20	1.51	-0.67	2.10	-0.09	2.32	0.13	2.19
cu_pop_dmbpy	1.89	-0.43	2.27	-0.04	1.78	-0.53	2.38	0.07	2.63	0.31	2.32
cu_pop_etbpy	1.62	-0.61	2.00	-0.23	1.55	-0.68	2.12	-0.11	2.37	0.15	2.23
cu_pop_phbpy	1.60	-0.56	1.94	-0.21	1.51	-0.64	2.07	-0.09	2.28	0.13	2.15
cu_pop_tfmbpy	1.49	-0.67	1.95	-0.21	1.40	-0.76	1.95	-0.20	2.21	0.05	2.16
cu_pop_55dtfmbpy	1.03	-0.89	1.48	-0.43	1.05	-0.86	1.64	-0.27	1.89	-0.02	1.91
cu_pop_44dtfmbpy	1.09	-0.78	1.61	-0.26	1.14	-0.72	1.71	-0.15	2.01	0.14	1.87
cu_xantphos_tfmbpy	1.58	-0.55	1.90	-0.23	1.47	-0.66	2.00	-0.13	2.23	0.09	2.13
cu_xantphos_phbpy	1.53	-0.67	1.92	-0.29	1.45	-0.76	2.01	-0.19	2.24	0.04	2.20
cu_xantphos_dmbpy	1.85	-0.45	2.22	-0.08	1.74	-0.56	2.33	0.03	2.59	0.29	2.30
cu_xantphos_44dtfmbpy	1.16	-0.80	1.58	-0.38	1.17	-0.79	1.70	-0.27	1.93	-0.03	1.96
cu_xantphos_bpy	1.43	-0.68	1.83	-0.28	1.37	-0.74	1.94	-0.17	2.14	0.03	2.11
cu_xantphos_etbpy	1.76	-0.51	2.14	-0.14	1.58	-0.70	2.13	-0.14	2.36	0.08	2.28
cu_xantphos_mbpy	1.74	-0.53	2.13	-0.14	1.56	-0.70	2.13	-0.14	2.34	0.07	2.27
cu2_cl2_dpmb2	2.20	-0.15	2.29	-0.07	1.40	-0.95	2.09	-0.26	2.59	0.23	2.35
cu2_cl2_dpmt2	1.89	-0.39	1.96	-0.32	1.75	-0.53	2.04	-0.24	3.09	0.80	2.28
cu2_cl2_dppb2	1.36	-0.96	1.81	-0.51	1.37	-0.96	1.89	-0.43	2.73	0.40	2.33
cu2_cl2_pnme22	1.55	-0.90	2.13	-0.32	1.34	-1.11	2.24	-0.22	2.68	0.23	2.45
cu2_br2_dpmb2	1.41	-1.01	1.84	-0.58	1.44	-0.99	1.97	-0.46	2.69	0.27	2.43
cu2_br2_dpmt2	1.98	-0.31	2.54	0.25	2.11	-0.18	2.81	0.52	3.04	0.75	2.29
cu2_br2_dppb2	1.41	-0.98	1.84	-0.55	1.46	-0.92	1.94	-0.44	2.81	0.43	2.38
cu2_br2_pnme22	1.75	-0.78	2.44	-0.09	1.58	-0.95	2.40	-0.13	3.08	0.55	2.53
cu2_i2_dpmb2	1.96	-0.53	1.96	-0.53	2.08	-0.41	2.11	-0.38	2.89	0.40	2.49
cu2_i2_dpmt2	2.05	-0.48	2.86	0.33	1.89	-0.64	2.67	0.14	3.27	0.74	2.53
cu2_i2_dppb2	1.83	-0.64	1.92	-0.55	1.79	-0.68	1.99	-0.48	2.71	0.24	2.47
cu2_i2_pnme22	1.82	-0.86	2.65	-0.02	1.68	-0.99	2.69	0.01	3.24	0.57	2.67

cu2_i2_pnpy22	2.13	-0.54	2.61	-0.06	2.07	-0.59	2.75	0.09	3.28	0.61	2.67
cu2_i2_phen2	0.45	-1.35	0.89	-0.92	1.12	-0.68	0.95	-0.85	1.30	-0.50	1.80
cu2_i2_dmp2	1.37	-0.48	1.85	-0.01	1.41	-0.44	1.90	0.04	2.22	0.36	1.86
cu2_i2_dppt12	2.06	-0.48	2.75	0.20	1.69	-0.86	2.50	-0.04	3.19	0.64	2.55
cu2_i2_dppt22	1.46	-1.11	2.02	-0.54	1.41	-1.16	1.95	-0.62	2.64	0.07	2.57
cu2_scn2_dppb2	1.36	-0.81	1.93	-0.24	1.35	-0.82	1.99	-0.18	2.33	0.16	2.17
cu2_scn2_pop2	2.12	-0.65	2.93	0.17	2.09	-0.68	2.90	0.13	3.35	0.58	2.77
cu_br_dmpzpp	1.44	-0.85	1.90	-0.39	1.33	-0.97	2.01	-0.29	2.33	0.04	2.29
cu_br_tpypo	1.34	-0.66	1.79	-0.21	1.20	-0.80	1.88	-0.12	2.20	0.20	2.00
cu_br_ttpp	1.27	-1.10	1.79	-0.58	1.21	-1.16	1.88	-0.49	2.18	-0.19	2.37
cu_cl_tpypo	1.23	-0.69	1.67	-0.25	1.11	-0.81	1.77	-0.15	2.09	0.17	1.92
cu_cl_ttpp	1.23	-1.11	1.74	-0.60	1.15	-1.19	1.83	-0.51	2.31	-0.03	2.34
cu_i_dmpzpp	1.72	-0.62	1.97	-0.36	1.52	-0.82	2.09	-0.25	2.44	0.10	2.34
cu_i_tpyaso	1.51	-0.55	2.07	0.00	1.34	-0.73	2.15	0.08	2.50	0.43	2.07
cu_i_tpym	1.60	-0.66	2.16	-0.09	1.43	-0.83	2.23	-0.02	2.60	0.35	2.25
cu_i_tpypo	1.38	-0.68	1.92	-0.15	1.22	-0.84	2.00	-0.07	2.35	0.28	2.07
cu_i_tpyps	1.38	-0.70	1.91	-0.17	1.21	-0.87	2.00	-0.09	2.33	0.24	2.08
cu_i_tpypse	1.38	-0.56	1.91	-0.03	1.21	-0.73	1.99	0.06	2.32	0.38	1.94
cu_i_ttpp	1.34	-1.04	1.89	-0.49	1.29	-1.09	1.98	-0.40	2.30	-0.08	2.38
cu_i_ttppo	1.04	-0.96	1.54	-0.46	0.89	-1.10	1.65	-0.35	2.03	0.03	2.00
cu_cn_ttpp	1.28	-1.06	1.81	-0.53	1.21	-1.13	1.91	-0.43	2.32	-0.02	2.34
cu_scn_ttpp	1.38	-1.01	1.89	-0.49	1.42	-0.96	1.97	-0.41	2.28	-0.11	2.38
cu_sph_dmpzpp	1.54	-0.75	2.44	0.14	1.30	-1.00	2.36	0.07	2.80	0.50	2.30
cu_ccph_ttpp	1.20	-0.86	1.70	-0.36	1.15	-0.91	1.79	-0.27	2.34	0.28	2.06
cu_ccnph_ttpp	1.28	-0.43	1.89	0.18	1.20	-0.51	1.95	0.23	2.26	0.54	1.72
cu_ccpyr_ttpp	1.23	-0.93	1.73	-0.43	1.16	-1.00	1.87	-0.29	2.33	0.17	2.16

Table S16. Predicted $S_1 \rightarrow S_0$ fluorescence energies with the QM/MM models.

	QM/MM										
	**PBE0**		**LRC-ωPBEh**		**B3LYP**		**CAM-B3LYP**		**ωB97X**		**EXP**
	E [eV]	Error [eV]	E [eV]	Error [eV]	E [eV]	Error [eV]	E [eV]	Error [eV]	E [eV]	Error [eV]	E [eV]
cu_pop_pypz	1.94	-0.59	2.43	-0.10	1.82	-0.71	2.53	0.00	2.85	0.32	2.53
cu_pop_pympz	2.16	-0.51	2.67	0.00	2.05	-0.62	2.77	0.10	3.10	0.43	2.67
cu_pop_pytfmpz	2.03	-0.49	2.46	-0.06	1.88	-0.65	2.55	0.03	2.88	0.36	2.52
cu_pop_bpy	1.77	-0.36	2.11	-0.03	1.57	-0.56	2.23	0.09	2.49	0.36	2.13
cu_pop_mbpy	1.86	-0.33	2.21	0.02	1.73	-0.45	2.32	0.13	2.58	0.40	2.19
cu_pop_dmbpy	2.11	-0.21	2.43	0.11	1.97	-0.35	2.52	0.20	2.78	0.46	2.32
cu_pop_etbpy	1.95	-0.27	2.31	0.08	1.81	-0.41	2.39	0.16	2.65	0.43	2.23
cu_pop_phbpy	1.67	-0.48	2.04	-0.12	1.54	-0.61	2.14	-0.02	2.40	0.25	2.15
cu_pop_tfmbpy	1.63	-0.52	2.02	-0.14	1.50	-0.65	2.10	-0.06	2.35	0.20	2.16
cu_pop_55dtfmbpy	1.52	-0.40	1.95	0.03	1.38	-0.53	2.02	0.11	2.33	0.41	1.91
cu_pop_44dtfmbpy	1.55	-0.31	2.02	0.16	1.45	-0.42	2.05	0.18	2.33	0.46	1.87
cu_xantphos_tfmbpy	1.67	-0.46	2.01	-0.12	1.56	-0.58	2.10	-0.03	2.34	0.21	2.13
cu_xantphos_phbpy	1.99	-0.22	2.04	-0.17	1.86	-0.34	2.14	-0.07	2.42	0.22	2.20
cu_xantphos_dmbpy	1.97	-0.33	2.33	0.03	1.84	-0.46	2.43	0.13	2.68	0.38	2.30
cu_xantphos_44dtfmbpy	1.93	-0.03	1.99	0.03	1.80	-0.17	2.42	0.46	2.34	0.38	1.96
cu_xantphos_bpy	1.48	-0.63	1.88	-0.24	1.36	-0.75	1.98	-0.14	2.25	0.14	2.11
cu_xantphos_etbpy	1.91	-0.37	2.28	0.00	1.78	-0.50	2.36	0.09	2.62	0.35	2.28
cu_xantphos_mbpy	1.88	-0.39	2.27	0.00	1.76	-0.51	2.34	0.08	2.61	0.34	2.27
cu2_cl2_dpmb2	2.20	-0.15	2.75	0.39	2.09	-0.26	2.69	0.34	2.91	0.56	2.35

cu2_cl2_dpmt2	2.27	-0.01	2.85	0.57	2.17	-0.11	2.92	0.63	3.22	0.94	2.28
cu2_cl2_dppb2	2.12	-0.20	2.57	0.25	1.99	-0.34	2.63	0.30	2.90	0.57	2.33
cu2_cl2_pnme22	2.10	-0.35	2.67	0.21	1.95	-0.50	2.77	0.32	3.17	0.72	2.45
cu2_br2_dpmb2	2.27	-0.16	2.84	0.42	2.16	-0.27	2.74	0.32	2.99	0.56	2.43
cu2_br2_dpmt2	2.29	0.01	2.88	0.60	2.18	-0.11	2.93	0.65	3.25	0.97	2.29
cu2_br2_dppb2	2.20	-0.18	2.66	0.27	2.08	-0.31	2.72	0.34	3.00	0.62	2.38
cu2_br2_pnme22	2.23	-0.30	2.74	0.21	2.05	-0.48	2.85	0.32	3.24	0.71	2.53
cu2_i2_dpmb2	2.39	-0.10	2.85	0.36	2.30	-0.19	2.91	0.42	3.14	0.65	2.49
cu2_i2_dpmt2	2.39	-0.14	2.91	0.38	2.29	-0.24	3.00	0.47	3.27	0.74	2.53
cu2_i2_dppb2	2.29	-0.18	2.83	0.36	2.15	-0.32	2.79	0.32	3.05	0.58	2.47
cu2_i2_pnme22	2.32	-0.35	2.89	0.21	2.16	-0.51	2.98	0.30	3.36	0.68	2.67
cu2_i2_pnpy22	2.36	-0.31	2.90	0.24	2.20	-0.47	2.98	0.31	3.36	0.69	2.67
cu2_i2_phen2	1.26	-0.54	1.68	-0.12	1.11	-0.69	1.78	-0.02	2.05	0.25	1.80
cu2_i2_dmp2	1.45	-0.41	1.84	-0.02	1.28	-0.58	1.93	0.07	2.20	0.34	1.86
cu2_i2_dppt12	2.35	-0.19	3.01	0.46	2.21	-0.34	3.05	0.50	3.43	0.89	2.55
cu2_i2_dppt22	1.86	-0.71	2.36	-0.21	1.71	-0.86	2.41	-0.16	2.69	0.13	2.57
cu2_scn2_dppb2	1.86	-0.31	2.34	0.17	1.76	-0.41	2.43	0.25	2.72	0.55	2.17
cu2_scn2_pop2	2.57	-0.20	3.51	0.74	2.36	-0.41	3.33	0.56	3.66	0.89	2.77
cu_br_dmpzpp	2.04	-0.25	2.62	0.33	1.90	-0.39	2.69	0.40	3.09	0.80	2.29
cu_br_tpypo	1.34	-0.66	1.80	-0.20	1.20	-0.80	1.89	-0.11	2.20	0.20	2.00
cu_br_ttpp	1.90	-0.47	2.42	0.05	1.79	-0.58	2.48	0.11	2.81	0.44	2.37
cu_cl_tpypo	1.21	-0.71	1.64	-0.28	1.09	-0.84	1.74	-0.18	2.05	0.13	1.92
cu_cl_ttpp	1.88	-0.46	2.39	0.05	1.77	-0.57	2.45	0.11	2.78	0.44	2.34
cu_i_dmpzpp	1.93	-0.41	2.53	0.19	1.73	-0.61	2.57	0.23	2.93	0.59	2.34
cu_i_tpyaso	1.51	-0.55	2.07	0.00	1.33	-0.74	2.14	0.07	2.48	0.42	2.07
cu_i_tpym	1.63	-0.62	2.20	-0.06	1.44	-0.81	2.26	0.00	2.61	0.36	2.25
cu_i_tpypo	1.38	-0.68	1.92	-0.15	1.20	-0.86	1.99	-0.08	2.32	0.26	2.07
cu_i_tpyps	1.41	-0.67	1.97	-0.11	1.21	-0.87	2.04	-0.04	2.37	0.28	2.08
cu_i_tpypse	1.38	-0.56	1.91	-0.03	1.20	-0.74	1.98	0.04	2.30	0.36	1.94
cu_i_ttpp	1.89	-0.49	2.46	0.08	1.75	-0.63	2.51	0.13	2.83	0.45	2.38
cu_i_ttppo	1.60	-0.39	2.20	0.20	1.99	-0.01	2.26	0.26	2.72	0.72	2.00
cu_cn_ttpp	1.99	-0.35	2.51	0.17	1.89	-0.45	2.58	0.24	2.90	0.56	2.34
cu_scn_ttpp	2.11	-0.27	2.57	0.19	1.94	-0.44	2.64	0.26	2.94	0.56	2.38
cu_sph_dmpzpp	1.71	-0.59	2.63	0.33	1.49	-0.81	2.63	0.33	3.09	0.80	2.30
cu_ccph_ttpp	1.85	-0.21	2.34	0.28	1.74	-0.32	2.40	0.34	2.71	0.65	2.06
cu_ccnph_ttpp	1.93	0.21	2.43	0.72	1.82	0.10	2.48	0.76	2.81	1.09	1.72
cu_ccpyr_ttpp	1.95	-0.22	2.41	0.25	1.85	-0.31	2.47	0.31	2.81	0.65	2.16

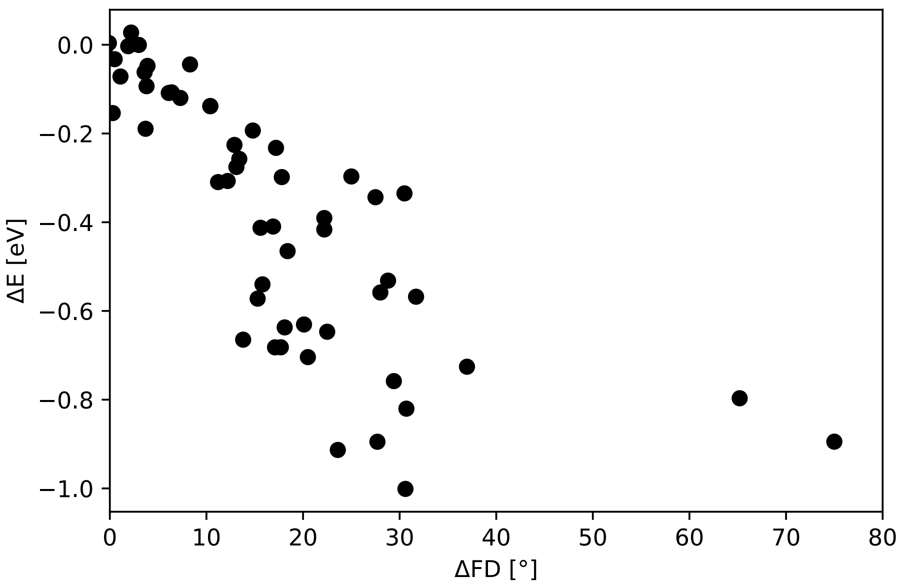

Figure S3. Change in predicted $S_1 \rightarrow S_0$ transition energy vs. change in flattening degree between the isolated and QM/MM models with the LRC-ωPBEh functional.

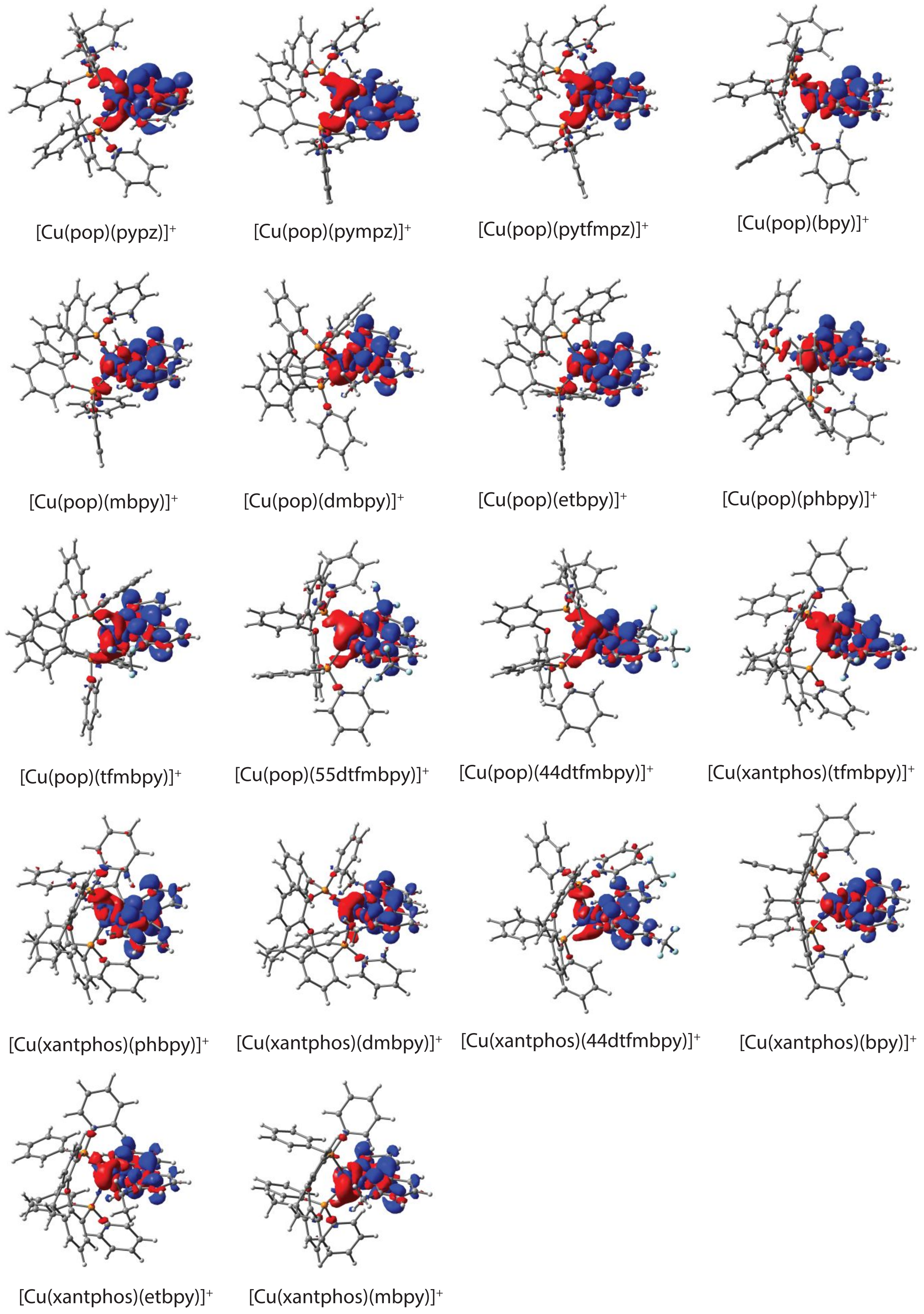

Figure S4. Electron density difference plots on a 0.002 a.u. isosurface for $S_1 \rightarrow S_0$ fluorescence emission for the $[Cu(NN)(PP)]^+$ complexes with QM/MM models and PBE0 functional. Red (blue) regions imply increasing (decreasing) electron density during the transition.

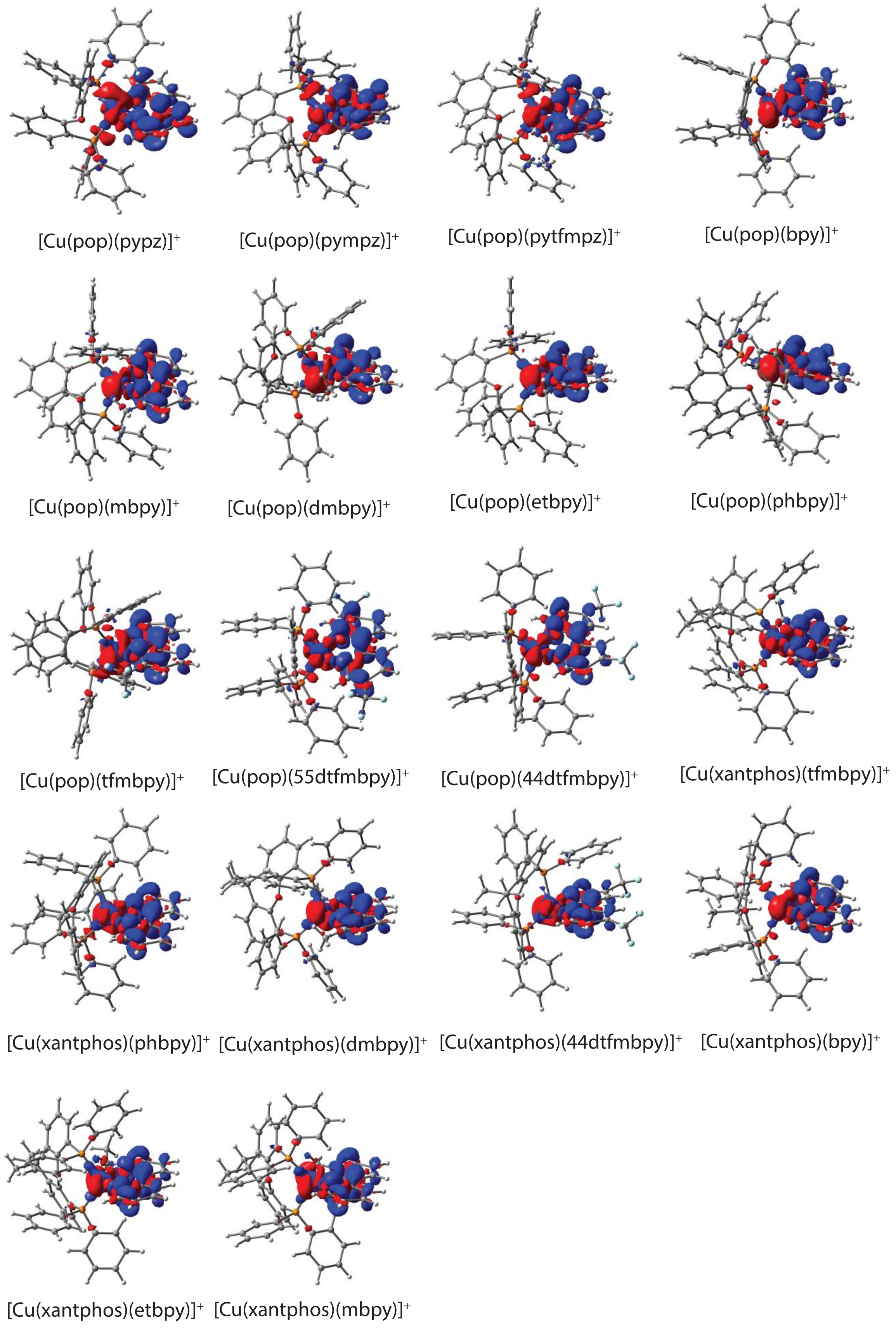

Figure S5. Electron density difference plots on a 0.002 a.u. isosurface for $S_1 \rightarrow S_0$ fluorescence emission for the $[Cu(NN)(PP)]^+$ complexes with QM/MM models and LRC-ωPBEh functional. Red (blue) regions imply increasing (decreasing) electron density during the transition.

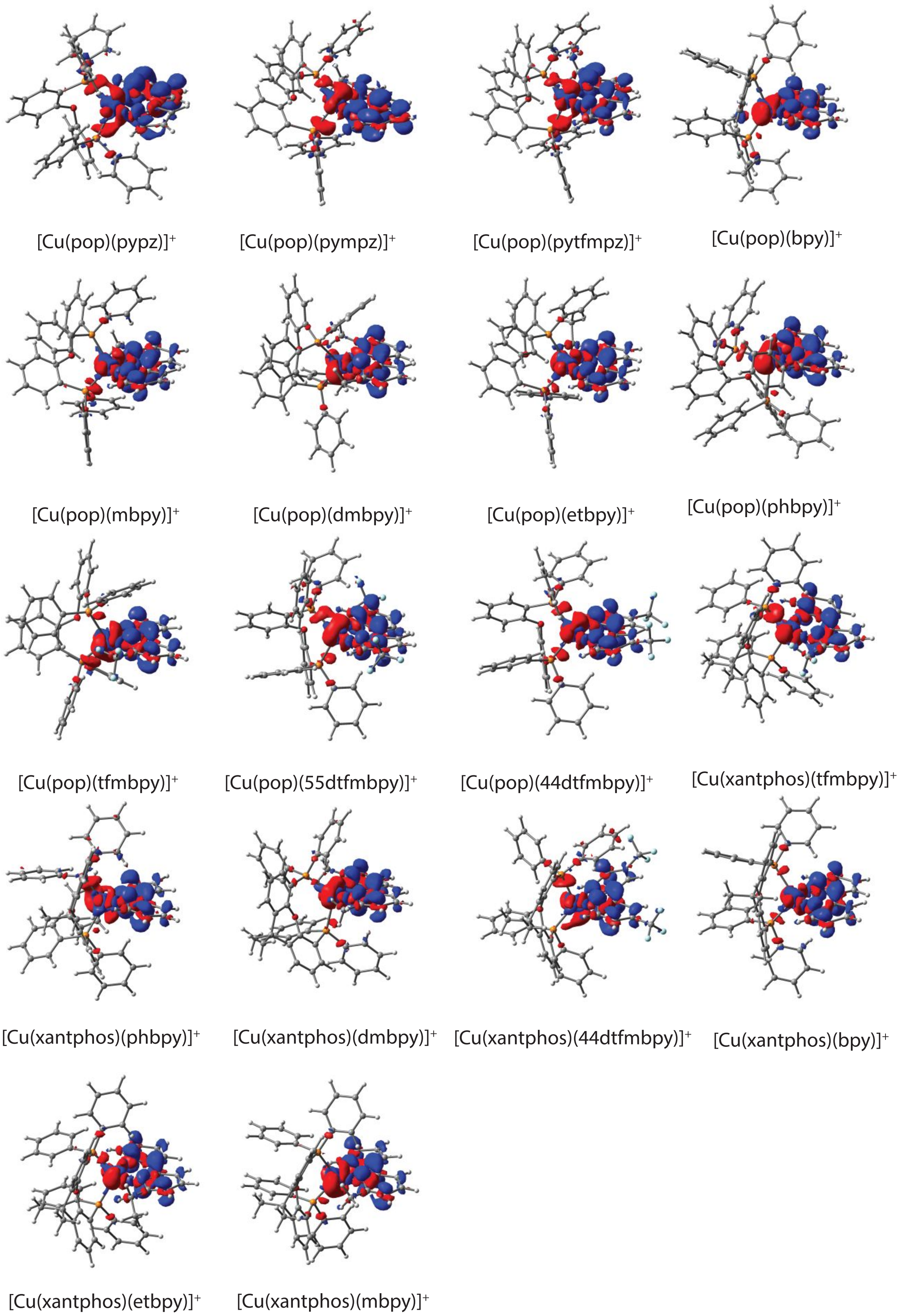

Figure S6. Electron density difference plots on a 0.002 a.u. isosurface for $S_1 \rightarrow S_0$ fluorescence emission for the $[Cu(NN)(PP)]^+$ complexes with QM/MM models and B3LYP functional. Red (blue) regions imply increasing (decreasing) electron density during the transition.

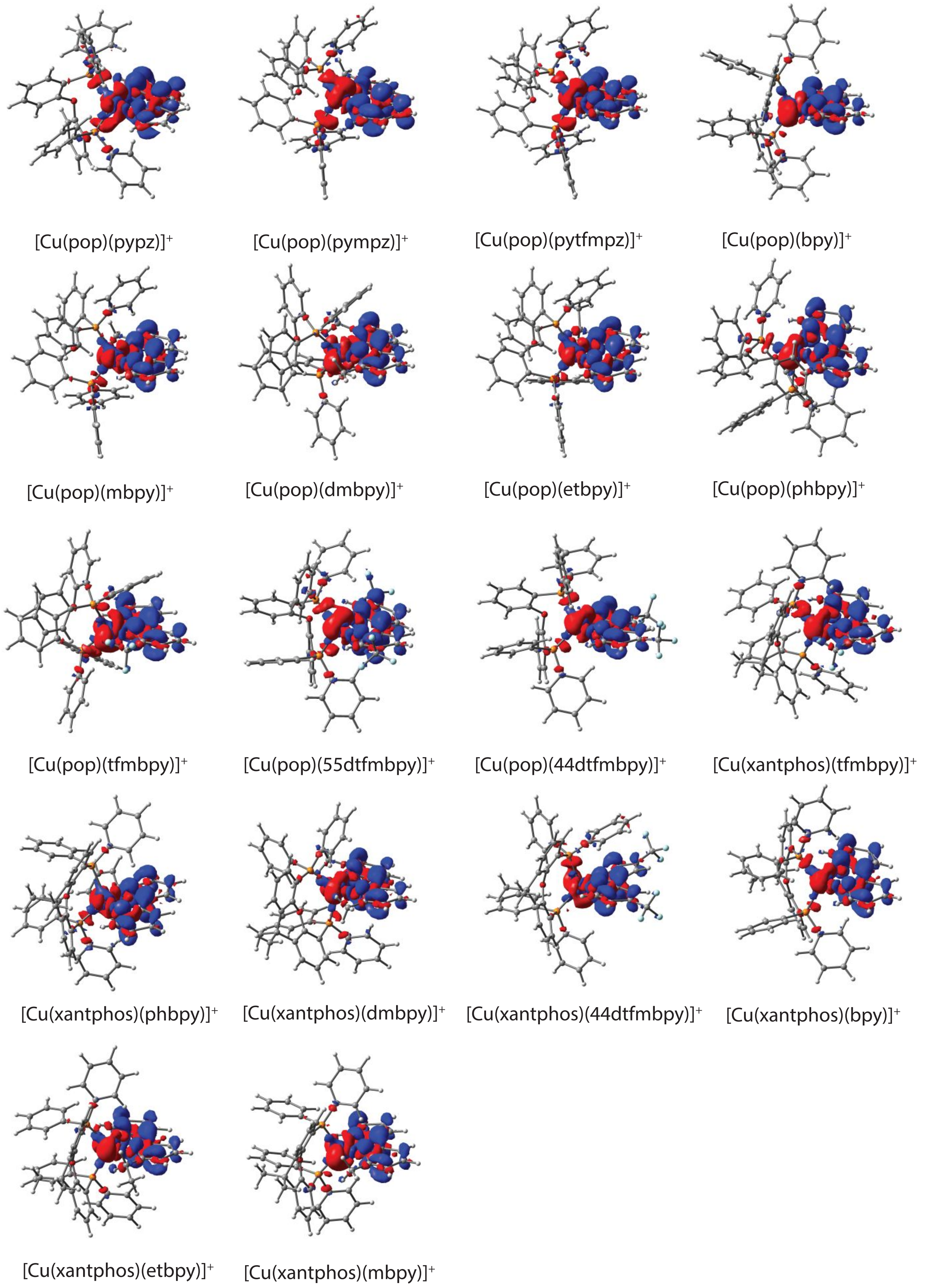

Figure S7. Electron density difference plots on a 0.002 a.u. isosurface for $S_1 \rightarrow S_0$ fluorescence emission for the $[Cu(NN)(PP)]^+$ complexes with QM/MM models and CAM-B3LYP functional. Red (blue) regions imply increasing (decreasing) electron density during the transition.

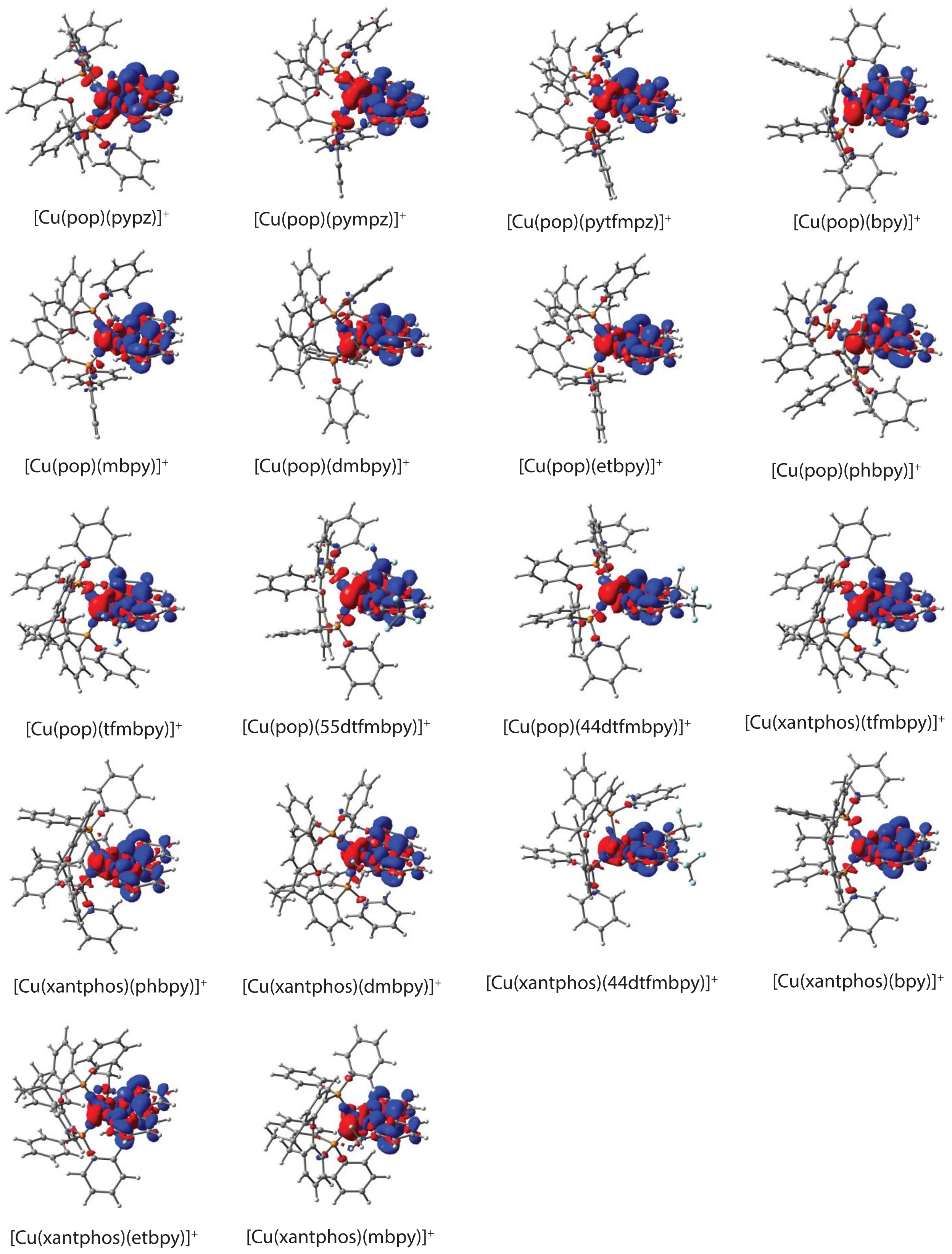

Figure S8. Electron density difference plots on a 0.002 a.u. isosurface for $S_1 \rightarrow S_0$ fluorescence emission for the $[Cu(NN)(PP)]^+$ complexes with QM/MM models and ωB97X functional. Red (blue) regions imply increasing (decreasing) electron density during the transition.

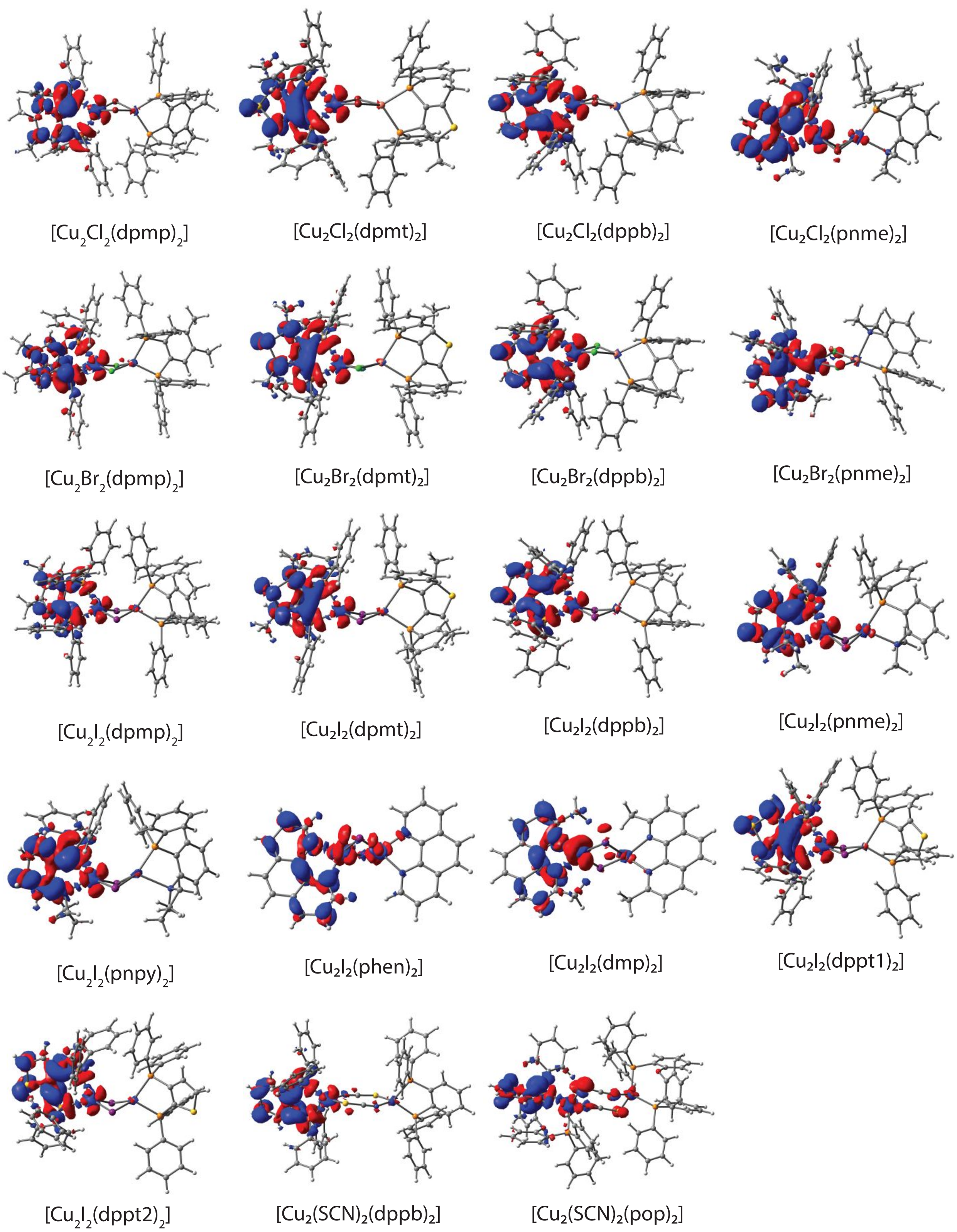

Figure S9. Electron density difference plots on a 0.002 a.u. isosurface for $S_1 \rightarrow S_0$ fluorescence emission for the [$Cu_2X_2(L^2)_2$] complexes with QM/MM models and PBE0 functional. Red (blue) regions imply increasing (decreasing) electron density during the transition.

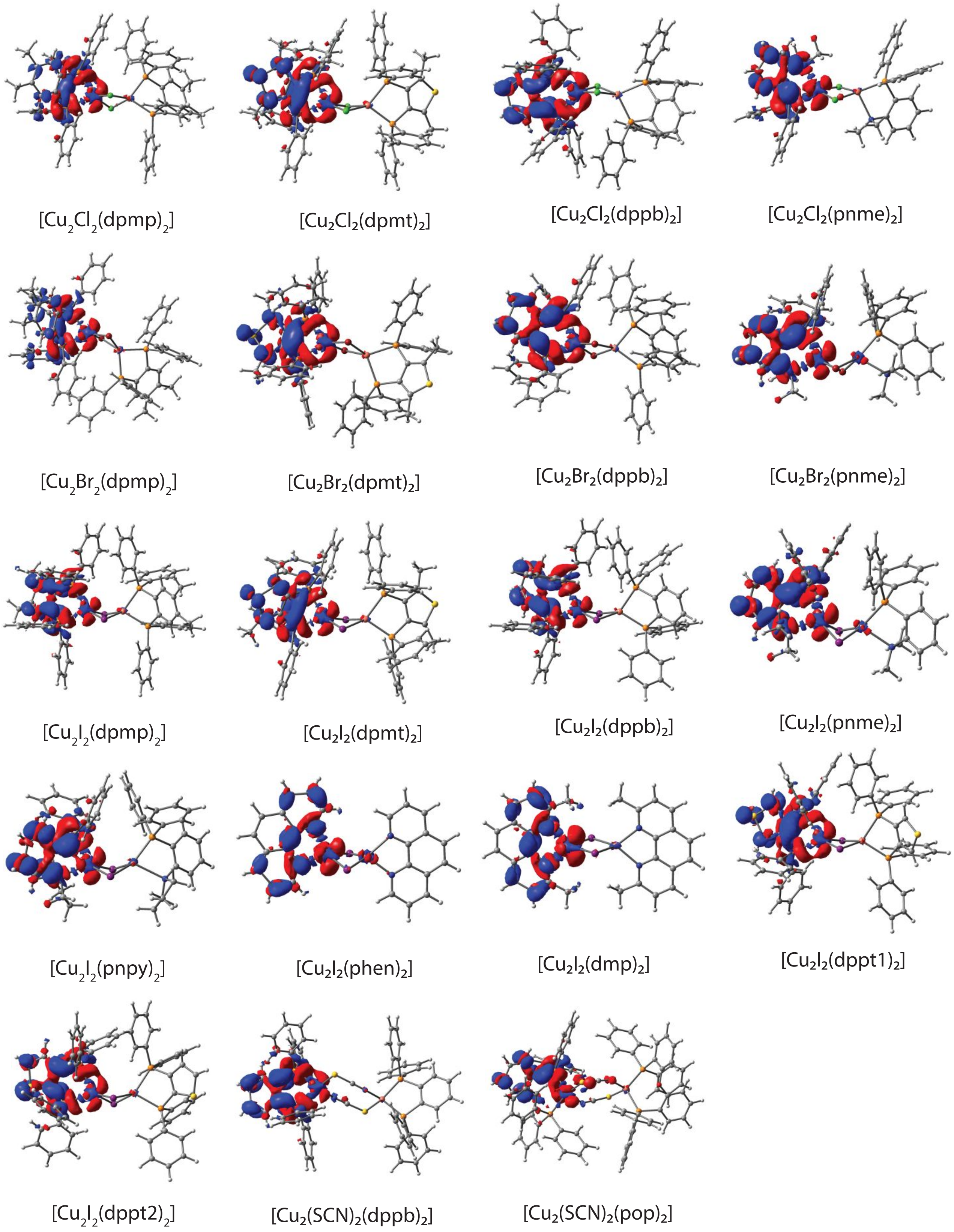

Figure S10. Electron density difference plots on a 0.002 a.u. isosurface for $S_1 \rightarrow S_0$ fluorescence emission for the $[Cu_2X_2(L^2)_2]$ complexes with QM/MM models and LRC-ωPBEh functional. Red (blue) regions imply increasing (decreasing) electron density during the transition.

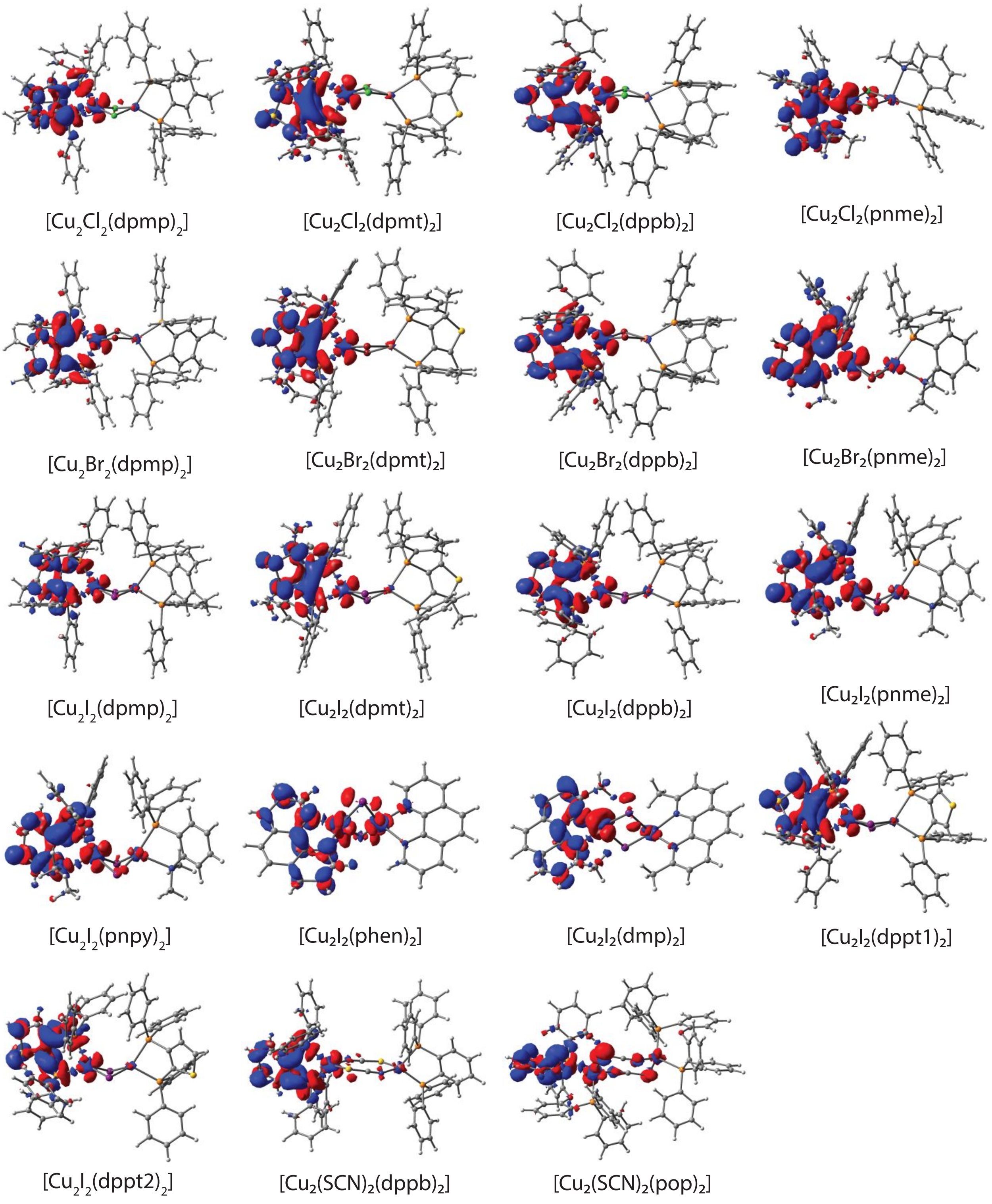

Figure S11. Electron density difference plots on a 0.002 a.u. isosurface for $S_1 \rightarrow S_0$ fluorescence emission for the [$Cu_2X_2(L^2)_2$] complexes with QM/MM models and B3LYP functional. Red (blue) regions imply increasing (decreasing) electron density during the transition.

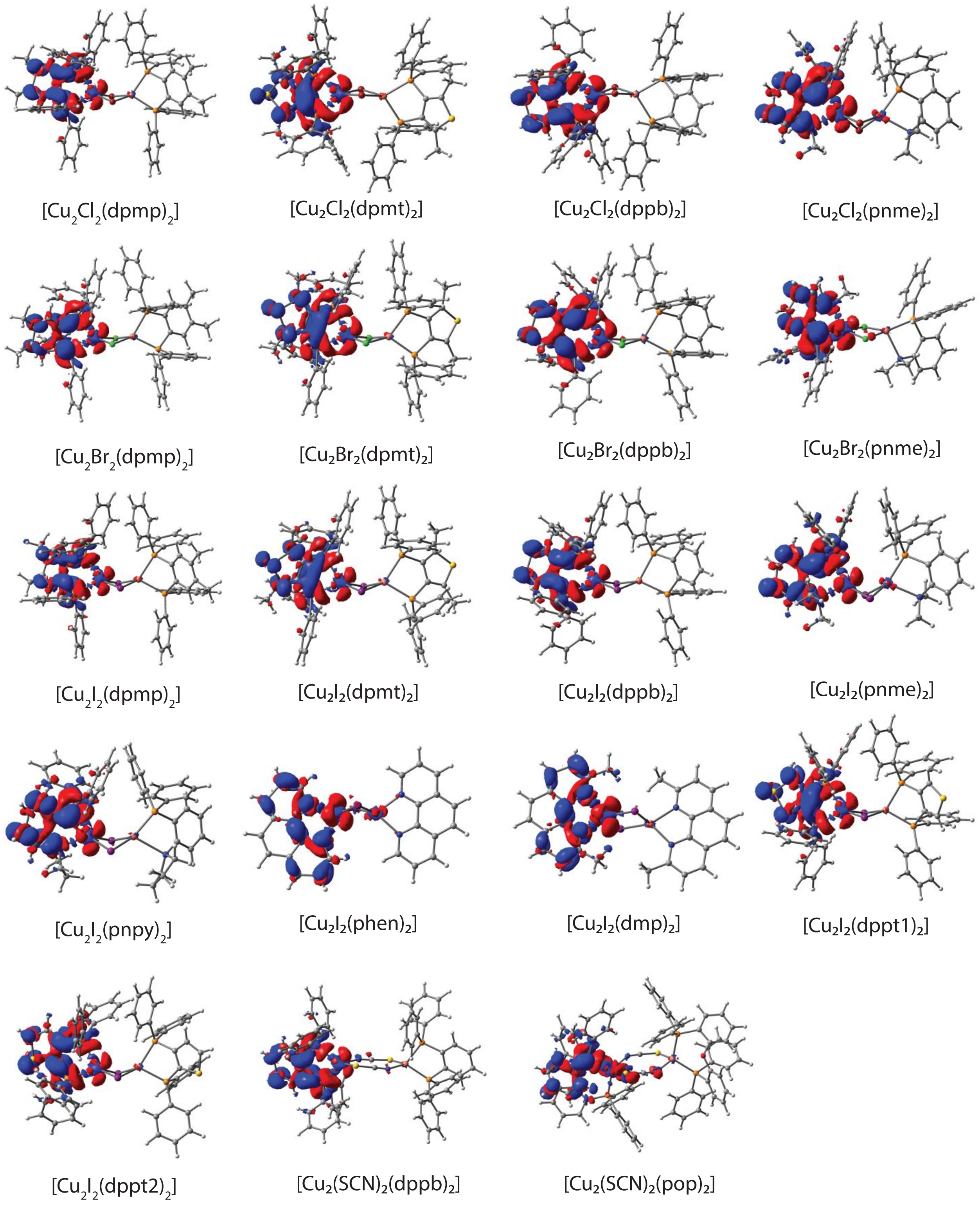

Figure S12. Electron density difference plots on a 0.002 a.u. isosurface for $S_1 \rightarrow S_0$ fluorescence emission for the $[Cu_2X_2(L^2)_2]$ complexes with QM/MM models and CAM-B3LYP functional. Red (blue) regions imply increasing (decreasing) electron density during the transition.

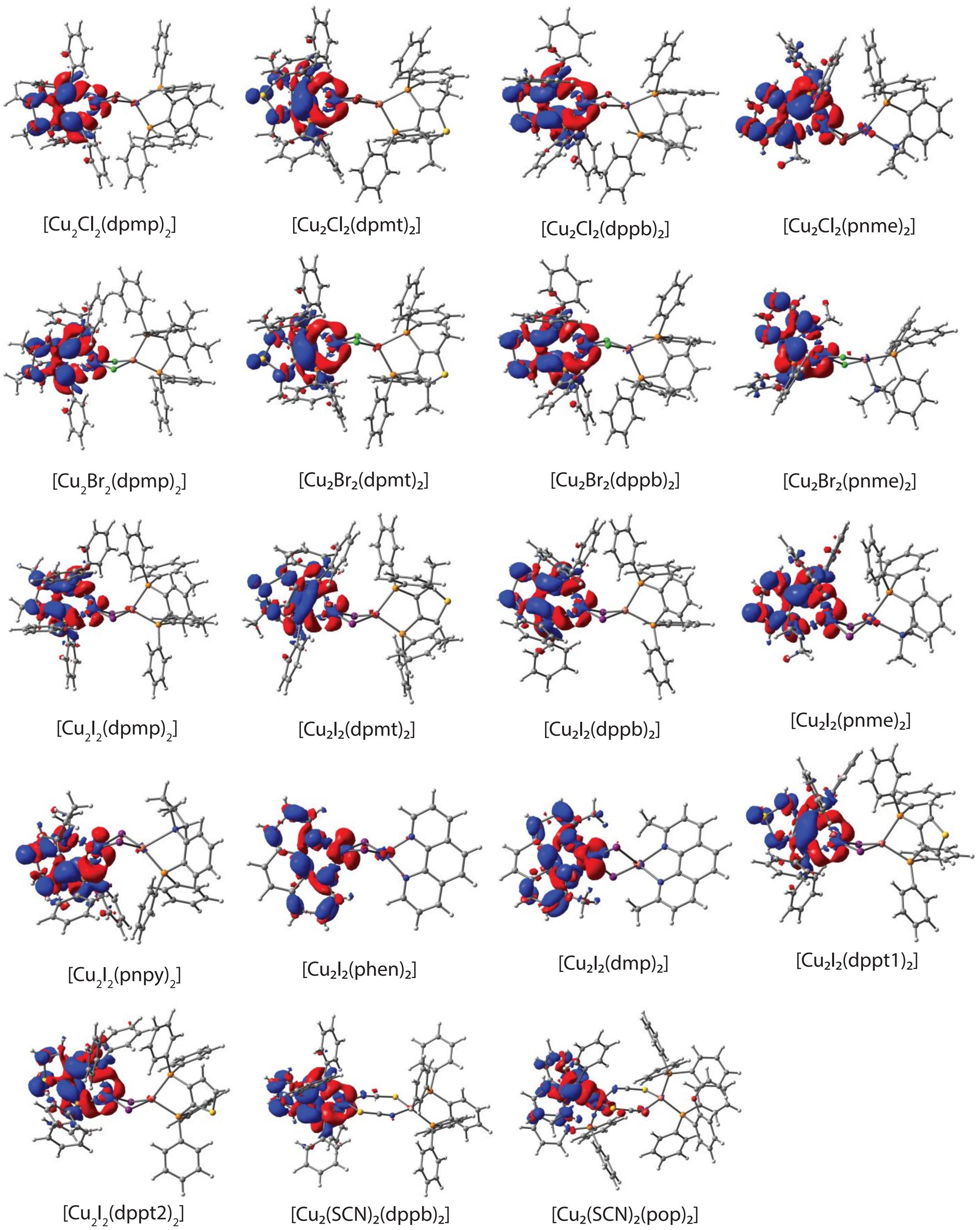

Figure S13. Electron density difference plots on a 0.002 a.u. isosurface for $S_1 \rightarrow S_0$ fluorescence emission for the $[Cu_2X_2(L^2)_2]$ complexes with QM/MM models and ωB97X functional. Red (blue) regions imply increasing (decreasing) electron density during the transition.

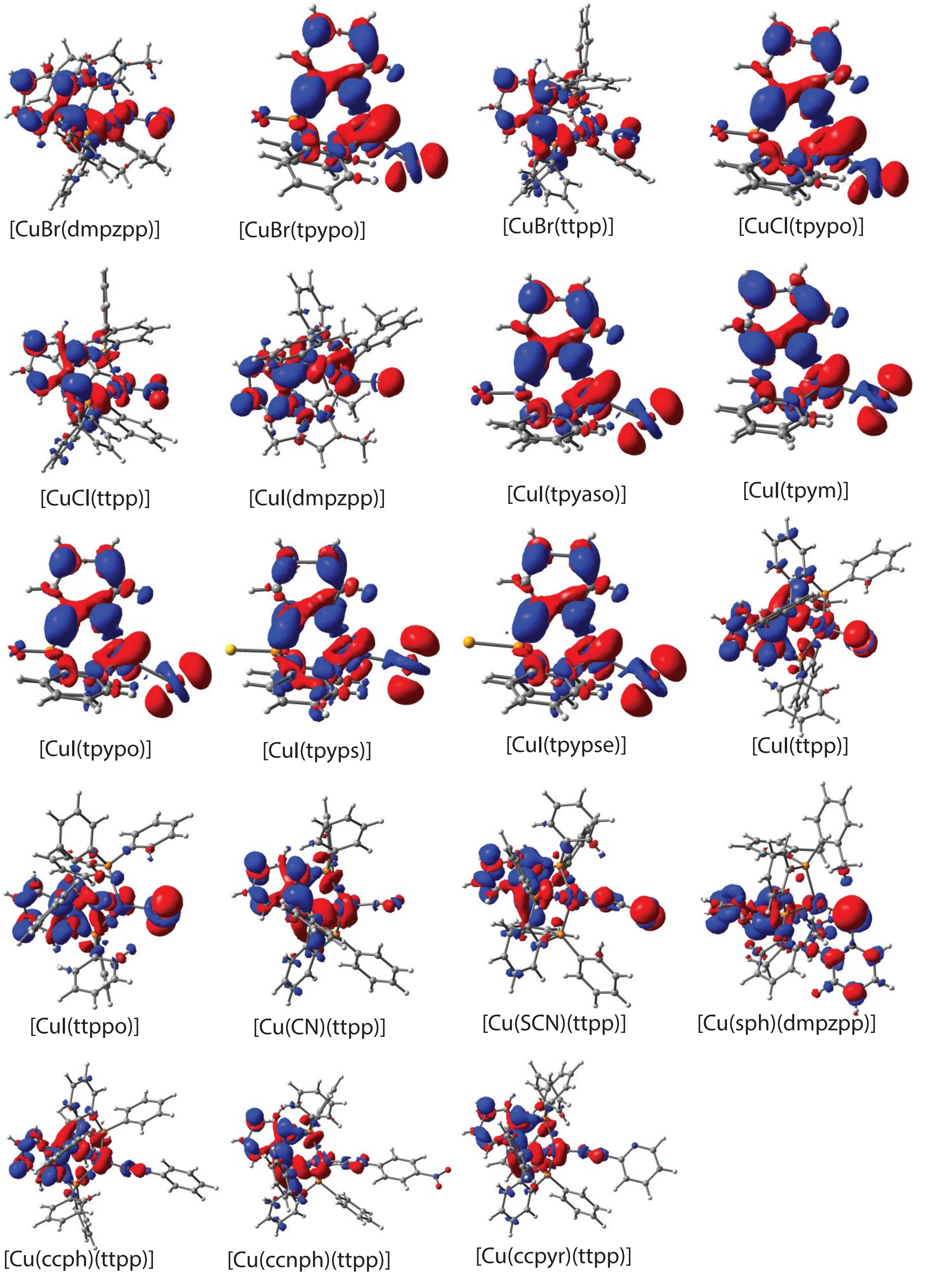

Figure S14. Electron density difference plots on a 0.002 a.u. isosurface for $S_1 \rightarrow S_0$ fluorescence emission for the [CuX(L^3)] complexes with QM/MM models and PBE0 functional. Red (blue) regions imply increasing (decreasing) electron density during the transition.

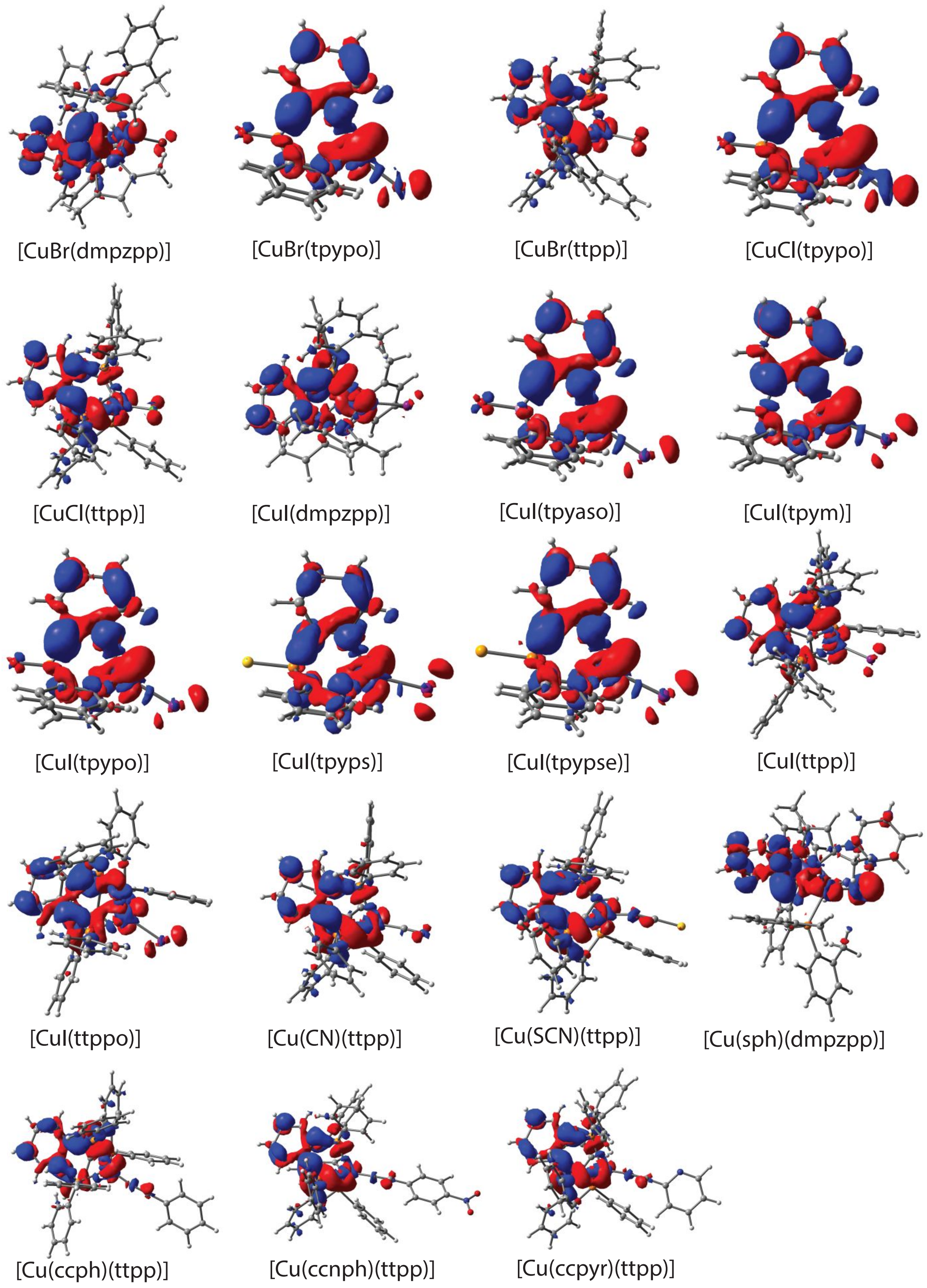

Figure S15. Electron density difference plots on a 0.002 a.u. isosurface for $S_1 \rightarrow S_0$ fluorescence emission for the [CuX(L^3)] complexes with QM/MM models and LRC-ωPBEh functional. Red (blue) regions imply increasing (decreasing) electron density during the transition.

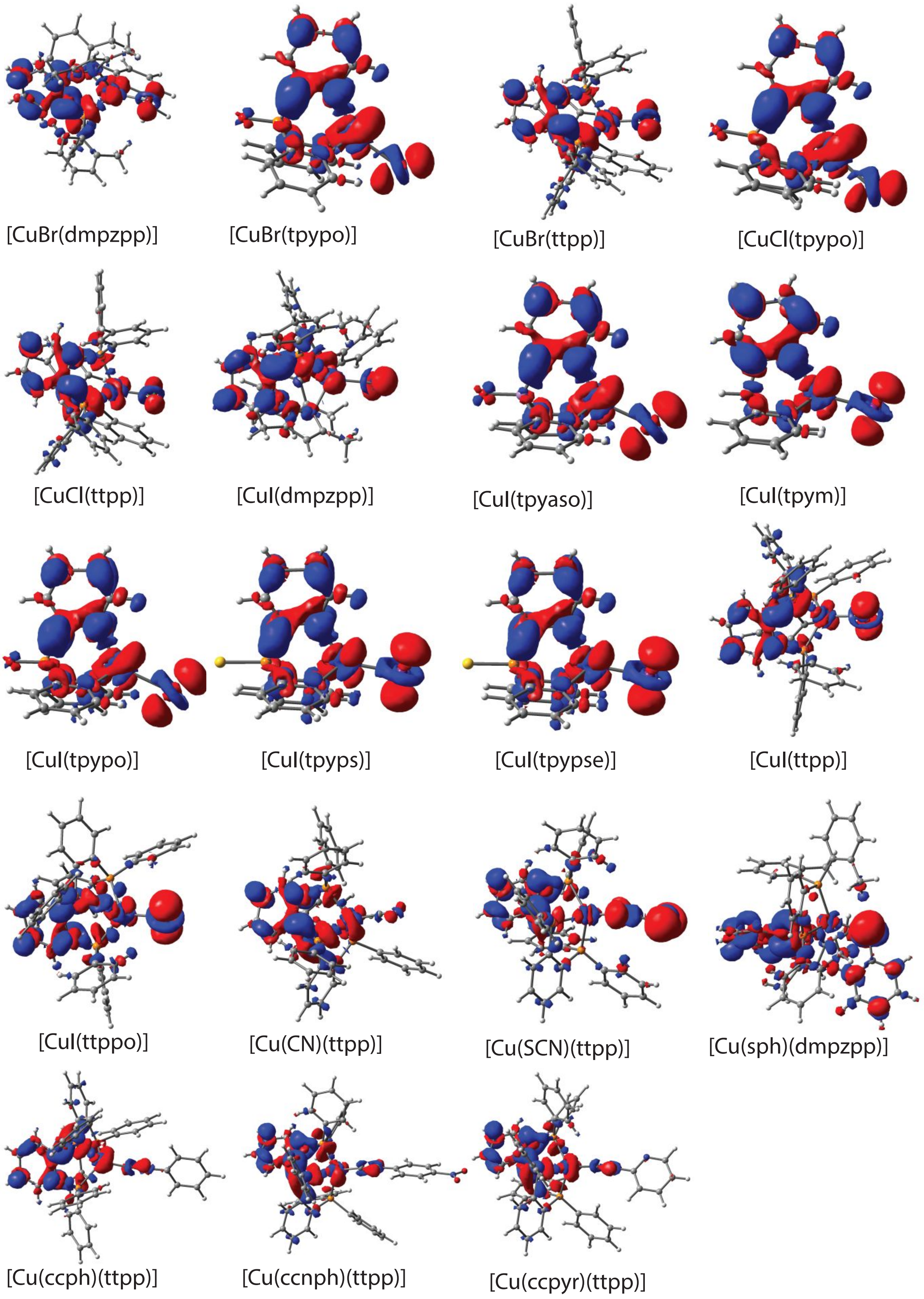

Figure S16. Electron density difference plots on a 0.002 a.u. isosurface for $S_1 \rightarrow S_0$ fluorescence emission for the $[CuX(L^3)]$ complexes with QM/MM models and B3LYP functional. Red (blue) regions imply increasing (decreasing) electron density during the transition.

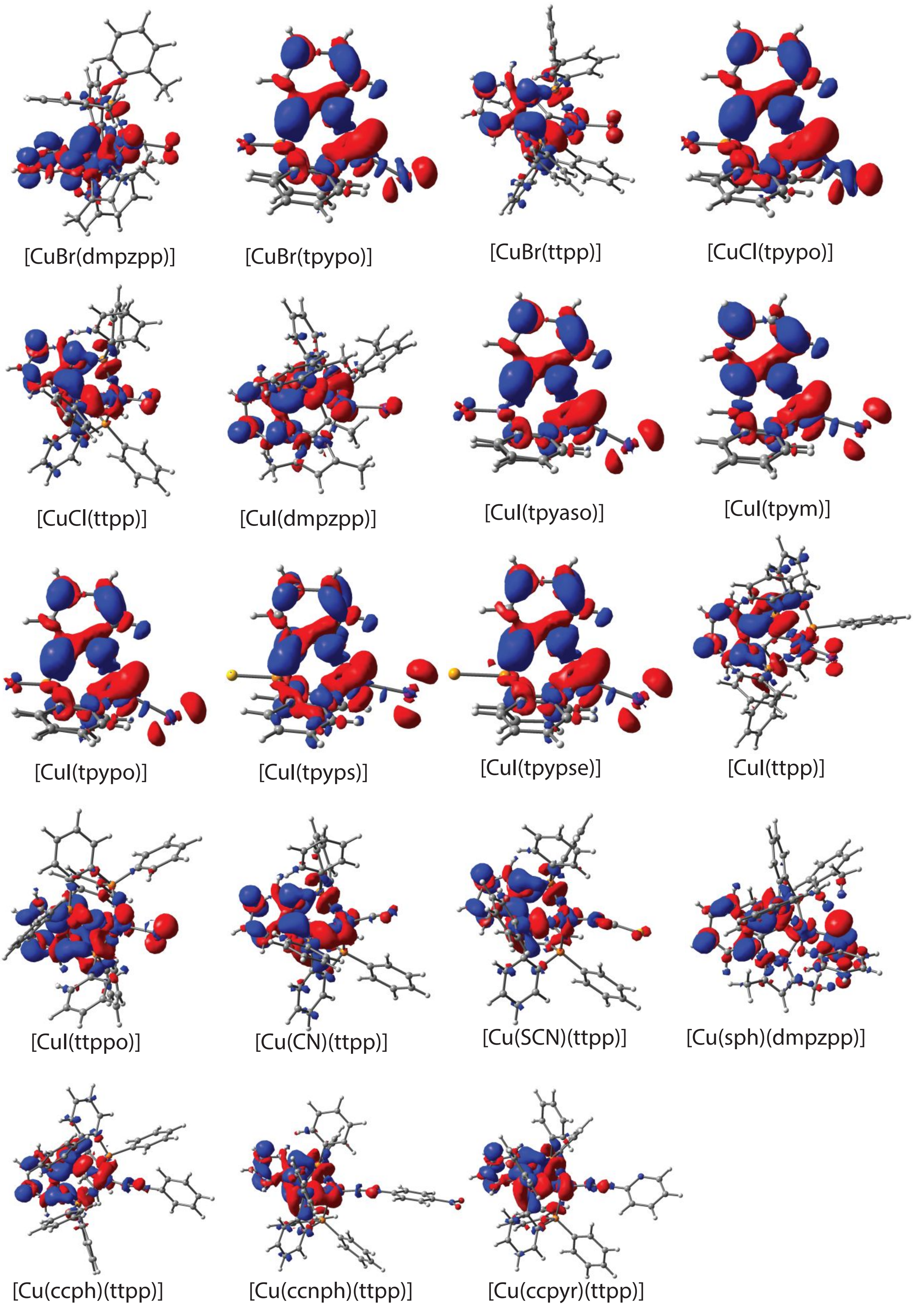

Figure S17. Electron density difference plots on a 0.002 a.u. isosurface for $S_1 \rightarrow S_0$ fluorescence emission for the [CuX(L^3)] complexes with QM/MM models and CAM-B3LYP functional. Red (blue) regions imply increasing (decreasing) electron density during the transition.

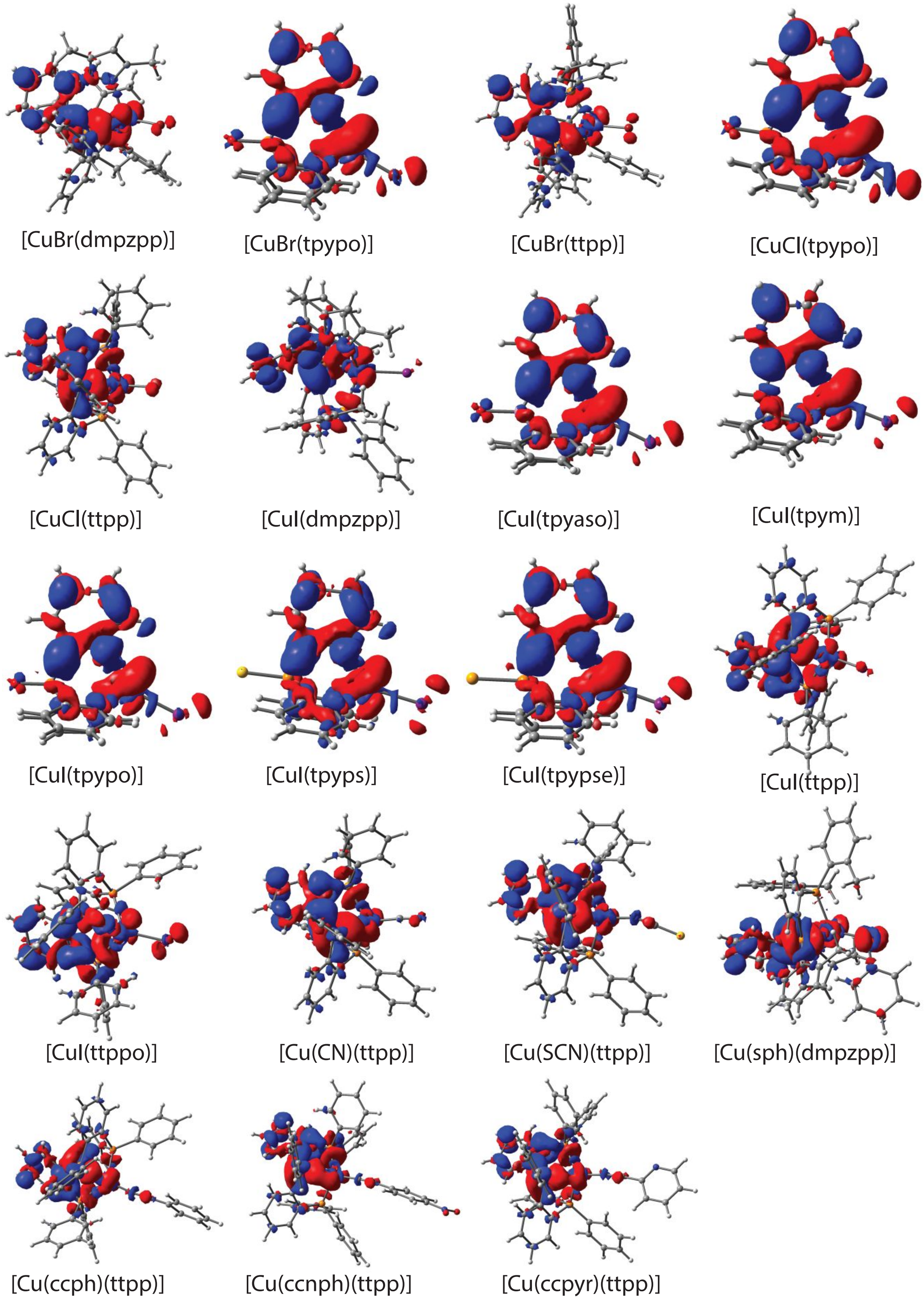

Figure S18. Electron density difference plots on a 0.002 a.u. isosurface for $S_1 \rightarrow S_0$ fluorescence emission for the [CuX(L^3)] complexes with QM/MM models and ωB97X functional. Red (blue) regions imply increasing (decreasing) electron density during the transition.

Table S17. $\Delta E(S_1\text{-}T_1)$ energy differences estimated as energy differences in $S_0 \rightarrow S_1$ and $S_0 \rightarrow T_1$ vertical excitations using the QM/MM models. Entries highlighted in orange exceed the 0.37 eV threshold.

	PBE0	LRC-ωPBEh	B3LYP	CAM-B3LYP	ωB97X
cu_pop_pypz	0.292	0.375	0.256	0.352	0.486
cu_pop_pympz	0.292	0.505	0.247	0.500	0.770
cu_pop_pytfmpz	0.304	0.403	0.267	0.348	0.509
cu_pop_bpy	0.273	0.218	0.242	0.224	0.358
cu_pop_mbpy	0.237	0.284	0.209	0.238	0.469
cu_pop_dmbpy	0.232	0.305	0.200	0.328	0.688
cu_pop_etbpy	0.246	0.252	0.219	0.277	0.574
cu_pop_phbpy	0.226	0.267	0.206	0.245	0.400
cu_pop_tfmbpy	0.282	0.346	0.253	0.309	0.350
cu_pop_55dtfmbpy	0.206	0.221	0.186	0.204	0.223
cu_pop_44dtfmbpy	0.334	0.319	0.330	0.312	0.310
cu_xantphos_tfmbpy	0.299	0.292	0.277	0.281	0.288
cu_xantphos_phbpy	0.255	0.273	0.226	0.280	0.483
cu_xantphos_dmbpy	0.199	0.218	0.171	0.214	0.764
cu_xantphos_44dtfmbpy	0.316	0.338	0.294	0.301	0.342
cu_xantphos_bpy	0.298	0.333	0.276	0.296	0.401
cu_xantphos_etbpy	0.287	0.240	0.268	0.273	0.423
cu_xantphos_mbpy	0.297	0.251	0.277	0.295	0.410
cu2_cl2_dpmb2	0.187	0.322	0.144	0.295	0.391
cu2_cl2_dpmt2	0.176	0.341	0.144	0.319	0.461
cu2_cl2_dppb2	0.138	0.294	0.103	0.245	0.360
cu2_cl2_pnme22	0.082	0.196	0.056	0.179	0.435
cu2_br2_dpmb2	0.179	0.324	0.139	0.296	0.400
cu2_br2_dpmt2	0.167	0.340	0.117	0.312	0.477
cu2_br2_dppb2	0.141	0.325	0.099	0.286	0.406
cu2_br2_pnme22	0.123	0.238	0.086	0.230	0.366
cu2_i2_dpmb2	0.152	0.360	0.100	0.330	0.465
cu2_i2_dpmt2	0.164	0.425	0.111	0.417	0.631
cu2_i2_dppb2	0.124	0.319	0.112	0.293	0.431
cu2_i2_pnme22	0.091	0.232	0.148	0.220	0.466
cu2_i2_pnpy22	0.117	0.268	0.076	0.276	0.523
cu2_i2_phen2	0.057	0.129	0.047	0.087	0.141
cu2_i2_dmp2	0.305	0.127	0.034	0.162	0.201
cu2_i2_dppt12	0.093	0.338	0.060	0.309	0.536
cu2_i2_dppt22	0.060	0.295	0.048	0.307	0.596
cu2_scn2_dppb2	0.076	0.265	0.056	0.230	0.323
cu2_scn2_pop2	0.109	0.634	0.043	0.614	0.878
cu_br_dmpzpp	0.126	0.266	0.090	0.231	0.354
cu_br_tpypo	0.105	0.143	0.098	0.128	0.139
cu_br_ttpp	0.104	0.213	0.075	0.189	0.274
cu_cl_tpypo	0.140	0.156	0.127	0.140	0.146
cu_cl_ttpp	0.120	0.217	0.093	0.197	0.277
cu_i_dmpzpp	0.079	0.250	0.045	0.203	0.336
cu_i_tpyaso	0.070	0.131	0.052	0.108	0.166
cu_i_tpym	0.322	0.301	0.051	0.110	0.146
cu_i_tpypo	0.074	0.133	0.054	0.108	0.138
cu_i_tpyps	0.433	0.176	0.293	0.261	0.180
cu_i_tpypse	0.310	0.148	0.308	0.279	0.135
cu_i_ttpp	0.056	0.200	0.031	0.161	0.270

cu_i_ttppo	0.048	0.184	0.032	0.126	0.359
cu_cn_ttpp	0.130	0.228	0.106	0.213	0.317
cu_scn_ttpp	0.084	0.267	0.051	0.233	0.348
cu_sph_dmpzpp	0.028	0.236	0.020	0.129	0.519
cu_ccph_ttpp	0.133	0.241	0.105	0.217	0.378
cu_ccnph_ttpp	0.476	0.779	0.425	0.842	0.989
cu_ccpyr_ttpp	0.132	0.276	0.104	0.259	0.478

Table S18. $\Delta E(S_1\text{-}T_1)$ energy differences using the isolated models. Entries highlighted in yellow indicate negative values and entries highlighted in orange exceed the 0.37 eV threshold.

	Isolated				
	PBE0	LRC-ωPBEh	B3LYP	CAM-B3LYP	ωB97X
cu_pop_pypz	0.205	0.282	0.159	0.253	0.272
cu_pop_pympz	0.175	0.263	0.139	0.240	0.284
cu_pop_pytfmpz	0.214	0.174	0.181	0.271	0.331
cu_pop_bpy	0.233	0.293	0.187	0.265	-0.027
cu_pop_mbpy	0.211	0.260	0.172	0.235	0.269
cu_pop_dmbpy	0.195	0.060	0.164	0.227	0.225
cu_pop_etbpy	0.205	0.259	0.129	0.175	0.218
cu_pop_phbpy	0.201	0.251	0.135	0.211	0.190
cu_pop_tfmbpy	0.197	0.274	0.166	0.199	0.230
cu_pop_55dtfmbpy	0.256	0.321	0.185	0.273	0.314
cu_pop_44dtfmbpy	0.261	0.317	0.199	0.284	0.320
cu_xantphos_tfmbpy	0.246	0.262	0.209	0.254	0.278
cu_xantphos_phbpy	0.212	0.268	0.149	0.231	0.228
cu_xantphos_dmbpy	0.222	0.010	0.183	0.240	0.279
cu_xantphos_44dtfmbpy	0.261	0.326	0.204	0.294	0.311
cu_xantphos_bpy	0.236	0.296	0.184	0.257	0.259
cu_xantphos_etbpy	0.267	0.267	0.158	0.210	0.232
cu_xantphos_mbpy	0.271	0.271	0.184	0.247	0.275
cu2_cl2_dpmb2	0.133	0.158	-0.192	0.150	0.125
cu2_cl2_dpmt2	0.138	0.141	0.041	0.167	0.604
cu2_cl2_dppb2	0.009	0.084	-0.156	0.032	0.805
cu2_cl2_pnme22	0.060	0.162	-0.003	0.080	0.419
cu2_br2_dpmb2	0.018	0.090	-0.208	0.054	0.105
cu2_br2_dpmt2	0.140	0.371	0.006	0.382	0.678
cu2_br2_dppb2	0.014	0.089	-0.150	0.052	0.022
cu2_br2_pnme22	0.045	0.110	-0.048	-0.022	0.316
cu2_i2_dpmb2	0.079	0.123	-0.236	0.082	0.170
cu2_i2_dpmt2	0.104	0.464	0.003	0.450	0.759
cu2_i2_dppb2	0.083	0.080	-0.156	0.071	0.044
cu2_i2_pnme22	0.042	0.062	-0.058	0.091	0.323
cu2_i2_pnpy22	0.148	0.447	-0.134	0.142	0.381
cu2_i2_phen2	0.130	0.238	0.323	0.147	0.197
cu2_i2_dmp2	0.240	0.223	0.296	0.218	0.193
cu2_i2_dppt12	0.108	0.277	-0.153	0.247	0.623
cu2_i2_dppt22	-0.006	0.094	-0.181	0.103	0.459
cu2_scn2_dppb2	0.024	0.124	-0.165	0.049	0.581
cu2_scn2_pop2	0.031	0.320	-0.121	0.321	0.863
cu_br_dmpzpp	0.115	0.192	-0.007	0.002	0.052
cu_br_tpypo	0.131	0.170	0.107	0.149	0.158

cu_br_ttpp	0.073	0.168	0.037	0.120	0.305
cu_cl_tpypo	0.134	0.165	0.111	0.146	0.151
cu_cl_ttpp	0.079	0.160	0.050	0.115	0.318
cu_i_dmpzpp	0.034	0.191	-0.016	0.070	0.200
cu_i_tpyaso	0.108	0.166	0.079	0.139	0.166
cu_i_tpym	0.119	0.172	0.094	0.144	0.155
cu_i_tpypo	0.111	0.169	0.086	0.143	0.164
cu_i_tpyps	0.113	0.170	0.088	0.142	0.165
cu_i_tpypse	0.113	0.169	0.088	0.145	0.168
cu_i_ttpp	0.065	0.181	0.017	0.108	0.315
cu_i_ttppo	0.053	0.134	0.005	0.059	0.112
cu_cn_ttpp	0.070	0.183	0.061	0.120	0.318
cu_scn_ttpp	0.058	0.164	0.020	0.109	0.130
cu_sph_dmpzpp	-0.001	0.129	-0.056	0.031	0.080
cu_ccph_ttpp	0.061	-0.181	0.036	0.100	0.341
cu_ccnph_ttpp	-0.026	0.373	-0.015	0.452	0.690
cu_ccpyr_ttpp	0.069	-0.128	0.034	0.107	0.327

Table S19. $\Delta E(S_1\text{-}T_1)$ energy differences using the QM/MM models. Entries highlighted in yellow indicate negative values and entries highlighted in orange exceed the 0.37 eV threshold.

	QM/MM				
	PBE0	LRC-ωPBEh	B3LYP	CAM-B3LYP	ωB97X
cu_pop_pypz	0.202	0.274	0.171	0.260	0.304
cu_pop_pympz	0.199	0.274	0.165	0.259	0.303
cu_pop_pytfmpz	0.263	0.316	0.221	0.298	0.338
cu_pop_bpy	0.228	0.264	0.215	0.198	0.173
cu_pop_mbpy	0.216	0.243	0.178	0.224	0.238
cu_pop_dmbpy	0.300	0.118	0.248	0.282	0.292
cu_pop_etbpy	0.205	0.015	0.168	0.208	0.226
cu_pop_phbpy	0.215	0.259	0.170	0.236	0.245
cu_pop_tfmbpy	0.244	0.297	0.188	0.273	0.299
cu_pop_55dtfmbpy	0.227	0.258	0.185	0.232	0.238
cu_pop_44dtfmbpy	0.248	0.291	0.207	0.265	0.280
cu_xantphos_tfmbpy	0.252	0.250	0.220	0.247	0.253
cu_xantphos_phbpy	0.356	0.271	0.317	0.260	0.283
cu_xantphos_dmbpy	0.204	0.232	0.155	0.207	0.214
cu_xantphos_44dtfmbpy	0.454	0.299	0.401	0.509	0.287
cu_xantphos_bpy	0.249	0.298	0.206	0.276	0.303
cu_xantphos_etbpy	0.240	0.243	0.195	0.249	0.263
cu_xantphos_mbpy	0.243	0.255	0.210	0.247	0.264
cu2_cl2_dpmb2	0.161	0.255	0.111	0.175	0.234
cu2_cl2_dpmt2	0.111	0.331	0.076	0.353	0.586
cu2_cl2_dppb2	0.134	0.165	0.100	0.138	0.134
cu2_cl2_pnme22	0.098	0.160	0.060	0.130	0.176
cu2_br2_dpmb2	0.159	0.259	0.097	0.160	0.247
cu2_br2_dpmt2	0.143	0.368	0.099	0.410	0.700
cu2_br2_dppb2	0.114	0.210	0.069	0.164	0.208
cu2_br2_pnme22	0.147	0.199	0.106	0.217	0.279
cu2_i2_dpmb2	0.192	0.203	0.173	0.247	0.324
cu2_i2_dpmt2	0.162	0.466	0.118	0.510	0.790
cu2_i2_dppb2	0.155	0.267	0.112	0.216	0.253
cu2_i2_pnme22	0.110	0.182	0.075	0.162	0.515
cu2_i2_pnpy22	0.136	0.203	0.109	0.188	0.640

cu2_i2_phen2	0.100	0.240	0.070	0.235	0.232
cu2_i2_dmp2	0.230	0.192	0.147	0.154	0.165
cu2_i2_dppt12	0.119	0.377	0.085	0.383	0.709
cu2_i2_dppt22	0.083	0.188	0.048	0.161	0.458
cu2_scn2_dppb2	0.086	0.124	0.065	0.102	0.119
cu2_scn2_pop2	0.023	0.668	-0.077	0.558	0.948
cu_br_dmpzpp	0.108	0.205	0.058	0.164	0.236
cu_br_tpypo	0.134	0.171	0.113	0.153	0.162
cu_br_ttpp	0.103	0.160	0.071	0.124	0.155
cu_cl_tpypo	0.138	0.169	0.116	0.151	0.160
cu_cl_ttpp	0.104	0.158	0.071	0.121	0.149
cu_i_dmpzpp	0.088	0.187	0.055	0.146	0.212
cu_i_tpyaso	0.106	0.163	0.079	0.135	0.160
cu_i_tpym	0.125	0.180	0.101	0.156	0.170
cu_i_tpypo	0.110	0.165	0.084	0.138	0.160
cu_i_tpyps	0.116	0.183	0.082	0.150	0.178
cu_i_tpypse	0.113	0.170	0.090	0.147	0.171
cu_i_ttpp	0.092	0.159	0.063	0.125	0.163
cu_i_ttppo	0.073	0.165	0.015	0.134	0.230
cu_cn_ttpp	0.110	0.162	0.080	0.133	0.160
cu_scn_ttpp	0.086	0.163	0.023	0.110	0.140
cu_sph_dmpzpp	0.022	0.170	-0.058	0.082	0.227
cu_ccph_ttpp	0.121	0.174	0.097	0.147	0.173
cu_ccnph_ttpp	0.175	0.546	0.165	0.620	0.825
cu_ccpyr_ttpp	0.125	0.065	0.095	0.133	0.162

References

1. Chen, X. L.; Yu, R. M.; Zhang, Q. K.; Zhou, L. J.; Wu, C. Y.; Zhang, Q.; Lu, C. Z. Rational Design of Strongly Blue-Emitting Cuprous Complexes with Thermally Activated Delayed Fluorescence and Application in Solution-Processed OLEDs. *Chemistry of Materials* **2013**, *25*, 3910-3920. DOI: 10.1021/cm4024309.
2. Keller, S.; Brunner, F.; Junquera-Hernández, J. M.; Pertegás, A.; La-Placa, M. G.; Prescimone, A.; Constable, E. C.; Bolink, H. J.; Ortí, E.; Housecroft, C. E. CF3 Substitution of Cu(PP)(bpy) PF6 Complexes: Effects on Photophysical Properties and Light-Emitting Electrochemical Cell Performance. *Chempluschem* **2018**, *83*, 217-229. DOI: 10.1002/cplu.201700501.
3. Costa, R. D.; Tordera, D.; Ortí, E.; Bolink, H. J.; Schönle, J.; Graber, S.; Housecroft, C. E.; Constable, E. C.; Zampese, J. A. Copper(I) complexes for sustainable light-emitting electrochemical cells. *Journal of Materials Chemistry* **2011**, *21*, 16108-16118. DOI: 10.1039/c1jm12607e.
4. Keller, S.; Constable, E. C.; Housecroft, C. E.; Neuburger, M.; Prescimone, A.; Longo, G.; Pertegás, A.; Sessolo, M.; Bolink, H. J. Cu(bpy)(P∧P)+ containing light-emitting electrochemical cells: improving performance through simple substitution. *Dalton Transactions* **2014**, *43*, 16593-16596. DOI: 10.1039/c4dt02847c.
5. Andrés-Tomé, I.; Fyson, J.; Dias, F. B.; Monkman, A. P.; Iacobellis, G.; Coppo, P. Copper(I) complexes with bipyridyl and phosphine ligands: a systematic study. *Dalton Transactions* **2012**, *41*, 8669-8674. DOI: 10.1039/c2dt30698k.
6. Keller, S.; Pertegás, A.; Longo, G.; Martínez, L.; Cerdá, J.; Junquera-Hernández, J. M.; Prescimone, A.; Constable, E. C.; Housecroft, C. E.; Ortí, E.; Bolink, H. J. Shine bright or live long: substituent effects in Cu(N ∧ N)(P ∧ P) +-based light-emitting

electrochemical cells where N ∧ N is a 6-substituted 2,2'-bipyridine. *Journal of Materials Chemistry C* **2016**, *4*, 3857-3871. DOI: 10.1039/c5tc03725e.
7. Brunner, F.; Babaei, A.; Pertegás, A.; Junquera-Hernández, J. M.; Prescimone, A.; Constable, E. C.; Bolink, H. J.; Sessolo, M.; Ortí, E.; Housecroft, C. E. Phosphane tuning in heteroleptic Cu(NN)(PP)+ complexes for light-emitting electrochemical cells. *Dalton Transactions* **2019**, *48*, 446-460. DOI: 10.1039/c8dt03827a.
8. Hong, X.; Wang, B.; Liu, L.; Zhong, X. X.; Li, F. B.; Wang, L.; Wong, W. Y.; Qin, H. M.; Lo, Y. H. Highly efficient blue-green neutral dinuclear copper(I) halide complexes containing bidentate phosphine ligands. *Journal of Luminescence* **2016**, *180*, 64-72. DOI: 10.1016/j.jlumin.2016.08.004.
9. Wei, Q.; Chen, H. T.; Liu, L.; Zhong, X. X.; Wang, L.; Li, F. B.; Cong, H. J.; Wong, W. Y.; Alamry, K. A.; Qin, H. M. Syntheses and photoluminescence of copper(i) halide complexes containing dimethylthiophene bidentate phosphine ligands. *New Journal of Chemistry* **2019**, *43*, 13408-13417. DOI: 10.1039/c9nj01417a.
10. Tsuboyama, A.; Kuge, K.; Furugori, M.; Okada, S.; Hoshino, M.; Ueno, K. Photophysical properties of highly luminescent copper(I) halide complexes chelated with 1,2-bis(diphenylphosphino)benzene. *Inorganic Chemistry* **2007**, *46*, 1992-2001. DOI: 10.1021/ic0608086.
11. Leitl, M. J.; Küchle, F. R.; Mayer, H. A.; Wesemann, L.; Yersin, H. Brightly Blue and Green Emitting Cu(I) Dimers for Singlet Harvesting in OLEDs. *Journal of Physical Chemistry A* **2013**, *117*, 11823-11836. DOI: 10.1021/jp402975d.
12. Nitsch, J.; Kleeberg, C.; Fröhlich, R.; Steffen, A. Luminescent copper(I) halide and pseudohalide phenanthroline complexes revisited: simple structures, complicated excited state behavior. *Dalton Transactions* **2015**, *44*, 6944-6960. DOI: 10.1039/c4dt03706e.
13. Li, X.; Zhang, J.; Zhao, Z.; Yu, X.; Li, P.; Yao, Y.; Liu, Z.; Jin, Q.; Bian, Z.; Lu, Z.; Huang, C. Bluish-Green Cu(I) Dimers Chelated with Thiophene Ring-Introduced Diphosphine Ligands for Both Singlet and Triplet Harvesting in OLEDs. *Acs Applied Materials & Interfaces* **2019**, *11*, 3262-3270. DOI: 10.1021/acsami.8b15897.
14. Chakkaradhari, G.; Eskelinen, T.; Degbe, C.; Belyaev, A.; Melnikov, A. S.; Grachova, E. V.; Tunik, S. P.; Hirva, P.; Koshevoy, I. O. Oligophosphine-thiocyanate Copper(I) and Silver(I) Complexes and Their Borane Derivatives Showing Delayed Fluorescence. *Inorganic Chemistry* **2019**, *58*, 3646-3660. DOI: 10.1021/acs.inorgchem.8b03166.
15. Klein, M.; Rau, N.; Wende, M.; Sundermeyer, J.; Cheng, G.; Che, C. M.; Schinabeck, A.; Yersin, H. Cu(I) and Ag(I) Complexes with a New Type of Rigid Tridentate N,P,P-Ligand for Thermally Activated Delayed Fluorescence and OLEDs with High External Quantum Efficiency. *Chemistry of Materials* **2020**, *32*, 10365-10382. DOI: 10.1021/acs.chemmater.0c02683.
16. Gneuss, T.; Leitl, M. J.; Finger, L. H.; Rau, N.; Yersin, H.; Sundermeyer, J. A new class of luminescent Cu(I) complexes with tripodal ligands - TADF emitters for the yellow to red color range. *Dalton Transactions* **2015**, *44*, 8506-8520. DOI: 10.1039/c4dt02631d.
17. Zhang, J.; Duan, C. B.; Han, C. M.; Yang, H.; Wei, Y.; Xu, H. Balanced Dual Emissions from Tridentate Phosphine-Coordinate Copper(I) Complexes toward Highly Efficient Yellow OLEDs. *Advanced Materials* **2016**, *28*, 5975. DOI: 10.1002/adma.201600487.
18. Dau, T. M.; Asamoah, B. D.; Belyaev, A.; Chakkaradhari, G.; Hirva, P.; Jänis, J.; Grachova, E. V.; Tunik, S. P.; Koshevoy, I. O. Adjustable coordination of a hybrid

phosphine-phosphine oxide ligand in luminescent Cu, Ag and Au complexes. *Dalton Transactions* **2016**, *45*, 14160-14173. DOI: 10.1039/c6dt02435a.

19. Chakkaradhari, G.; Chen, Y. T.; Karttunen, A. J.; Dau, M. T.; Jänis, J.; Tunik, S. P.; Chou, P. T.; Ho, M. L.; Koshevoy, I. O. Luminescent Triphosphine Cyanide d10 Metal Complexes. *Inorganic Chemistry* **2016**, *55*, 2174-2184. DOI: 10.1021/acs.inorgchem.5b02581.
20. Chakkaradhari, G.; Belyaev, A. A.; Karttunen, A. J.; Sivchik, V.; Tunik, S. P.; Koshevoy, I. O. Alkynyl triphosphine copper complexes: synthesis and photophysical studies. *Dalton Transactions* **2015**, *44*, 13294-13304. DOI: 10.1039/c5dt01870f.
